# Dual‐State Photophysical Modulation via Bifurcated Hydrogen Bonding in a U‐Shaped Dipyridophenazine‐Cored Donor‐π‐Acceptor‐π‐Donor Fluorophore

**DOI:** 10.1002/chem.202503421

**Published:** 2025-12-26

**Authors:** Kimiya Takei, Shunsuke Kobashi, Divya, Norimitsu Tohnai, Satoshi Minakata, Yoichi Kobayashi, Piotr de Silva, Youhei Takeda

**Affiliations:** ^1^ Department of Applied Chemistry Graduate School of Engineering The University of Osaka Suita Osaka Japan; ^2^ Department of Applied Chemistry College of Life Sciences Ritsumeikan University Kusatsu Shiga Japan; ^3^ Department of Energy Conversion and Storage Technical University of Denmark Kongens Lyngby Denmark

**Keywords:** charge‐transfer, donor‐acceptor, hydrogen bonding, luminescence, supramolecular complex

## Abstract

Modulating photophysical properties via hydrogen bonding offers a powerful strategy for designing supramolecular functional materials and responsive optical systems. Here, we present a U‐shaped dipyridophenazine (DPyPHZ)‐cored donor–π–acceptor–π–donor (D–π–A–π–D) scaffold that serves as a hydrogen‐bond receptor capable of forming bifurcated hydrogen bonds, enabling simultaneous modulation of both ground and excited states. We developed an efficient synthetic route to this π‐conjugated system, and demonstrated that its absorption and emission spectra undergo pronounced red‐shifts upon 1:1 complexation with neutral hydrogen‐bond donors such as sulfonamides and even water. Spectroscopic analyses reveal that the hydrogen‐bonding interaction stabilizes the charge‐transfer excited state, leading to enhanced photoluminescence quantum yield (PLQY) and color modulation. Notably, these optical responses persist in the solid state when embedded in a polymer matrix, enabling emission color switching without fluorophore aggregation. Single‐crystal X‐ray analysis confirmed the formation of a well‐defined bifurcated hydrogen‐bonding complex in the solid state. Comparative studies with structural analogues highlight the importance of the electron‐density hotspot in DPyPHZ for selective and directional hydrogen bonding. This work introduces a supramolecular design strategy for precise dual‐state photophysical control, providing a platform for environmentally responsive luminescent materials with potential in sensing, imaging, and optoelectronic applications.

## Graphical abstract

A donor–π–acceptor–π–donor (D–π–A–π–D) fluorophore bearing a U‐shaped dipyridophenazine core forms bifurcated hydrogen bonds with neutral donors such as sulfonamides or water. This interaction modulates both ground and excited states, enabling red‐shifted emission and enhanced photoluminescence in solution and solid state.

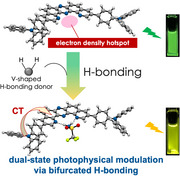

## 1 Introduction

Hydrogen bonding is a ubiquitous noncovalent interaction in nature, playing essential roles in genetic information storage and decoding through base pairing in nucleic acids [1], protein folding [2], and enzymatic catalysis [3]. Inspired by nature's elegant control of molecular recognition, synthetic hydrogen‐bonding motifs have been widely explored to construct functional supramolecular architectures [4]. From a materials science perspective, elucidating how hydrogen bonding governs the photophysical behavior of organic fluorophores is of particular significance. Precise modulation of emission properties via hydrogen bonding offers a powerful strategy for advancing environmental sensing, bioimaging, and energy‐efficient photonic technologies.

A particularly promising mechanism for hydrogen‐bond‐assisted photophysical modulation is excited‐state proton transfer (ESPT). For example, intermolecular hydrogen bonding between heteroaromatic fluorophores and protic solvents such as alcohols often results in luminescence quenching due to enhanced nonradiative internal conversion [5, 6]. Conversely, intramolecular ESPT (ESIPT) typically induces excited‐state tautomerization, producing emission with a large Stokes shift from the stabilized tautomeric form [7]. This ESPT‐driven behavior has enabled ON/OFF emission switching and sizable shifts in emission wavelength, driving the development of ESIPT‐based sensing and imaging probes [8]. Despite these advances, direct modulation of both ground‐ and excited‐state electronic structures through hydrogen bonding remains largely underexplored [9]. Most known systems exhibit only subtle shifts due to weak binding affinities or poor orbital matching between donor and acceptor sites [9]. Unlocking dual‐state modulation could offer a complementary strategy to established ESPT‐based approaches, paving the way toward next‐generation functional luminescent materials.

**FIGURE 1:**
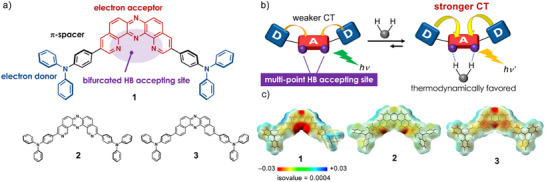
(a) Molecular structures of the designed compound **1** and reference compounds **2** and **3**; (b) Direct modulation of both the ground‐ and excited‐state electronic structures in a fluorophore consisting of electron donor and acceptor units; (c) Electrostatic potential map (EPM) of compounds **1–3**.

Herein, we disclose the design and synthesis of a D‐π‐A‐π‐D type fluorophore (compound **1**) that exhibits hydrogen‐bond‐responsive photophysical behavior (Figure 1a). To rigorously evaluate the effect of molecular design, two structural analogues (**2** and **3**) were also synthesized for comparison (Figure 1a). Notably, D‐π‐A‐π‐D compound **1** forms 1:1 supramolecular complexes with V‐shaped hydrogen bond donors— including sulfonamides and carboxylic amides and even water—resulting in pronounced red shifts in both UV‐vis absorption and photoluminescence (PL) spectra. These spectral modulations arise from enhanced charge‐transfer (CT) character in both ground and excited states, enabled by a unique bifurcated hydrogen‐bonding geometry (Figure 1b). Remarkably, the hydrogen‐bonding effect persists in polymer‐dispersed solid films, highlighting the robustness of this supramolecular design strategy.

## 2 Results and Discussion

To achieve simultaneous modulation of both ground‐ and excited‐state electronic structures via hydrogen bonding, it is essential to enhance the hydrogen bond‐accepting ability of the fluorophore and to control the directionality of electron‐density redistribution with precision. We hypothesized that an electron donor (D)–acceptor (A)–donor (D–A–D) framework bearing a multi‐point hydrogen bond‐accepting site at the acceptor unit would meet these criteria (Figure 1b). In twisted D–A systems, the D and A units are partially electronically coupled in both ground and exited states, typically resulting in CT emission as the dominant photo‐radiative process. When such a fluorophore incorporates multiple hydrogen bond‐accepting sites at the acceptor unit, the formation of multi‐point hydrogen bonds can shift the associate‐dissociation equilibrium toward the hydrogen‐bonded complex, thereby stabilizing the CT state. To realize this concept, we designed compound **1**, a D‐π‐A‐π‐D‐type molecule in which the acceptor unit plays a pivotal role (Figure 1a). As the electron acceptor, we employed a kinked (U‐shaped) tetraazaacene core, dipyridophenazine (DPyPHZ), which features four sp^2^‐hybridized nitrogen atoms embedded within a π‐conjugated scaffold. This configuration generates a localized electron‐density hotspot (Figure 1c). In contrast, regioisomeric compound **2**, which contains two sp^2^‐hybridized nitrogen atoms at alternative positions, and analogue **3**, which lacks such nitrogen atoms, exhibit more diffuse electron distributions (Figure 1c). The highly localized electron‐rich site in **1** enables the formation of strong, geometry‐matched bifurcated bonds with suitable hydrogen‐bond donors, favoring a thermodynamically stable association. To further amplify CT character and its associated photophysical effects, the acceptor unit is flanked on both sides by strong electron donors, connected via phenylene π‐spacers (Figure 1a). This architecture promotes efficient mixing of CT and locally excited (π‐π*) states, thereby enhancing the PLQY.

**SCHEME 1:**
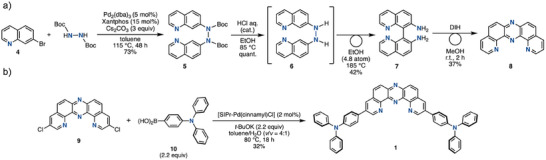
Synthetic routes of a) **8** and b) **1**.

As an initial step, we explored synthetic access to the key acceptor molecule **8** (Scheme 1a and Eq. S1–S3). While DPyPHZ has previously been used as a tridentate ligand in transition‐metal coordination chemistry [10], its application as an electron‐accepting or hydrogen‐bonding synthon has not been reported. The original synthesis of DPyPHZ **8** by Case et al. in 1966 [11], relied on harsh, multi‐step conditions, including phenazine nitration with concentrated and fuming H_2_SO_4_/HNO_3_ mixtures, followed by recrystallization, nitro group reduction, and sequential Skraup reactions. To streamline access to DPyPHZ and its derivatives, we explored a more efficient strategy based on oxidative rearrangement of binaphthalene diamines, mediated by an iodine‐containing oxidant [12]. As shown in Scheme 1a, 8,8’‐biquinoline‐7,7’‐diamine (**7**) was synthesized via a Pd‐catalyzed diamination of 7‐bromoquinoline (**4**) with *N,N’*‐diBoc‐hydrazine. Subsequent Boc deprotection of intermediate **5**, followed by a [3,3]‐sigmatropic rearrangement of diaryl hydrazine **6**, led to tautomerization and afforded diamine **7** in high yield. Upon exposure of diamine **7** to the oxidative skeletal rearrangement conditions [12], the desired DPyPHZ **8** was obtained in good yield (Scheme 1a). Using this synthetic route, we further prepared 3,11‐dichloro‐substituted DPyPHZ derivative **9** from 7‐bromo‐3‐chloroquinoline (see Eq. S4–S7). The target compound **1** was then successfully synthesized via a Pd‐catalyzed Suzuki–Miyaura double cross‐coupling of **9** with 4‐(*N,N*‐diphenylamino)phenylboronic acid (**10**) (Scheme 1b and Eq. S8). Reference compounds **2** and **3** were synthesized in a similar manner (Eq. S9–S13).

**FIGURE 2:**
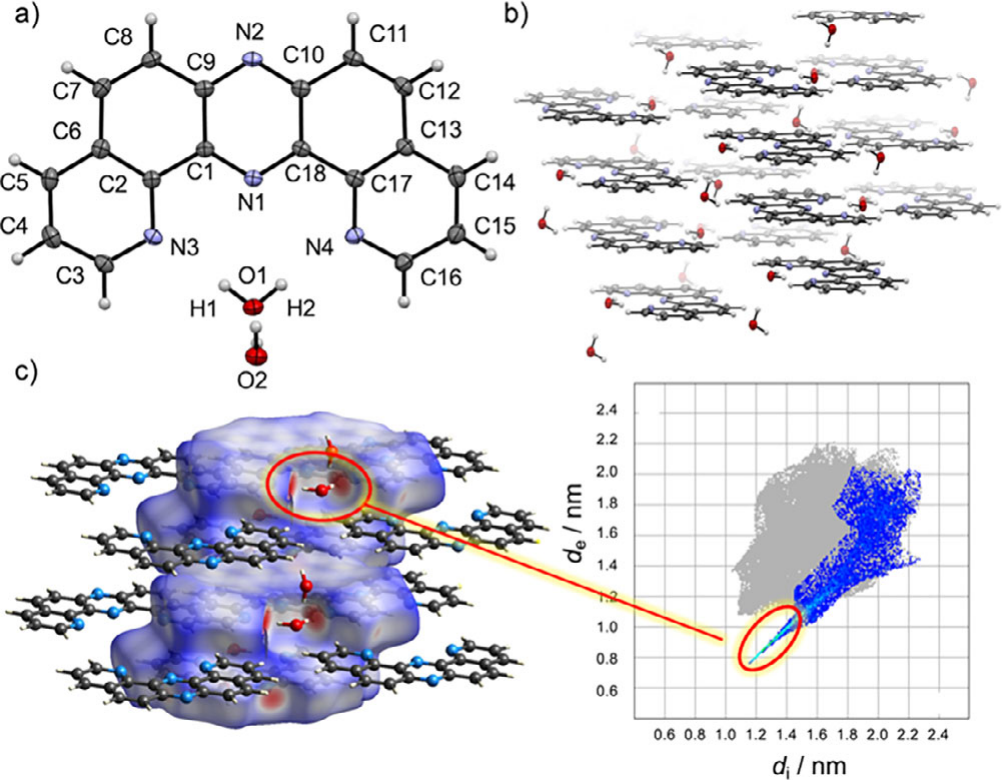
(a) Molecular structure of **8** in a single crystal grown from methanol. Thermal ellipsoids are set at the 50% probability level. Selected geometry parameters for **8**: N3•••O1 = 2.89(7) Å; N4•••O1 = 2.93(7) Å; O1•••O2 = 2.77(1) Å; N3–H1–O1 = 174.9(5)°; N4–H2–O1 = 175.2(2)°; (b) Packing diagram and (c) Hirshfeld surface analysis of **8** in the single crystal. The inset indicates a 2D fingerprint plot, highlighting N•••H/H•••N contacts.

A colorless single crystal of DPyPHZ **8**, suitable for X‐ray diffraction analysis, was obtained by slow evaporation from a MeOH solution (Figure 2a and Table S1) [13]. Unexpectedly, the crystal was identified as a sesquihydrate, with a water molecule located within the inner cleft of the DPyPHZ framework, presumably originating from the adventitious water in the MeOH solvent. The observed N•••O distances (N3•••O1 = 2.89(7) Å and N4•••O1 = 2.93(7) Å) are shorter than the sum of the van der Waals radii of nitrogen and oxygen (3.07 Å), indicative of a significant interaction. Moreover, the nearly linear O─H•••N angles [N3─H1─O1 = 174.9(5)°; N4─H2─O1 = 175.2(2)°] suggest moderately strong, directionally defined hydrogen bonds, primarily of electrostatic character [14]. The hydrogen‐bonded water molecule bridges two DPyPHZ units, forming additional hydrogen bonds with both the sp^2^‐hybrized nitrogen atoms and neighboring water molecules (Figure 2a). The DPyPHZ molecules adopt an antiparallel π‐stacked arrangement with an interplanar distance of 3.40(8) Å (Figure 2b), closely matching the van der Waals contact distance between aromatic carbon atoms (3.40 Å), thus implying favorable π‐π interactions. Hirshfeld surface analysis [15] clearly visualizes the bifurcated hydrogen bonding interactions between water molecules and the two sp^2^ nitrogen atoms, as highlighted by prominent red regions (Figure 2c). This finding underscores the inherent ability of the DPyPHZ core to act as a geometrically defined bifurcated hydrogen‐bond acceptor. The corresponding 2D fingerprint plot, generated from internal (*d*_i_) and external (*d*_e_) distances relative to the Hirshfeld surface, reveals a distinct spike in the 1.2–1.4 Å range, consistent with short N•••H contacts indicative of strong hydrogen bonding (inset in Figure 2c).

**FIGURE 3:**
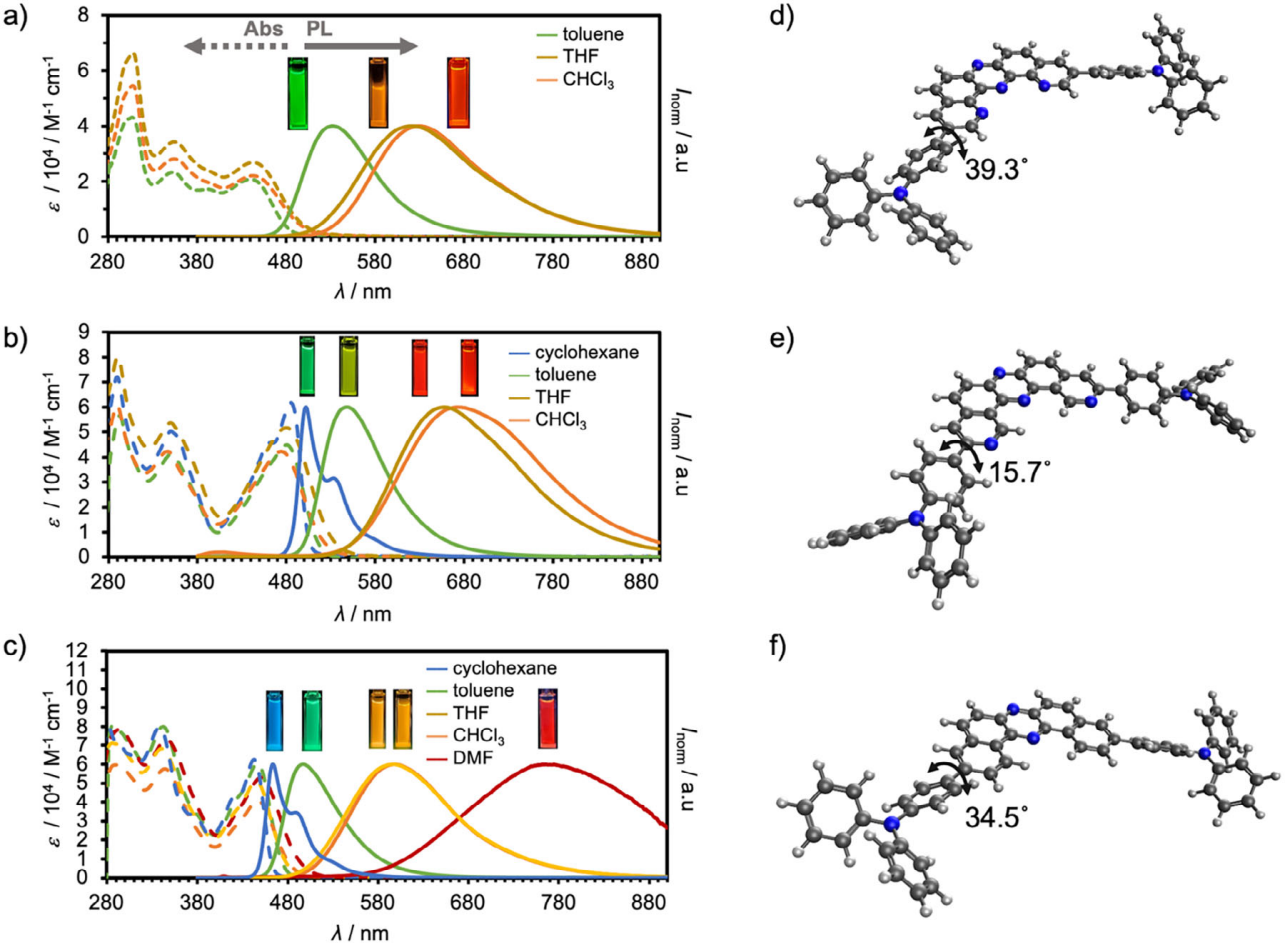
Steady‐state UV‐vis and PL spectra of diluted solutions (*c* = 10^−5^ M) of (a) **1**, (b) **2**, and (c) **3**. Excited at *λ*_ex_ = 365 nm. The solubilities of compounds **1** and **2** were not enough to make homogeneous solutions in cyclohexane and DMF. The inset photographs show PL of each solution under the irradiation of a UV lamp (*λ*_ex_ = 365 nm). Ground state optimized structures of (d) **1**, (e) **2**, and (f) **3** in toluene at PBE0/6‐31G(d,p) level.

As the initial step in our photophysical investigation, we evaluated the fundamental optical properties of the D–π–A–π–D compounds **1–3** in diluted solutions (*c* = 10^−5^ M) (Figure 3, Table 1, Figures S1–S6, and Tables S2–S4). In toluene, compound **1** displayed a broad absorption band centered at approximately 440 nm, which remained largely unaffected by solvent polarity (Figure 3a and Table S2). As designed, compound **1** exhibited distinct PL (*λ*_ex_ = 365 nm) characterized by a Gaussian‐shaped emission profile, indicative of a CT excited state (Figure 3a). In contrast to its absorption spectra, the emission maximum (*λ*_PL_) exhibited strong solvent dependence, shifting from green (*λ*_PL_ = 532 nm in toluene) to orange (*λ*_PL_ = 629 nm in CHCl_3_) (Table 1 and Table S2). Such positive solvatochromic behavior is a hallmark of donor–acceptor–donor (D–A–D) architectures [16]. Notably, compound **1** maintained a high PLQY in the range of 0.62–0.76, regardless of solvent polarity (Table 1 and Table S2), which is likely due to the effective mixing of CT and locally excited (π‐π*) states mediated by the phenylene π‐spacer, resulting in enhanced oscillator strength.

**TABLE 1:** Summary of photophysical properties of toluene solution (*c* = 10^−5^ M) of **1–3**.

Compd.	*λ*_abs_ [nm]	*ε* [M^−1^cm^−1^]	*λ*_em_ [nm]^[^ ^a^ ^]^	*Φ*_PL_^[^ ^b^ ^]^	<*τ*> [ns]^[^ ^c^ ^]^	*k_f_* [s^−1^]^[^ ^d^ ^]^
**1**	446	20500	532	0.76	4.10	1.85×10^8^
**2**	480	44800	547	0.75	3.34	2.25×10^8^
**3**	445	60300	498	0.74	2.87	2.58×10^8^

^[a]^ Excited at *λ*_ex_ = 365 nm.

^[b]^ The absolute PLQY acquired with an integral sphere.

^[c]^ Intensity average lifetime.

^[d]^ fluorescence rate *k*_f_ = *Φ*_PL_/<*τ*>.

Reference compounds **2** and **3** exhibited similar CT‐type emission (*λ*_ex_ = 365 nm) behavior with solvent‐dependent red shifts (Figures 3b, c, Table S3, and S4). However, comparative analysis of compounds **1–3** revealed key structural and photophysical differences (see Figure S7 for absorption and emission spectra in the same solvent). Compound **2** showed both absorption and PL spectra red‐shifted relative to **1** under identical solvent conditions (Figures 3a, 3b, and Table 1). This difference can be rationalized by examining the dihedral angles between the diphenylamino group and the phenylene spacer, as determined from optimized structures in toluene: 39.3° for **1**, 15.7° for **2**, and 34.5° for **3** (Figures 3d–f). The smaller dihedral angle in **2** suggests enhanced π‐conjugation, which lowers the HOMO–LUMO energy gap and red‐shifts the absorption and emission. This effect is further attributed to the substitution of a C─H bond at the 2‐position of the DBPHZ core with an sp^2^ nitrogen atom, which reduces steric hindrance in the biaryl linkage, allowing better orbital overlap. In contrast, when compared to compound **3**, compound **1** exhibited a markedly red‐shifted PL band, consistent with a more stabilized CT excited state (Figure 3a vs. 3c). This stabilization is in agreement with the higher electron affinity of the DPyPHZ acceptor core (3.71 eV, Figure S8) relative to that of the dibenzophenazine acceptor unit used in **3** (3.29 eV) [12].

**FIGURE 4:**
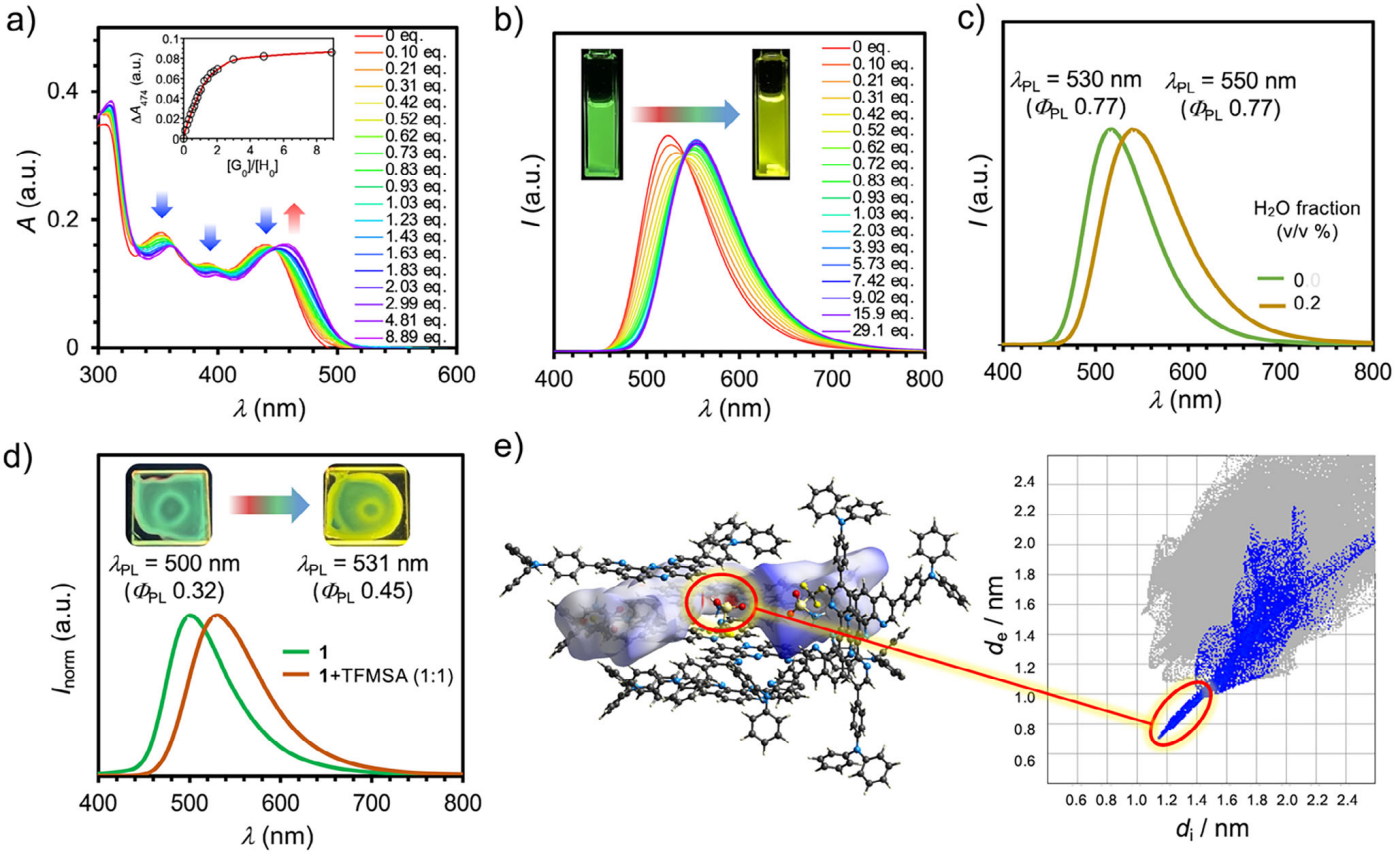
(a) UV‐vis absorption titration of **1** with TFMSA in toluene. The inserted graph indicates the isotherm plots obtained by plotting the absorbance at 474 nm as a function of [G]_0_/[H]_0_ ([H]_0_ = 0.52×10^–^^5^ M); (b) PL titration of **1** with TFMSA in toluene ([H]_0_ = 0.96×10^–^^5^ M;* λ*_ex_ = 365 nm). The inset photographs show PL of solution of **1** and **1** +TFMSA (29.1 eq.) under the irradiation of a UV lamp (*λ*_ex_ = 365 nm); (c) PL spectra of dried **1** in toluene (moth green line) and in wet (0.2 *v*/*v* water fraction) toluene (brown line) (*λ*_ex_ = 365 nm); (d) PL spectra of **1**@PS (1 wt%) and **1**+TFMSA@PS (*λ*_ex_ = 365 nm). The inset photographs show PL of film of **1**@PS and **1**+TFMSA@PS under the irradiation of a UV lamp (*λ*_ex_ = 365 nm); (e) Packing diagram of **1–TFMSA** (1:1 complex), along with its Hirshfeld surface analysis and 2D fingerprint plot, highlighting N•••H/H•••N contacts.

To validate the hydrogen bonding‐responsive photophysical properties of compound **1**, we selected trifluoromethanesulfonamide (TFMSA; p*K*_a_ = 9.7 in DMSO) [17] as a model hydrogen bond donor capable of forming bifurcated interactions. Upon addition of TFMSA to a dilute toluene solution of **1** (*c* = 1.09 × 10^−5^ M), the UV‐vis absorption spectrum exhibited a pronounced transformation (Figure 4a). Specifically, the absorption band centered around *λ*_abs_ = 440 nm underwent a noticeable red shift. A Job plot analysis revealed a maximum at a molar ratio of [**1**]_0_/([**1**]_0_+[TFMSA]) ∼ 0.5, consistent with the formation of 1:1 complex (Figure S9). Nonlinear least‐squares fitting of the titration isotherm to a 1:1 binding model yielded an association constant of *K* = (4.61±0.25)×10^5^ M^−1^ (inset, Figure 4a). In parallel with the absorption changes, the PL spectra of **1** exhibited a red shift upon increasing TFMSA concentration (Figure 4b), with the emission maximum shifting from *λ*_PL_ = 524 nm (green) to *λ*_PL_ = 554 nm (yellow). Concomitantly, the PLQY increased from 0.78 to 0.83, along with an increase in <*τ*> from 4.10 ns to 5.56 ns (Figures S10 and S11). To evaluate the specificity of this interaction, we performed analogous titrations using reference compounds **2** and **3** (Figure S12). In both cases, the UV‐vis spectra showed negligible changes (Figure S12a, b), and the PL spectra displayed no significant shift in *λ*_em_, although slight emission quenching was observed upon TFMSA addition (Figure S12c, d). These control experiments strongly suggest that the unique coordination geometry and electron‐density distribution of the DPyPHZ core in **1** are crucial for enabling effective photophysical modulation via bifurcated hydrogen bonding. Furthermore, the dual‐state modulation of **1—**in both ground and excited states—is attributable to this hydrogen‐bonding interaction. To investigate the effect of solvent environment on complexation, titration experiments were also performed in THF and CHCl_3_. The resulting association constants were significantly lower: *K* = (2.49±0.12)×10^3^ M^−1^ in THF and (6.58±0.60)×10^4^ M^−1^ in CHCl_3_ (Figure S13). These decreases can be rationalized by competitive hydrogen bonding between TFMSA and the coordinating solvent (in the case of THF) and by specific interactions between the acidic CHCl_3_ and **1**, both of which interfere with complex formation.

To assess the generality of dual‐state photophysical modulation, UV‐vis and PL titrations were performed using weaker hydrogen‐bond donors: methanesulfonamide (p*K*_a_ = 17.5 in DMSO), trifluoroacetoamide (p*K*_a_ = 17.3 in DMSO), and acetoamide (p*K*_a_ = 25.5 in DMSO). Notably, each hydrogen‐bond donor induced comparable red shifts in both absorption and PL spectra (Figure S14), and a clear inverse correlation between p*K*_a_ and log*K* was observed (Figure S15), consistent with classical hydrogen‐bonding thermodynamics.

To gain deeper insights into the association behavior between **1** and TFMSA, we monitored the ^1^H NMR spectra of **1** upon incremental addition of TFMSA in CDCl_3_ (Figure S16). In the absence of TFMSA, a signal attributed to water appeared at 2.03 ppm, markedly downfield from the typical water signal in CDCl_3_ (1.56 ppm). This shift suggests that trace amounts of water form hydrogen‐bonded complexes with **1**, undergoing rapid exchange with free water molecules. Upon addition of 0.08 equivalents of TFMSA, the signal corresponding to its acidic N–H protons shifted dramatically downfield, from 5.19 ppm to 11.89 ppm, indicating strong hydrogen bond formation (Figure S16). Concurrently, the signal assigned to the proton at the 2‐position of the DPyPHZ core shifted upfield, while the water signal moved further upfield as well. Given the significantly lower p*K*_a_ of water (31.4 in DMSO) relative to TFMSA (9.7), these observations suggest that water initially forms bifurcated hydrogen bonds with **1**, but is readily displaced by TFMSA, the stronger hydrogen bond donor. ^1^H NMR titration analysis using water as a guest yielded an association constant of *K* = (0.62±0.16) M^−1^ in CDCl_3_ (Figure S17). At the start of the titration ([H_2_O]_0_/[**1**]_0_ = 0.5), the H_2_O resonance appeared in the downfield region (3.53 ppm), indicating that the equilibrium strongly favors the hydrogen‐bonded (associated) form. As [H_2_O]_0_/[**1**]_0_ was increased, the water signal shifted significantly upfield, reaching a plateau at 1.71 ppm (Figure S17). Such an upfield shift is consistent with fast exchange on the NMR time scale between free and bound water, leading to a population‐weighted averaged chemical shift. In view of the relatively low binding constant and the fact that hydrogen bonding is largely electrostatic/polarization in nature, the chemical shift of the hydrogen nuclei at the C2 position of the DPyPHZ unit changed only marginally. Notably, even in the presence of excess water, both absorption and emission spectra of **1** exhibited red shifts without significant quenching (Figure 4c), further supporting that hydrogen bonding modulates its photophysical behavior in solution.

To further evaluate the robustness of our molecular design, we investigated the hydrogen‐bond‐mediated photophysical modulation of **1** in a solid‐state environment. A toluene solution of **1** and polystyrene (PS, 1 wt%) was spin‐coated onto a quartz plate and dried to afford a thin film (**1**@PS). The resulting film exhibited green PL (*λ*_PL_ = 500 nm) (Figure 4d). Remarkably, when a 1:1 mixture of **1** and TFMSA was used to prepare the film (**1**+TFMSA@PS), the emission underwent a substantial red shift to *λ*_PL_ = 531 nm, along with an increase in PLQY (Figures 4d and S18). In contrast, analogous films prepared using compounds **2** and **3** with TFMSA showed no discernible shift in emission (Figures S19 and S20), indicating that bifurcated hydrogen bonding remains operative in the solid state only for **1**. The ability to modulate emission color in response to external hydrogen‐bond donors in the solid state underscores the potential of **1** as a platform for smart optoelectronic materials, such as color‐tunable OLEDs [18].

To directly confirm the presence of a 1:1 hydrogen‐bonded complex in the solid state, we crystallized a mixture of **1** and TFMSA (1:1 molar ratio) from toluene. Single crystals suitable for X‐ray diffraction were successfully obtained [13]. The crystal structure revealed a well‐defined 1:1 complex stabilized by bifurcated hydrogen bonding, in accordance with our molecular design (Figure 4e). Interestingly, the complex adopts a dimeric packing motif, in which two DPyPHZ cores align in an anti‐parallel fashion with a π‐π stacking distance of 3.49 Å (Figure 4e), indicative of favorable intermolecular interactions.

**FIGURE 5:**
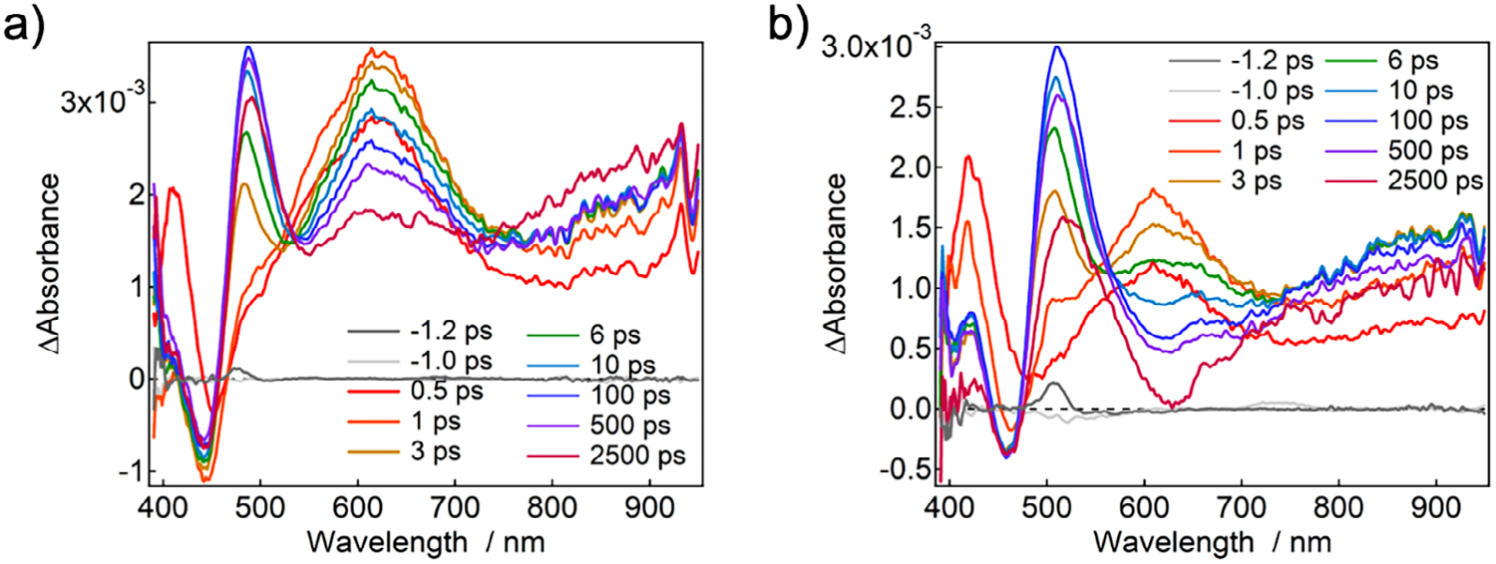
a) Transient absorption spectrum of **1** excited at 347 nm; b) Transient absorption spectrum of 1+TFMSA (1:TFMSA = 1:235) excited at 347 nm.

To gain more detailed insights into how TFMSA modulates the excited‐state dynamics of compound **1**, we performed femtosecond transient absorption (TA) measurements using a 347 nm excitation pulse (Figure 5a; Figures S21–S23). In toluene, the TA spectrum of **1** exhibited a negative signal at 450 nm, attributed to ground‐state bleach, and a transient absorption band at 615 nm, originating from the excited state S_1_, immediately after excitation. Within a few picoseconds, the 615 nm band partially decayed, accompanied by the growth of new transient absorption bands at 490 and 900 nm. Notably, a local minimum appeared at 550 nm, which exhibited a significant red shift in chloroform, consistent with solvent‐dependent shifts observed in the steady‐state fluorescence spectra.

This spectral dip at 550 nm is attributed to the overlap of transient absorption and stimulated emission. Furthermore, the picosecond dynamics were accelerated in a more polar solvent (i.e., chloroform), indicating that the initial excited state undergoes rapid revolution from a localized S_1_ state centered on the DPyPHZ core to a CT S_1_ state. The CT S_1_ state gradually decayed over a nanosecond timescale, while the transient absorption band at 900 nm continued to increase gradually. Microsecond transient absorption measurements revealed that this signal originates from the formation of the T_1_ state via intersystem crossing.

When TFMSA was added to **1**, the TA spectrum of **1** retained the initial transient absorption band at around 615 nm, indicative of the same localized S_1_ state (Figure 5b). However, the dip originating from stimulated emission shifts to ∼650 nm. The difference in stimulated emission indicates that the addition of TFMSA modulates the CT state. Furthermore, the rise component at 900 nm, associated with the formation of the T_1_ state in the nanosecond‐order decay, was not observed. The addition of TFMSA led to an increase in fluorescence quantum yield and a reduction in microsecond‐scale transient absorption signals, demonstrating that TFMSA suppresses intersystem crossing. Specifically, the calculated nonradiative decay rate constants [*k*_nr_ = (1–*Φ*_PL_)/<*τ*>] for compound **1** in toluene are approximately 5.85×10⁷ s^−^^1^ without TFMSA and 3.06×10⁷ s^−^^1^ with 6 equivalents of TFMSA. This significant reduction in *k*_nr_ supports our hypothesis that TFMSA suppresses nonradiative deactivation of the excited state. This conclusion is qualitatively supported by the theoretical calculations of intersystem crossing rate constants (Table S6). We observe that the fastest intersystem crossing channel in **1** is through the T_5_ state with a rate constant *k*_ISC_ = 1.8×10^9^ s^−^^1^. Upon the complexation with TFMSA, this rate constant is approximately halved leading to a higher concentration of emissive singlet states. The net effect is likely to be the results of an interplay between various nonradiative and radiative decay processes.

Compounds **2** and **3** exhibited similar excited‐state dynamics to **1**, which initially had transient absorption spectra features corresponding to the local S_1_ states, followed by evolution into CT S_1_ state on a picosecond timescale (Figures S24–S27). Global analyses using singular value decomposition revealed electron‐transfer time constants of 4.0, 5.6, and 3.2 ps for **1**, **2**, and **3** in toluene, respectively (Figures S28–S37). These time constants roughly correlate with the extent of Stokes shift observed in polar solvents (Figure 3a–3c). This trend indicates that the larger the CT character, the larger the Stokes shift of the CT emission, which in turn corresponds to a longer relaxation time to reach the post‐electron‐transfer stabilized structure.

**FIGURE 6:**
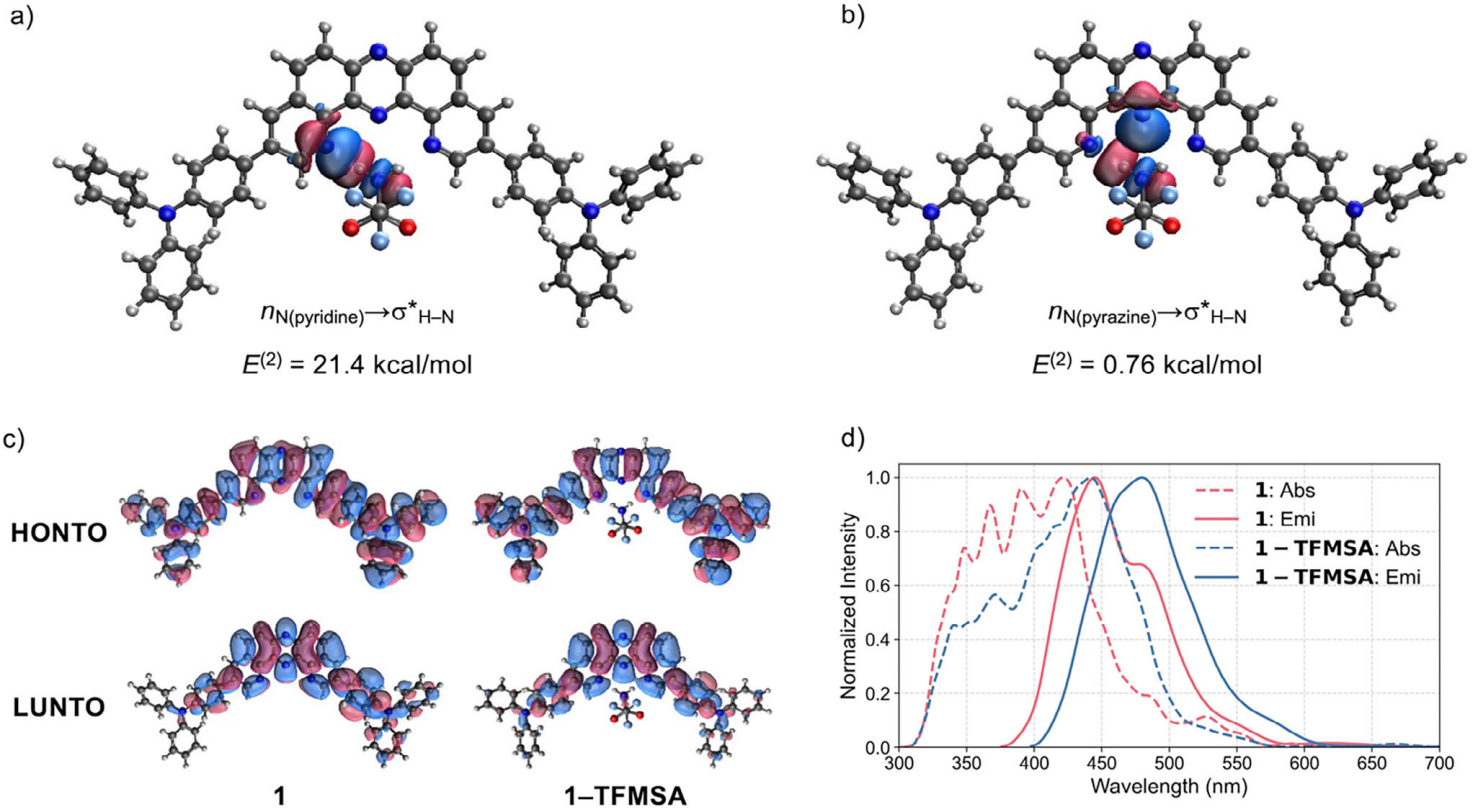
a) NBO overlap interaction surface plot for 1:1 hydrogen‐bonded complex of **1** and TFMSA showing *n*_N (pyridine)_→σ*_H–N_ interaction; b) NBO overlap interaction surface plot for 1:1 hydrogen‐bonded complex of **1** and TFMSA showing *n*_N (pyrazine)_→σ*_H–N_ interaction; c) NTOs of **1** and **1–TFMSA** for S_0_→S_1_ transitions; d) Absorption and emission spectra of **1** and **1–TFMSA** simulated with TD‐DFT and Nuclear Ensemble Method.

To gain deeper insights into the hydrogen bonding interactions between **1** and TFMSA, and to understand how complexation modulates the electronic structure in both ground and excited states, quantum chemical calculations were performed (Figure 6, see Supporting Information for details). Optimization of the equilibrium geometries confirms that TFMSA binds in the same conformation in both the ground and excited state. Complexation leads to the energetic stabilization of the LUMO, which has a delocalization tail at the TFMSA molecule (Table S5 and Figure S41). The hydrogen bonding energy between **1** and TFMSA was estimated to −23.9 kcal/mol in the ground state and −37.1 kcal/mol in the first singlet excited state. The excited‐state value was calculated as the energy difference between the fully optimized complex in the excited state and the sum of individually optimized **1** in the excited state and TFMSA in the ground state. This substantial increase in binding energy upon excitation reflects a strengthening of the hydrogen bond, likely due to geometric relaxation driven by a partial delocalization of the exciton to the hydrogen donor. This interpretation is further corroborated by the analysis of vibrational frequencies. The frequency of the normal mode responsible for stretching of the N─H bonds in TFMSA changes from 3270 cm^−1^ in the ground state to 3066 cm^−1^ in the excited state. Such noticeably lower frequency indicates stronger interaction between the hydrogens in TFMSA and the nitrogen atoms in **1**.

Natural bond orbital (NBO) analysis was conducted to elucidate the nature of these interactions (Figure 6a, b) [19]. The second‐order perturbation energy, *E* ^(2)^, for the interaction between the lone pair on the pyridine‐like sp^2^ nitrogen atom in the inner cleft and the N─H anti‐bonding orbital *n*_N(pyridine)_→σ*_H─N_ was calculated to be 21.4 kcal/mol (Figure 6a), indicating that the hydrogen bonding in the complex exhibits both electrostatic and charge transfer interaction characteristics. Additionally, a weak interaction was observed between the lone pair on the central pyrazine nitrogen atom and the N─H antibonding orbital, with a significantly lower *E* ^(2)^ value of 0.76 kcal/mol. These findings indicate that the DPyPHZ core primarily functions as a two‐point bifurcated hydrogen bonding receptor. Furthermore, the presence of the pyrazine ring in the DPyPHZ framework effectively lowers the LUMO energy level, thereby enhancing the electron density contrast between the donor and acceptor units, which is crucial for distinct charge‐transfer behavior.

The properties of the excited states were simulated using time‐dependent functional theory calculations. The natural transition orbitals (NTOs) for the S_0_→S_1_ transition in **1** and its complex with TFMSA (**1–TFMSA)** are illustrated in Figure 6c. For both systems, the transitions are qualitatively similar mixtures of a charge transfer and locally excited character. However, in the complex, a partial delocalization of the excited electron to TFMSA is noticeable. This delocalization explains the experimentally observed redshift in the absorption spectra (Figure 4a). Indeed, simulations of the absorption spectra using the nuclear ensemble approach [20, 21] (Figure 6d) reveal that the intensities of the higher‐energy bands of **1** decrease while a new red‐shifted peak emerges upon complexation. The positions of the first absorption peaks agree very well with the experiment, and the simulated redshift agrees quantitatively. A similar redshift is present in the simulated emission spectra, also fully in line with the experiment. However, the Stokes shifts are underestimated by ∼0.3 eV, which indicates some additional stabilization of the excited state not captured in simulations, either through structural relaxation or solvent effects. We also note that the redshift of the band edges is not well reproduced, likely due to the limitations of the computational method at large vibrational displacements. The increase in CT character upon complexation was also corroborated by the larger electronic dipole moments of **1‐TFMSA** [8.21 D(S_0_)/27.86 D (S_1_)] compared with those of **1** [0.77 D (S_0_)/1.99 D (S_1_)].

## 3 Conclusion

In summary, we have developed a donor–π–acceptor–π–donor organic fluorophore that exhibits pronounced red‐shifts in both absorption and photoluminescence spectra upon forming a hydrogen‐bonded complex with V‐shaped neutral hydrogen bond donors. Remarkably, this supramolecular modulation remains operative even in dispersed solid‐state environments. The formation of geometrically complementary *n*→σ* (H─E, E = O, N) interactions reinforces hydrogen bonding in the ground state, resulting in cooperative stabilization of the complex. In the excited state, these interactions suppress dissociation or dynamic fluctuations of the hydrogen bond, thereby stabilizing the charge transfer excited state. Furthermore, the structural rigidification induced by strong bifurcated hydrogen bonding effectively suppresses nonradiative decay pathways, leading to enhanced photoluminescence quantum yields through restriction of intramolecular motions. This work offers a new molecular design strategy for modulating the photophysical behavior of π‐conjugated systems via direct electronic perturbation through hydrogen bonding. Our findings deepen the understanding of hydrogen‐bonding effects in π‐conjugated systems. Although this study focuses on fundamental aspects of the hydrogen‐bonded complex, the present findings may provide a useful starting point for future development of functional materials: for example, in water‐responsive or humidity‐sensing contexts [22], as well as in designing optoelectronic systems or probes whose emission properties can be modulated by hydrogen‐bonding environment.

## Conflicts of Interest

The authors declare no conflict of interest.

## Supporting information



The authors have cited additional references within the Supporting Information [23, 24, 25, 26, 27, 28, 29, 30, 31, 32].
**Supporting File 1**: chem70632‐sup‐0001‐SuppMat.pdf.

**Supporting File 2**: chem70632‐sup‐0002‐Data.zip.
