# Supplementary material for: Dual‐State Photophysical Modulation via Bifurcated Hydrogen Bonding in a U‐Shaped Dipyridophenazine‐Cored Donor‐π‐Acceptor‐π‐Donor Fluorophore
Source: Chemistry. 2025 Dec 26;32(7):e03421. doi: 10.1002/chem.202503421 (PMC12910415; doi:10.1002/chem.202503421)
Supplement: Supplementary file 1 — The authors have cited additional references within the Supporting Information [23, 24, 25, 26, 27, 28, 29, 30, 31, 32]. Supporting File 1: chem70632‐sup‐0001‐SuppMat.pdf. [file CHEM-32-e03421-s001.pdf]

Table of Content

**General Remarks**	S1
**Synthetic Procedures and Spectroscopic Data of New Compounds**	S2–S13
**Single Crystal X-Ray Crystallographic Analysis (Table S1)**	S14–S15
**Steady-State Photophysical Properties of 1–3 in Solution (Figure S1–S7, Table S2–S4)**	S16–S21
**Cyclic Voltammetry (Figure S8)**	S22
**Job Plot (Figure S9)**	S22–S23
**Photophysical properties of 1 with addition of TFMSA (Figure S10–11)**	S23–S24
**UV-Vis Absorption and PL Titration Experiments (Figure S12–S15)**	S24–S28
**Monitoring of ^{1}H NMR of 1 and TFMSA (Figure S16)**	S28–S29
**^{1}H NMR Titration of 1 with Water (Figure S17)**	S29–S30
**Preparation of Emitter-Doped Films (Figure S18–S20)**	S30–S32
**Femtosecond-to-Nanosecond Transient Absorption Spectroscopy and Microsecond Transient Absorption Spectroscopy (Figure S21–S40)**	S33–S41
**Theoretical Calculations (Figure S41, Table S5–S6)**	S42–S44
**Copies of NMR Charts of New Compounds**	S45–S59
**References**	S60–S61

General Remarks. All reactions were carried out under an atmosphere of nitrogen unless otherwise noted. Products were purified by chromatography on silica gel Chromatorex BW-300 and NH-DM1020 (Fuji Silysia Chemical Ltd.). Analytical thin-layer chromatography (TLC) was performed on pre-coated silica gel glass plates (Merck silica gel 60 F_{254} TLC plate and Fuji Silysia Chromatorex NH, 0.25 mm thickness). Compounds were visualized with UV lamp. Melting points were determined on a Stanford Research Systems MPA100 OptiMelt Automated Melting Point System. All ^{1}H and ^{13}C NMR were recorded on a JEOL JMTC-400/54/SS Spectrometer (^{1}H NMR, 400 MHz; ^{13}C NMR, 100 MHz) using tetramethylsilane as an internal standard. Infrared spectra were acquired on a SHIMADZU IRAffinity-1 FT-IR Spectrometer. Mass spectra and High-resolution mass spectra were obtained on a JEOL JMS-700 Mass Spectrometer. The elemental analysis (CHN) was carried out with JM10 (J-SCIENCE LAB CO., Ltd). UV-Vis spectra were recorded on a Shimadzu UV-2550 spectrophotometer. Steady-state emission spectra were recorded on a JASCO FP-8650 NIR spectrofluorometer, and absolute photoluminescence quantum yields were calculated with a HAMAMATSU Quantaurus-QY C11347-01 spectrometer with an integrating sphere. Lifetime measurement was conducted with a time-correlated single-photon counting (TCSPC) system HAMAMATSU Quantaurus-Tau C11367. Cyclic voltammetry (CV) was performed with ALS-610E (BAS Inc.) system.

Materials. Dehydrated toluene and THF used for organic synthesis were purified by passing through a solvent purification system. 3,11-dibromo-dibenzo[*a,j*]phenazine (**S9**) [CAS No. 1620543-64-7][S1] was synthesized according to the reported procedure. Other reagents were purchased and used as received. Solvents of fluorescence spectroscopic grade for measurement of UV-Vis and emission spectra were purchased from Nacalai Tesque Inc.

Theoretical Calculations. Molecular geometry optimizations and frequency calculations were performed with (time-dependent) density functional theory (DFT) at the PBE0/6-31+G(d,p) level. Photophysical properties were investigated using nuclear ensemble method as implemented in the NEMO software.[S2] It involves using time-dependent density functional theory (TD-DFT) with the long-range corrected hybrid functional LRC-ωPBEh, employing a tuned range-separation parameter[S3] (ω = 0.124 bohr^{-1}) along with the 6-31+G(d,p) basis set in toluene solvent. Ensembles were generated from S_0 and S_1 states, each with 500 conformations, both for compounds 1 and its complex with TFMSA. Natural bond orbitals (NBO) and natural transition orbitals (NTO) were also calculated at the same level of theory and visualized using IQmol software. Gaussian 16[S4] and QChem 5.0[S5] software were employed in these calculations.

Synthetic Procedures and Spectroscopic Data of New Compounds

Compound **8** was synthesized according to the following chemical equations (S1–S3).

*Synthesis of di-tert-butyl 1,2-di(quinolin-7-yl)hydrazine-1,2-dicarboxylate (**5**)*

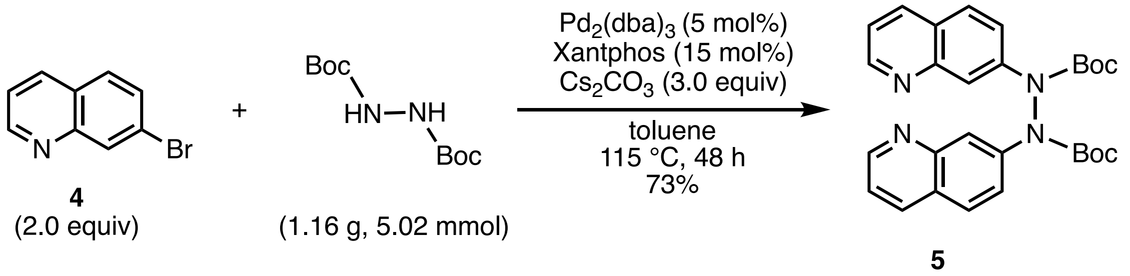

(S1)

The title compound **5** was prepared according to a similar method reported in the literature.[S6] To a two-necked reaction tube (50 mL) equipped with a magnetic stir bar and a rubber septum, were added 7-bromoquinoline (**4**) (2.08 g, 10.0 mmol, 2.0 equiv), di-*tert*-butyl hydrazine-1,2-dicarboxylate (1.16 g, 5.02 mmol, 1.0 equiv), $Pd_2(dba)_3$ (229.4 mg, 0.251 mmol, 5 mol%), Xantophos (441.6 mg, 0.763 mmol, 15 mol%), Cs_2CO_3 (4.90 g, 15.0 mmol, 3.0 equiv). The tube was sealed with another rubber septum, evacuated, and purged with nitrogen gas for three cycles. Toluene (10 mL) was added to the tube through a septum with a syringe. The resulting mixture was stirred in a Personal Organic Synthesizer (EYELA, *Chemi-Station*) equipped with a cooling system under a reflux condition (aluminum block temperature: 115 °C) for 48 h. The reaction mixture was allowed to cool down to room temperature. EtOAc (30 mL) was added, and the mixture was filtered through a celite pad. Solvents in the filtrate were evaporated under reduced pressure to give viscous brown crude solid (3.22 g). The solid residue was purified by flash column chromatography on NH silica gel (eluent: *n*-hexane:EtOAc = 8:2 to 7:3). The collected fractions were combined, and the solvents were evaporated under reduced pressure to give the title compound **5** as yellow solid in 73% yield (1.77 g, 3.65 mmol). Mp 155.0 °C; R_f 0.53 (*n*-hexane/EtOAc 2:8, NH silica); ^{1}H NMR (400 MHz, DMSO-d_6) δ 8.86 (dd, J = 4.0, 1.6 Hz, 2H), 8.33–8.31 (m, 2H), 7.98 (d, J = 8.8 Hz, 2H), 7.92 (d, J = 1.6 Hz, 2H), 7.74 (d, J = 8.4 Hz, 2H), 7.48 (dd, J = 8.0, 4.4 Hz, 2H), 1.53 (s, 18H); ^{13}C NMR (100 MHz, DMSO-d_6) δ 152.2, 151.3, 147.7, 141.1, 135.7, 128.6, 125.5, 121.8 (br), 121.3, 119.4 (br), 82.9, 27.7; IR (ATR, cm^{-1}): 2976, 1721, 1622, 1501, 1368, 1321, 1294, 1252, 1146, 1121, 1057, 1020, 833, 770, 754; MS (EI): *m/z* (relative intensity, %) 487 ($[M+1]^+$, 4), 486 (M^+, 13), 386 ($[M–Boc+1]^+$, 19), 285 ($[M–2Boc+1]^+$, 100); HRMS (MALDI-TOF, positive, CHCA matrix): *m/z* calcd for $C_{28}H_{31}N_4O_4$ $[M+H]^+$ 487.2345, found 487.2340.

Synthesis of [8,8'-biquinoline]-7,7'-diamine (7)

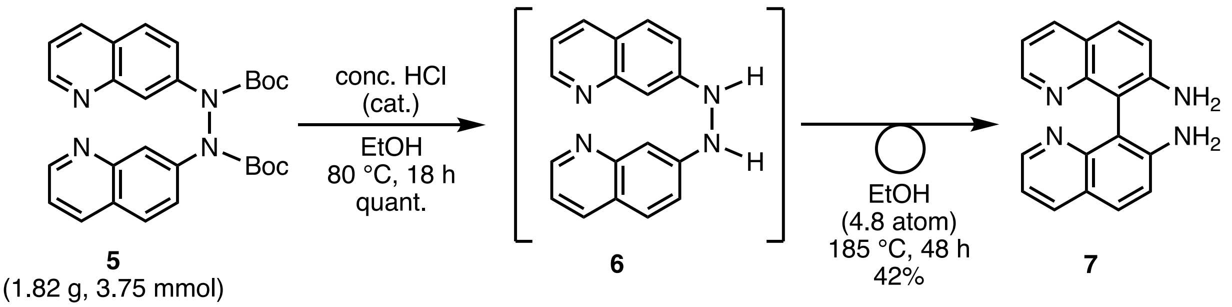

(S2)

To a three-necked flask (300 mL) equipped with a magnetic stirring bar, were added di-*tert*-butyl 1,2-di(quinolin-7-yl)hydrazine-1,2-dicarboxylate (**5**) (1.82 g, 3.75 mmol), EtOH (60 mL), conc. HCl (2.4 mL). The flask was equipped with an air-cooled condenser tube (Asynt, *CondenSyn*) and sealed with a septum and a glass stopper. The resulting mixture was stirred in an aluminum heating block under a reflux condition (aluminum block temperature: 80 °C) for 18 h. The reaction mixture was allowed to cool down to room temperature and neutralized with aq. $NaHCO_3$. The organic layer was extracted with EtOAc (30 mL×3), and the combined organic extract was dried over Na_2SO_4 and filtered. Solvents in the filtrate were evaporated under reduced pressure to give yellow crude solid containing 1,2-di(quinolin-7-yl)hydrazine (**6**) (1.14 g, quant). The quantitative formation of **6** was confirmed with ^{1}H NMR (*note*: compound **6** is easily oxidized to corresponding azo compound under air and during purification with silica gel. Therefore, without further purification, the crude product was employed in the following reaction). Mp 223.0 °C (dec.); R_f 0.12 (*n*-hexane/EtOAc 2:8, NH silica); ^{1}H NMR (400 MHz,) δ 8.73 (dd, J = 4.4, 1.0 Hz, 2H), 8.00 (dd, J = 8.4, 1.6 Hz, 2H), 7.67 (d, J = 8.8 Hz, 2H), 7.41 (d, J = 2.0 Hz, 2H), 7.20–7.16 (m, 4H), 6.18 (br, 2H); IR (ATR, cm^{-1}): ν 3208, 2994, 2959, 1616, 1437, 1352, 1314, 1261, 1140, 935, 822, 760; MS (EI$^+$): *m/z* (relative intensity, %) 287 ([M+1]$^+$, 22) , 286 (M$^+$, 100), 270 ([M–NH_2]$^+$, 59), 156 ([$C_9H_6N_3$]$^+$, 10), 128 ([C_9H_6N]$^+$, 10); HRMS (EI$^+$): *m/z* calcd for $C_{18}H_{14}N_4$ (M) 286.1218, found 286.1217.

To an autoclave (100 mL), were added the crude product (1.14 g) and EtOH (15 mL), which was purged with nitrogen gas. The autoclave was sealed, and the reaction mixture was stirred in an aluminum heating block (aluminum block temperature: 185 °C; pressure: 4.8 atm) for 48 h. The reaction mixture was allowed to cool down to room temperature, and solvents were removed from the mixture under reduced pressure to give brown crude solid (1.13 g). The solid residue was purified by flash column chromatography on NH silica gel (eluent: *n*-hexane/EtOAc = 4:6 to 0:10) to give the title compound **7** as yellow solid (455.6 mg, 1.58 mmol, 42% in 2 steps). Mp 269.0 °C (dec.); R_f 0.25 (EtOAc, NH silica); ^{1}H NMR (400 MHz, $CDCl_3$) δ 8.68 (dd, J = 4.4, 1.6 Hz, 2H), 8.04 (dd, J = 8.0, 1.6 Hz, 2H), 7.75 (d, J = 8.8 Hz, 2H), 7.21 (d, J = 8.8 Hz, 2H), 7.12 (dd, J = 8.0, 4.4 Hz, 2H), 3.86 (br,

4H); ^{13}C NMR (100 MHz, $CDCl_3$) δ 150.9, 148.3, 146.1, 136.1, 129.0, 123.0, 119.1, 117.6, 114.6; IR (ATR, cm^{-1}): 3455, 3412, 3292, 3169, 3049, 1608, 1593, 1560, 1500, 1427, 1373, 1321, 1285, 1265, 1209, 1138, 1049, 939, 862, 825, 802, 763; MS (EI^+): *m/z* (relative intensity, %) 287 ($[M+1]^+$, 1), 286 (M^+, 5), 270 ($[M–NH_2]^+$, 100) , 143 ($[C_9H_7N_2]^+$, 3); HRMS (EI^+): *m/z* calcd for $C_{18}H_{14}N_4$ (M) 282.1218, found 282.1210.

*Synthesis of dipyrido[2,3-a:3',2'-j]phenazine (**8**) [CAS No. 10088-28-5]*

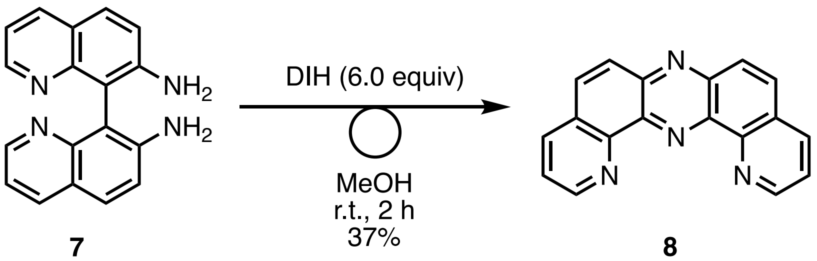

7
(138 mg, 0.48 mmol)

8

(S3)

To a three-necked flask (100 mL) equipped with a magnetic stirring bar, were added [8,8'-biquinoline]-7,7'-diamine (**7**) (138 mg, 0.48 mmol) and MeOH (50 mL). The resulting mixture was stirred at room temperature until compound **7** was completely dissolved. The flask was purged with nitrogen gas and sealed with two rubber septa and a glass stopper. To the solution, was added 1,3-diiode-5,5-dimethylhydantoin (DIH) (1.10 g, 2.90 mmol, 6.0 equiv), and the resulting mixture was stirred at room temperature for 2 h. Aqueous solution of sodium thiosulfate (1 M, 50 mL) was added to the reaction mixture, and organic layer was extracted with $CHCl_3$ (30 mL×3). The combined organic extract was dried over Na_2SO_4 and filtered. Solvents in the filtrate were evaporated under reduced pressure to give viscous brown crude solid (212 mg), which was purified by flash column chromatography on NH silica gel (eluent: *n*-hexane:$CHCl_3$ = 5:5 to 0:10). The collected fractions were combined, and the solvents were evaporated under reduced pressure to give the title compound **8** as yellow solid in 37% yield (50.8 mg, 0.18 mmol). Mp 285.5 °C (dec.); R_f 0.05 ($CHCl_3$, NH silica); ^{1}H NMR (400 MHz, $CDCl_3$) δ 9.35 (dd, J = 4.4, 1.2 Hz, 2H), 8.37 (dd, J = 8.0, 1.2 Hz, 2H), 8.25 (d, J = 9.2 Hz, 2H), 8.16 (d, J = 9.2 Hz, 2H), 7.79 (dd, J = 8.4, 4.4 Hz, 2H); ^{13}C NMR (100 MHz, $CDCl_3$) δ 150.6, 146.3, 144.9, 140.9, 136.4, 131.7, 128.8, 128.1, 124.2; IR (ATR, cm^{-1}): 3248, 3017, 1690, 1606, 1593, 1541, 1508, 1470, 1422, 1362, 1109, 850, 831, 738; MS (EI^+): *m/z* (relative intensity, %) 283 ($[M+1]^+$, 23), 282 (M^+, 100), 256 ($[M–26]^+$, 7), 127 ($[C_9H_5N]^+$, 3); HRMS (EI^+): *m/z* calcd for $C_{18}H_{10}N_4$ (M) 282.0905, found 282.0898.

Compound **1** was synthesized according to the following chemical equations (S4–S8).

*Synthesis of 7-bromo-3-chloroquinoline (**S1**) [CAS No. 1246549-62-1]*[S3]

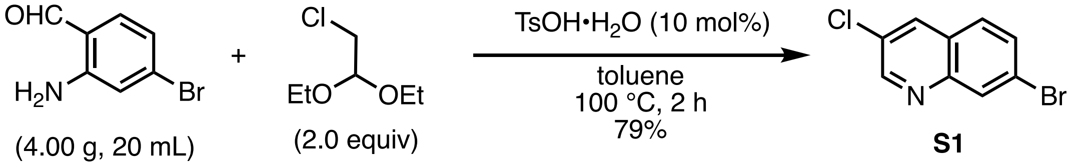

(S4)

The title compound was synthesized according to a procedure reported in literature.[S7] To a three-necked flask (300 mL) equipped with a magnetic stir bar, were added toluene (120 mL), TsOH•H_2O (380 mg, 2.0 mmol, 10 mol%), 2-amino-4-bromobenzaldehyde (4.00 g, 20 mmol), and 2-chloro-1,1-diethoxyethane (6.10 mL, 40 mmol, 2.0 equiv). The flask was equipped with an air-cooled condenser tube (Asynt, *CondenSyn*), purged with nitrogen gas, and sealed with a septum and a glass stopper. The reaction mixture was stirred in an aluminum heating block (aluminum block temperature: 100 °C) for 2 h. The reaction mixture was allowed to cool down to room temperature and neutralized with aq. $NaHCO_3$. The organic layer was extracted with EtOAc (20 mL×3), and the combined organic extract was dried over Na_2SO_4 and filtered. Solvents in the filtrate were evaporated under reduced pressure to give brown crude solid (5.60 g). The solid residue was purified by flash column chromatography on NH silica gel (eluent: *n*-hexane:EtOAc = 95:5). The collected fractions were combined, and the solvents were evaporated under reduced pressure to give the title compound **7** as pale yellow solid in 79% yield (3.82 g, 15.7 mmol). Mp 91.2 °C; R_f 0.63 (*n*-hexane/EtOAc 2:8, NH silica); ^{1}H NMR (400 MHz, DMSO-d_6) δ 8.94 (d, J = 2.4 Hz, 1H), 8.66 (d, J = 2.4 Hz, 1H), 8.30 (s, 1H), 7.98 (d, J = 8.8 Hz, 1H), 7.86 (dd, J = 8.8, 2.0 Hz, 1H); ^{13}C NMR (100 MHz, $CDCl_3$) δ 150.6, 146.7, 133.9, 131.9, 131.3, 128.8, 128.2, 127.0, 123.7; IR (ATR, cm^{-1}): 3036, 1584, 1551, 1476, 1429, 1362, 1321, 1260, 1244, 1159, 1142, 1086, 1061, 951, 897, 878, 808, 773; MS (EI$^+$): *m/z* (relative intensity, %) 245 ($[M+4]^+$,1), 243 ($[M+2]^+$, 40), 241 (M^+, 34), 162 ($[M–Br]^+$, 11), 136 ($[M–Br–26]^+$, 49); HRMS (EI$^+$): *m/z* calcd for C_9H_5BrClN (M) 240.9294, found 240.9297. These spectroscopic data were in good agreement with that previously reported in literature.[S7]

*Synthesis of di-tert-butyl 1,2-bis(3-chloroquinolin-7-yl)hydrazine-1,2-dicarboxylate (**S2**)*

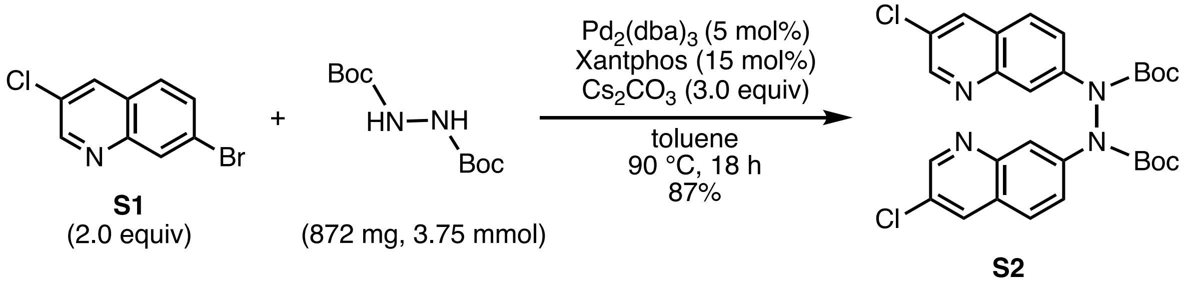

(S5)

The title compound **S2** was prepared according to a similar method reported in the literature.[S6] To a two-necked reaction tube (50 mL) equipped with a magnetic stir bar and a rubber septum, were added 7-bromo-3-quinoline (**S1**) (1.82 g, 7.5 mmol, 2.0 equiv), di-*tert*-butyl hydrazine-1,2-dicarboxylate (872 mg, 3.75 mmol, 1.0 equiv), $Pd_2(dba)_3$ (174.0 mg, 0.19 mmol, 5 mol%), Xantophos (328.0 mg, 0.56 mmol, 15 mol%), Cs_2CO_3 (3.67 g, 11.0 mmol, 3.0 equiv). The tube was sealed with another rubber septum, evacuated, and purged with nitrogen gas for three cycles. Toluene (15 mL) was added to the tube through a septum with a syringe. The resulting mixture was stirred in a Personal Organic Synthesizer (EYELA, *Chemi-Station*) equipped with a cooling system (aluminum block temperature: 90 °C) for 18 h. The reaction mixture was allowed to cool down to room temperature. EtOAc (20 mL) was added, and the mixture was filtered through a celite pad. Solvents in the filtrate were evaporated under reduced pressure to give viscous brown crude solid (1.87 g). The solid residue was purified by flash column chromatography on NH silica gel (eluent: *n*-hexane:EtOAc = 9:1 to 8:2). The collected fractions were combined, and the solvents were evaporated under reduced pressure to give the title compound **5** as yellow solid in 87% yield (1.82 g, 3.30 mmol). Mp 155.0 °C; R_f 0.28 (*n*-hexane/EtOAc 8:2, NH silica); ^{1}H NMR (400 MHz, DMSO-d_6) δ 8.86 (d, J = 2.4 Hz, 2H), 8.55 (d, J = 2.4 Hz, 2H), 7.99–7.96 (m, 4H), 7.83 (br, 2H), 1.52 (s, 18H); ^{13}C NMR (100 MHz, DMSO-d_6) δ 152.0, 150.1, 145.8, 141.4, 133.9, 128.1, 127.3, 125.7, 123.1 (br), 119.2 (br), 83.3, 27.7; IR (ATR, cm^{-1}): 2978, 1717, 1618, 1491, 1368, 1306, 1285, 1250, 1157, 1140, 1086, 1061, 905, 883, 768, 758; MS (EI): *m/z* (relative intensity, %) 556 ([M+2]$^+$, 3), 554 (M$^+$, 4), 458 ([M–Boc+4]$^+$, 1), 456 ([M–Boc+2]$^+$, 4), 454 ([M–Boc]$^+$, 6), 356 ([M–2Boc+4]$^+$, 24), 354 ([M–2Boc+2]$^+$, 42), 352 ([M–2Boc]$^+$, 3), 162 ([C_9H_5ClN]$^+$, 22); HRMS (EI$^+$): *m/z* calcd for $C_{28}H_{28}Cl_2N_4O_4$ (M) 554.1488, found 554.1495.

*Synthesis of 3,3'-dichloro-[8,8'-biquinoline]-7,7'-diamine (**S4**)*

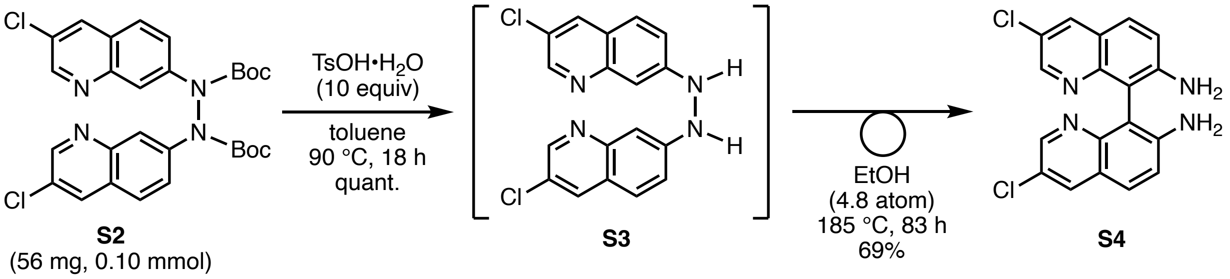

(S6)

To a two-necked flask (100 mL) equipped with a magnetic stirring bar, were added di-*tert*-butyl 1,2-di(quinolin-7-yl)hydrazine-1,2-dicarboxylate (**S2**) (56.0 mg, 0.101 mmol) and TsOH•H_2O (198 mg, 1.04 mmol, 10 equiv). The flask was equipped with an air-cooled condenser tube (Asynt, *CondenSyn*), purged with nitrogen gas for three times, and sealed with a rubber septum and a glass stopper. To the flask, EtOH (10 mL) was injected through septum. The resulting mixture was stirred

in an aluminum heating block under a reflux condition (aluminum block temperature: 85 °C) for 8 h. The reaction mixture was allowed to cool down to room temperature and neutralized with aq. $NaHCO_3$. The organic layer was extracted with EtOAc (30 mL×3), and the combined organic extract was dried over Na_2SO_4 and filtered. Solvents in the filtrate were evaporated under reduced pressure to give yellow crude solid containing 1,2-bis(3-chloroquinolin-7-yl)hydrazine (**S3**) (35.6 mg, quant). The quantitative formation of **S3** was confirmed with ^{1}H NMR (*note*: compound **S3** is easily oxidized to corresponding azo compound under air and during purification with silica gel. Therefore, without further purification, the crude product was employed in the following reaction). Mp 202.5 °C (dec.); R_f 0.28 (EtOAc, NH silica); ^{1}H NMR (400 MHz, $CDCl_3$) δ 8.65 (d, J = 2.8 Hz, 2H), 7.99 (d, J = 2.4 Hz, 2H), 7.63 (d, J = 8.8 Hz, 2H), 7.38 (d, J = 2.0 Hz, 2H), 7.21 (dd, J = 8.8, 2.4 Hz, 2H), 6.19 (br, 2H); IR (ATR, cm^{-1}): 3240, 3042, 2988, 1639, 1620, 1520, 1422, 1350, 1319, 1204, 1088, 949, 895, 806, 768, 748; MS (EI^+, direct): *m/z* (relative intensity, %) 356 ($[M+2]^+$, 6), 355 ($[M+1]^+$, 6), 354 (M^+, 11), 342 ($[M–NH_2+4]^+$, 13), 340 ($[M–NH_2+2]^+$, 68), 338 ($[M–NH_2]^+$, 100), 303($[M–NH_2–Cl]^+$, 10), 177 ($[C_9H_6ClN_2]^+$, 3), 162 ($[C_9H_5ClN]^+$, 9); HRMS (EI^+, direct): *m/z* calcd for $C_{18}H_{12}Cl_2N_4$ (M) 354.0439, found 354.0440.

To an autoclave (100 mL), were added the crude product (373.3 mg) and EtOH (60 mL), which was purged with nitrogen gas. The autoclave was sealed, and the reaction mixture was stirred in an aluminum heating block (aluminum block temperature: 185 °C; pressure: 4.8 atm) for 83 h. The reaction mixture was allowed to cool down to room temperature, and solvents were removed from the mixture under reduced pressure to give brown crude solid (357.7 mg). The solid residue was purified by flash column chromatography on NH silica gel (eluent: *n*-hexane/EtOAc = 7:3 to 6:4) to give the title compound **S4** as yellow solid (245.3 mg, 0.69 mmol, 69% in 2 steps). Mp 177.1 °C; R_f 0.40 (EtOAc, NH silica); ^{1}H NMR (400 MHz, DMSO-d_6) δ 8.34 (d, J = 2.0 Hz, 2H), 8.23 (d, J = 2.4 Hz, 2H), 7.71 (d, J = 8.8 Hz, 2H), 7.26 (d, J = 9.2 Hz, 2H), 5.05 (br, 4H); ^{13}C NMR (100 MHz, DMSO-d_6) δ 148.0, 147.7, 147.0, 133.6, 127.4, 122.2, 121.8, 120.4, 112.2; IR (ATR, cm^{-1}): 3447, 3352, 3059, 1606, 1487, 1418, 1402, 1362, 1337, 1323, 1169, 1150, 1098, 943, 893, 792; MS (EI^+, direct): *m/z* (relative intensity, %) 358 ($[M+4]^+$, 1), 356 ($[M+2]^+$, 4), 354 (M^+, 7), 342 ($[M–NH_2+4]^+$, 12), 340 ($[M–NH_2+2]^+$, 71), 338 ($[M–NH_2]^+$, 100), 305 ($[M–NH_2–Cl+2]^+$, 2), 303 ($[M–NH_2–Cl]^+$, 7), 177 ($[C_9H_6ClN_2]^+$, 2); HRMS (EI^+, direct): *m/z* calcd for $C_{18}H_{12}Cl_2N_4$ (M) 354.0439, found 354.0434.

*Synthesis of 3,11-dichlorodipyrido[2,3-a:3',2'-j]phenazine (**9**)*

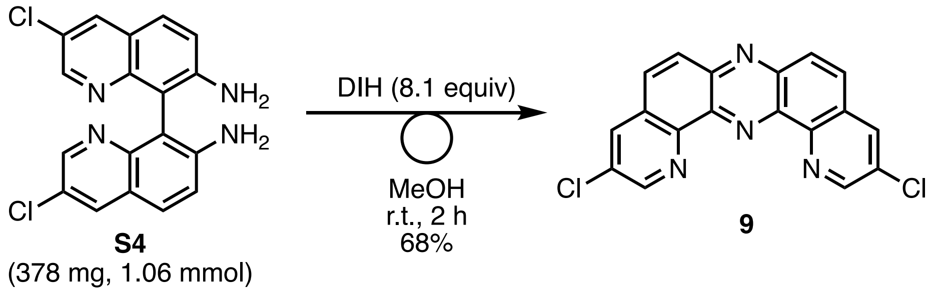

(S7)

To a three-necked flask (200 mL) equipped with a magnetic stirring bar, were added 3,3'-dichloro-[8,8'-biquinoline]-7,7'-diamine (**S4**) (378.3 mg, 1.06 mmol) and MeOH (100 mL). The resulting mixture was stirred at room temperature until compound **S4** was completely dissolved. The flask was purged with nitrogen gas and sealed with two rubber septa and a glass stopper. To the solution, was added 1,3-diiode-5,5-dimethylhydantoin (DIH) (3.07 g, 8.08 mmol, 8.1 equiv), and the resulting mixture was stirred at room temperature for 2 h. Aqueous solution of sodium thiosulfate (1 M, 100 mL) was added to the reaction mixture, and organic layer was extracted with $CHCl_3$ (30 mL×3). The combined organic extract was dried over Na_2SO_4 and filtered. Solvents in the filtrate were evaporated under reduced pressure to give viscous brown crude solid (530.3 mg), which was purified by flash column chromatography on NH silica gel (eluent: *n*-hexane:EtOAc = 8:2 to 0:10). The collected fractions were combined, and the solvents were evaporated under reduced pressure to give the title compound **9** as yellow solid in 68% yield (253.0 mg, 0.72 mmol). Further purification was conducted with recrystallization from $CHCl_3$. Mp 361.7 ℃ (dec.); R_f 0.11 (EtOAc, NH silica); ^{1}H NMR (400 MHz, $CDCl_3$) δ 9.25 (d, J = 2.4 Hz, 2H), 8.33 (d, J = 2.0 Hz, 2H), 8.27 (d, J = 9.2 Hz, 2H), 8.09 (d, J = 9.2 Hz, 2H); ^{13}C NMR (100 MHz, $CDCl_3$) δ 150.1, 144.8, 144.2, 141.0, 134.6, 132.5, 130.8, 129.5, 129.4; IR (ATR, cm^{-1}): 3063, 3045, 3032, 1584, 1495, 1422, 1362, 1296, 1207, 1107, 1015, 922, 889, 845, 824, 810, 735, 708; MS (EI$^+$, direct): *m/z* (relative intensity, %) 354 ([M+4]$^+$, 12), 352 ([M+2]$^+$, 69), 350 (M$^+$, 100), 317 ([M–Cl+2]$^+$, 10), 315([M–Cl]$^+$, 27), 177 ([$C_9H_4ClN_2$]$^+$, 9); HRMS (EI$^+$, direct): *m/z* calcd for $C_{18}H_8Cl_2N_4$ (M) 350.0126, found 350.0135.

*Synthesis of 4,4'-(dipyrido[2,3-a:3',2'-j]phenazine-3,11-diyl)bis(N,N-diphenylaniline) (**1**)*

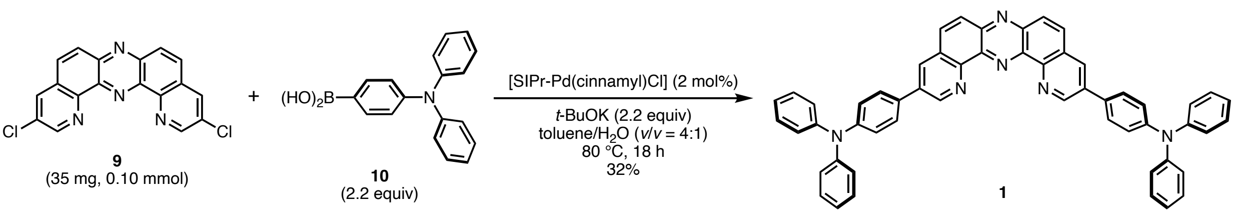

(S8)

To a flame-dried two-necked tube (50 mL) equipped with a magnetic stir bar, were added 3,11-dichlorodipyrido[2,3-*a*:3',2'-*j*]phenazine (**9**) (35.0 mg, 0.10 mmol) and (4-

(diphenylamino)phenyl)boronic acid (**10**) (68.0 mg, 0.22 mmol, 2.2 equiv). The tube was transferred into a glove box, where *t*-BuOK (25.0 mg, 0.22 mmol, 2.2 equiv) and [SIPr-Pd(cinnamyl)Cl] (1.5 mg, 20 μmol, 2 mol%) were added to the tube. The tube was sealed with two septa and taken from the glovebox, and deoxygenated toluene (1.0 mL) through freeze-throw cycles (3 times) and distilled water (0.25 mL) was added to the flask through a septum with a syringe. The mixture was purged with nitrogen gas and stirred in a Personal Organic Synthesizer (EYELA, *Chemi-Station*) equipped with a cooling system (aluminum block temperature: 80 °C) for 18 h. The reaction mixture was allowed to cool down to room temperature. Water (10 mL) was added to the mixture, and the organic layer was extracted with $CHCl_3$ (15 mL×3). The organic extract was dried over Na_2SO_4 and filtered. Solvents in the filtrate were evaporated under reduced pressure to give crude solid. The solid residue was purified by flash column chromatography on NH silica gel (eluent: *n*-hexane:$CHCl_3$ = 9:1 to 5:5). The collected fractions were combined, and the solvents were evaporated under reduced pressure to give an orange solid (37 mg), which was then reprecipitated from $CHCl_3$/*n*-hexane (1:2) to provide the title compound **1** in 32% yield (25.0 mg, 0.032 mmol) as an orange solid. Mp 165.1 °C; R_f 0.70 ($CHCl_3$, NH silica); ^{1}H NMR (400 MHz, CD_2Cl_2) δ 9.55 (d, J = 2.4 Hz, 2H), 8.51 (d, J = 2.4 Hz, 2H), 8.21 (d, J = 2.4 Hz, 4H), 7.77 (d, J = 8.8 Hz, 4H), 7.34 (dd, J = 8.0, 7.6 Hz, 8H), 7.24 (d, J = 8.4 Hz, 4H), 7.19 (dd, J = 8.4, 1.2 Hz, 8H), 7.11 (t, J = 7.2 Hz, 4H); ^{13}C NMR (100 MHz, $CDCl_3$) δ 149.2, 148.7, 147.2, 144.6, 144.5, 141.0, 136.6, 132.7, 131.8, 129.9, 129.5, 128.9, 128.4, 128.2, 125.0, 123.6, 123.2; IR (ATR, cm^{-1}): 3032, 1585, 1558, 1541, 1508, 1489, 1435, 1317, 1271, 741; MS (FAB$^+$, direct): *m/z* (relative intensity, %) 769 ([M+H]$^+$, 43), 768 (M$^+$, 6); HRMS (FAB$^+$, PEG+NaI): *m/z* calcd for $C_{54}H_{37}N_6$ [M+H]$^+$ 769.3080, found 769.3097.

Compound **2** was synthesized according to the following chemical equations (S9–S13).

*Preparation of di-tert-butyl 1,2-bis(3-chloroisoquinolin-7-yl)hydrazine-1,2-dicarboxylate (**S6**)*

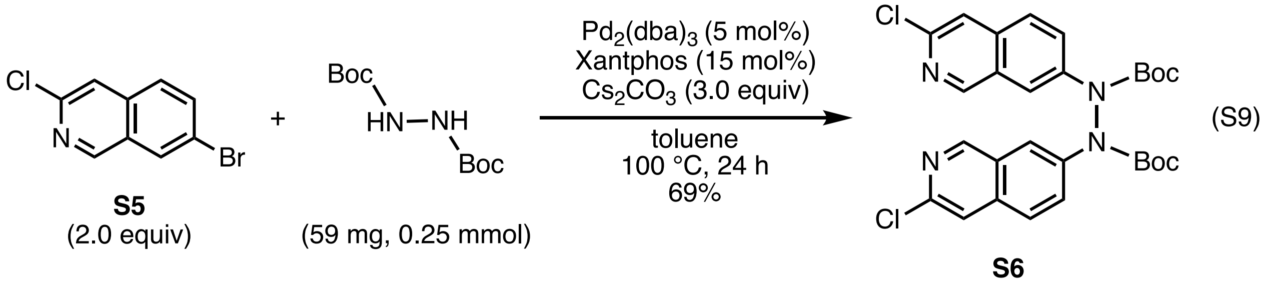

(S9)

The title compound **S6** was prepared according to a similar method reported in the literature.[S6] To a two-necked reaction tube (50 mL) equipped with a magnetic stir bar and a rubber septum, were added 7-bromo-3-chloroisoquinoline (**S5**) (123 mg, 0.50 mmol, 2.0 equiv), di-*tert*-butyl hydrazine-1,2-dicarboxylate (59 mg, 0.25 mmol, 1.0 equiv), $Pd_2(dba)_3$ (12.0 mg, 13 μmol, 5 mol%), Xantophos

(23.0 mg, 38 μmol, 15 mol%), Cs_2CO_3 (244 mg, 0.75 mmol, 3.0 equiv). The tube was sealed with another rubber septum, evacuated, and purged with nitrogen gas for three cycles. Toluene (2 mL) was added to the tube through a septum with a syringe. The resulting mixture was stirred in a Personal Organic Synthesizer (EYELA, *Chemi-Station*) equipped with a cooling system (aluminum block temperature: 100 °C) for 24 h. The reaction mixture was allowed to cool down to room temperature. $CHCl_3$ (10 mL) was added, and the mixture was filtered through a celite pad. To the filtrate, water was added. The organic layer was washed with water, dried over Na_2SO_4, and filtered. Solvents in the filtrate were evaporated under reduced pressure to give viscous orange crude solid (177 mg). The solid residue was purified by flash column chromatography on NH silica gel (eluent: *n*-hexane:EtOAc = 8:2). The collected fractions were combined, and the solvents were evaporated under reduced pressure to give the title compound **S6** as viscous orange solid in 69% yield (97 mg, 0.17 mmol). Mp 165.1 °C; R_f 0.23 (*n*-hexane/EtOAc 8:2, NH silica); ^{1}H NMR (400 MHz, $CDCl_3$) δ 8.99 (s, 2H), 8.03 (br, 2H), 7.84 (br, 2H), 7.74 (d, J = 9.2 Hz, 2H), 7.69 (s, 2H), 1.54 (s, 18H); ^{13}C NMR (100 MHz, $CDCl_3$) δ 152.6, 152.3, 145.7, 140.0, 135.5, 127.5, 126.6, 126.0, 119.31, 119.28, 83.9, 28.1; IR (ATR, cm^{-1}): 2978, 2934, 1719, 1560, 1494, 1393, 1342, 1250, 1143, 1061, 870; MS (FAB$^+$, NBA): *m/z* (relative intensity, %) 559 ($[M+H+4]^+$, 13), 557 ($[M+H+2]^+$, 70), 555 ($[M+H]^+$, 100), 458 ($[M+H–Boc+4]^+$, 4), 456 ($[M+H–Boc+2]^+$, 11), 454 ($[M+H–Boc]^+$, 12), 357 ($[M+H–2Boc+4]^+$, 8), 355 ($[M+H–2Boc+2]^+$, 17), 353 ($[M+H–2Boc]^+$, 17); HRMS (FAB$^+$, NBA: PEG+NaI): *m/z* calcd for $C_{28}H_{29}Cl_2N_4O_4$ $[M+H]^+$ 555.1566, found 555.1579.

*Synthesis of 3,3'-dichloro-[8,8'-biisoquinoline]-7,7'-diamine (**S7**)*

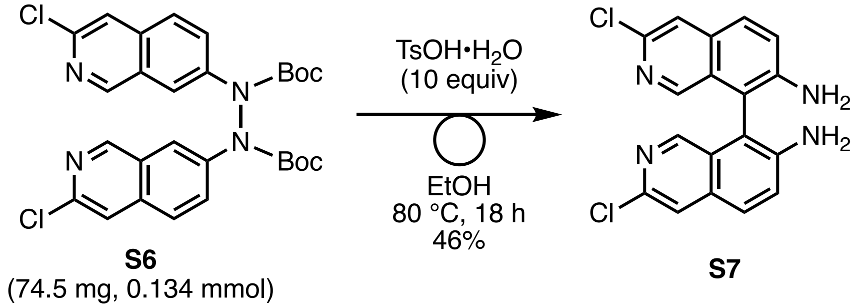

(S10)

To a three-necked flask (200 mL) equipped with a magnetic stirring bar, were added di-*tert*-butyl 1,2-bis(3-chloroisoquinolin-7-yl)hydrazine-1,2-dicarboxylate (**S6**) (74.5 mg, 0.134 mmol) and $TsOH•H_2O$ (436 mg, 1.30 mmol, 10 equiv). The flask was equipped with an air-cooled condenser tube (Asynt, *CondenSyn*), purged with nitrogen gas for three times, and sealed with a rubber septum and a glass stopper. To the flask, EtOH (20 mL) was injected through septum. The resulting mixture was stirred in an aluminum heating block under a reflux condition (aluminum block temperature: 80 °C) for 18 h. The reaction mixture was allowed to cool down to room temperature and neutralized with aq.

$NaHCO_3$. The organic layer was extracted with $CHCl_3$ (20 mL×3), and the combined organic extract was dried over Na_2SO_4 and filtered. Solvents in the filtrate were evaporated under reduced pressure to give yellow crude solid (65.0 mg). The solid residue was purified by flash column chromatography on NH silica gel (eluent: *n*-hexane/EtOAc = 5:5 to 4:6) to give the title compound **S7** as yellow solid (22.0 mg, 62 μmol, 46%). Mp 211.7 °C (dec.); R_f 0.25 ($CHCl_3$, NH silica); ^1H NMR (400 MHz, $CDCl_3$) δ 8.19 (s, 2H), 7.74 (d, J = 8.8 Hz, 2H), 7.66 (s, 2H), 7.35 (d, J = 8.8 Hz, 2H), 3.91 (br, 4H); ^{13}C NMR (100 MHz, $CDCl_3$) δ 148.4, 144.1, 143.1, 133.1, 128.1, 127.4, 123.8, 120.0, 109.0; IR (ATR, cm^{-1}): 3339, 3210, 1616, 1585, 1489, 1339, 1250, 1186, 1070, 930, 864; MS (FAB$^+$, NBA): *m/z* (relative intensity, %) 359 ($[M+H+4]^+$, 3), 357 ($[M+H+2]^+$, 17), 355 ($[M+H]^+$, 26); HRMS (FAB$^+$, direct): *m/z* calcd for $C_{18}H_{13}Cl_2N_4$ $[M+H]^+$ 355.0517, found 355.0507.

*Synthesis of 3,11-dichlorodipyrido[3,4-a:4',3'-j]phenazine (**S8**)*

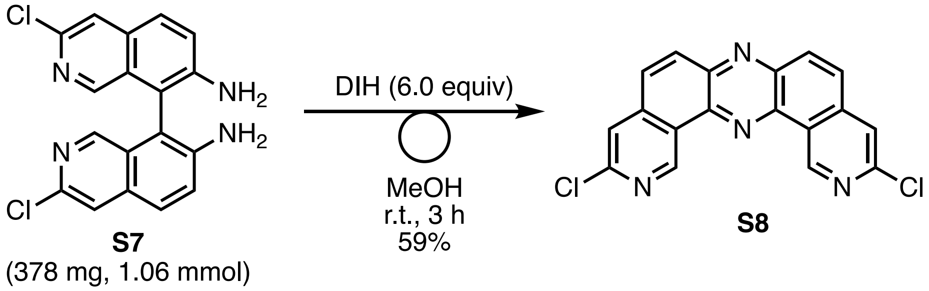

(S11)

To a two-necked flask (100 mL) equipped with a magnetic stirring bar, were added 3,3'-dichloro-[8,8'-biisoquinoline]-7,7'-diamine (**S7**) (72.0 mg, 0.20 mmol) and MeOH (40 mL). The resulting mixture was stirred at room temperature under ultrasonication until compound **S7** was completely dissolved. The flask was purged with nitrogen gas and sealed with a rubber septum and a glass stopper. To the solution, was added 1,3-diiode-5,5-dimethylhydantoin (DIH) (456 mg, 1.20 mmol, 6.0 equiv), and the resulting mixture was stirred at room temperature for 3 h. Aqueous solution of sodium thiosulfate (1 M, 20 mL) was added to the reaction mixture. The precipitates were filtered, washed with $CHCl_3$ (50 mL), and dried under reduced pressure to give the title compound **S8** as yellow solid in 59% yield (41.0 mg, 0.12 mmol). Mp 275.3 °C (dec.); R_f 0.63 ($CHCl_3$, NH silica); The solubility of the compound **S8** is very low in common solvents. NMR spectra were recorded after it was first dissolved in deuterated trifluoroacetic acid (TFA-d_1), then evaporated, and subsequently redissolved in $CDCl_3$. ^1H NMR (400 MHz, $CDCl_3$) δ 10.56 (s, 2H), 8.38 (d, J = 9.2 Hz, 2H), 8.08 (d, J = 9.2 Hz, 2H), 7.97 (s, 2H); ^{13}C NMR (100 MHz, $CDCl_3$) δ 150.4, 148.8, 142.8, 140.7, 140.5, 133.4, 130.4, 124.1, 121.5; IR (ATR, cm^{-1}): 3049, 1558, 1541, 1456, 1333, 1115, 1078, 876, 822; MS (EI$^+$, direct): *m/z* (relative intensity, %) 354 ($[M+4]^+$, 8), 352 ($[M+2]^+$, 66), 350 (M^+, 100), 317 ($[M–Cl+2]^+$, 23),

315 ([M–Cl]$^+$, 67), 280 ([M–2Cl]$^+$, 7), 175 ([$C_9H_4ClN_2$]$^+$, 10); HRMS (EI$^+$, direct): *m/z* calcd for $C_{18}H_8Cl_2N_4$ (M) 350.0126, found 350.0123.

*Synthesis of 4,4'-(dipyrido[3,4-a:4',3'-j]phenazine-3,11-diyl)bis(N,N-diphenylaniline) (**2**)*

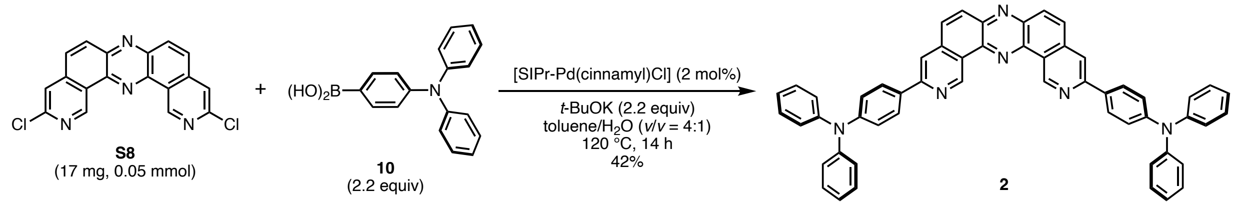

(S12)

To a flame-dried two-necked tube (10 mL) equipped with a magnetic stir bar, were added 3,11-dichlorodipyrido[3,4-*a*:4',3'-*j*]phenazine (**S8**) (17.0 mg, 0.05 mmol) and (4-(diphenylamino)phenyl)boronic acid (**10**) (34.0 mg, 0.11 mmol, 2.2 equiv). The tube was transferred into a glove box, where *t*-BuOK (13.0 mg, 0.11 mmol, 2.2 equiv) and [SIPr-Pd(cinnamyl)Cl] (0.7 mg, 1 µmol, 2 mol%) were added to the tube. The tube was sealed with two septa and taken from the glovebox, and deoxygenated toluene (2.0 mL) through freeze-throw cycles (3 times) and distilled water (0.25 mL) was added to the flask through a septum with a syringe. The mixture was purged with nitrogen gas and stirred in a Personal Organic Synthesizer (EYELA, *Chemi-Station*) equipped with a cooling system (aluminum block temperature: 120 °C) for 14 h. The reaction mixture was allowed to cool down to room temperature. Water (10 mL) was added to the mixture, and the organic layer was extracted with $CHCl_3$ (15 mL×3). The organic extract was dried over Na_2SO_4 and filtered. Solvents in the filtrate were evaporated under reduced pressure to give crude solid (50 mg). The solid residue was purified by flash column chromatography on NH silica gel (eluent: *n*-hexane:EtOAc = 8:2). The collected fractions were combined, and the solvents were evaporated under reduced pressure to give an orange solid (22 mg), which was then reprecipitated from $CHCl_3$/*n*-hexane (1:2) to provide the title compound **2** in 42% yield (16.0 mg, 0.021 mmol) as an orange solid. Mp 221.2 °C; R_f 0.75 ($CHCl_3$, NH silica); ^{1}H NMR (400 MHz, $CDCl_3$) δ 10.7 (s, 2H), 8.19 (d, J = 9.2 Hz, 2H), 8.11 (d, J = 8.8 Hz, 4H), 8.08 (s, 2H), 8.02 (d, J = 9.2 Hz, 2H), 7.32 (dd, J = 8.4, 7.6 Hz, 8H), 7.23 (d, J = 8.8 Hz, 4H), 7.20 (d, J = 7.6 Hz, 8H), 7.09 (t, J = 7.2 Hz, 4H); ^{13}C NMR (100 MHz, $CDCl_3$) δ 156.1, 149.1, 147.3, 142.8, 141.1, 138.5, 132.4, 131.8, 131.1, 129.4, 128.2, 125.0, 123.5, 123.3, 122.9, 116.1 (1C was not detected in the aromatic region, probably due to the overlap of signals); IR (ATR, cm^{-1}): 3030, 1587, 1510, 1487, 1456, 1329, 1314, 1273, 1177, 1152, 1111, 984, 833, 750; MS (EI$^+$, direct): *m/z* (relative intensity, %) 769 ([M+1]$^+$, 67), 768 (M$^+$, 100), 691 ([M–Ph]$^+$, 2), 384 ([M/2]$^+$, 32); HRMS (EI$^+$, direct): *m/z* calcd for $C_{54}H_{36}N_6$ (M) 768.3001, found 768.3010. Elemental analysis (%) calcd. for $C_{54}H_{36}N_6$: C 84.35, H 4.72, N 10.93, found: C 84.25, H 4.58, N 10.66.

*Synthesis of 4,4'-(dibenzo[a,j]phenazine-3,11-diyl)bis(N,N-diphenylaniline) (**3**)*

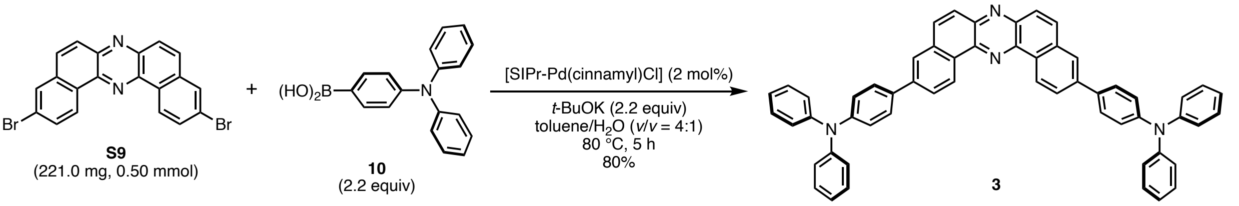

(S13)

To a flame-dried two-necked tube (10 mL) equipped with a magnetic stir bar, were added 3,11-dibromodibenzo[*a,j*]phenazine (**S9**) (221.0 mg, 0.50 mmol) and (4-(diphenylamino)phenyl)boronic acid (**10**) (335.0 mg, 1.1 mmol, 2.2 equiv). The tube was transferred into a glove box, where *t*-BuOK (124.0 mg, 1.1 mmol, 2.2 equiv) and [SIPr-Pd(cinnamyl)Cl] (7.0 mg, 10 μmol, 2 mol%) were added to the tube. The tube was sealed with two septa and taken from the glovebox, and deoxygenated toluene (5.0 mL) through freeze-throw cycles (3 times) and distilled water (1.0 mL) was added to the flask through a septum with a syringe. The mixture was purged with nitrogen gas and stirred in a Personal Organic Synthesizer (EYELA, *Chemi-Station*) equipped with a cooling system (aluminum block temperature: 80 °C) for 5 h. The reaction mixture was allowed to cool down to room temperature. Water (10 mL) was added to the mixture, and the organic layer was extracted with $CHCl_3$ (15 mL×3). The organic extract was dried over Na_2SO_4 and filtered. Solvents in the filtrate were evaporated under reduced pressure to give crude solid (515 mg). The solid residue was purified by flash column chromatography on NH silica gel (eluent: *n*-hexane:$CHCl_3$ = 9:1 to 8:2). The collected fractions were combined, and the solvents were evaporated under reduced pressure to give a yellow solid (364 mg), which was then reprecipitated from $CHCl_3$/*n*-hexane (1:2) to provide the title compound **3** in 80% yield (308.0 mg, 0.40 mmol) as a yellow solid. Mp 199.4 °C; R_f 0.75 (*n*-hexane/ $CHCl_3$ 2:8, NH silica); ^{1}H NMR (400 MHz, $CDCl_3$) δ 9.66 (d, J = 8.8 Hz, 2H), 8.16−8.08 (m, 8H), 7.71 (d, J = 8.0 Hz, 4H), 7.33−7.29 (m, 8H), 7.25−7.18 (m, 12H), 7.08 (t, J = 7.2 Hz, 4H); ^{13}C NMR (100 MHz, $CDCl_3$) δ 147.8, 147.5, 142.6, 141.4, 140.6, 134.0, 133.6, 132.5, 129.8, 129.4, 128.2, 127.3, 126.3, 125.7, 125.5, 124.7, 123.7, 123.2; IR (ATR, cm^{-1}): 3023, 1587, 1510, 1487, 1472, 1352, 1329, 1314, 1271, 1192, 1179, 991, 839, 812, 795, 750, 733; MS (EI$^+$, direct): *m/z* (relative intensity, %): 767 ([M+1]$^+$, 62), 766 (M$^+$, 100), 383 ([M/2]$^+$, 41); HRMS (EI$^+$): *m/z* calcd for $C_{54}H_{38}N_4$ (M) 766.3096, found 766.3082.

Single Crystal X-Ray Crystallographic Analysis

Crystallographic analysis of compound **8**: The single crystal suitable for the X-ray crystallographic analysis was grown from a MeOH solution by slow evaporation. A yellow needle crystal with dimensions of 0.200 × 0.100 × 0.100 mm^3 was mounted on a suitable support. Data were collected using an Rigaku XtaLAB P200 diffractometer with graphite monochromated CuKα radiation (λ = 1.54184 Å) to a $2\theta_{max}$ value of 148.7° at 193 K. The cell refinements were performed with a software CrysAlisPro 1.171.39.20a.[S8] The crystal structure was solved by direct methods (SHELXT Version 2018/3).[S9] All calculations were performed with the observed reflections [I > 2σ(I)] with the program Olex2 1.3 crystallographic software packages,[S10] except for refinement which was performed by SHELXL.[S11] The non-hydrogen atoms were refined anisotropically, and hydrogen atoms were refined using the riding model. The crystal data are summarized in Table S1. CCDC-2427517 contains the supplementary crystallographic data for **8**, which are available free of charge from the Cambridge Crystallographic Data Center (CCDC) via www.ccdc.cam.ac.uk/data_request/cif.

Crystallographic analysis of compound **1•TFMSA**: The single crystal suitable for the X-ray crystallographic analysis was grown from a toluene solution by slow evaporation. A yellow needle crystal with dimensions of 0.150 × 0.050 × 0.050 mm^3 was mounted on a suitable support. Data were collected using an Rigaku XtaLAB P200 diffractometer with graphite monochromated CuKα radiation (λ = 1.54184 Å) to a $2\theta_{max}$ value of 134.2° at 213 K. The cell refinements were performed with a software CrysAlisPro 1.171.43.105a.[S12] The crystal structure was solved by direct methods (SHELXT Version 2018/3).[S9] All calculations were performed with the observed reflections [I > 2σ(I)] with the program Olex2 1.5 crystallographic software packages,[S10] except for refinement which was performed by SHELXL.[S11] The non-hydrogen atoms were refined anisotropically, and hydrogen atoms were refined using the riding model. The crystal data are summarized in Table S1. CCDC-2427519 contains the supplementary crystallographic data for **1•TFMSA**, which are available free of charge from the Cambridge Crystallographic Data Center (CCDC) via www.ccdc.cam.ac.uk/data_request/cif

Table S1. Summary of the crystallographic data of compound **8** and **1•TFMSA**.

Compound	**8**	**1•TFMSA**
	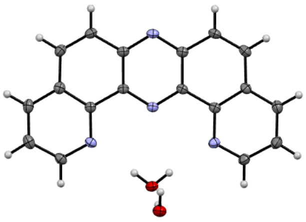	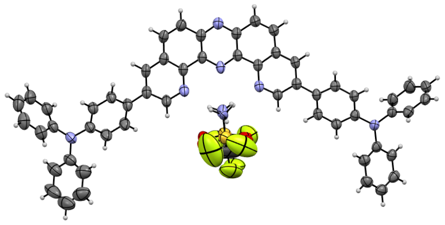
Empirical Formula	$C_{18}H_{14}N_4O_2$	$C_{55}H_{39}F_3N_7O_2S$
Formula Weight	318.33	918.99
Crystal System	*Triclinic*	*Monoclinic*
Space Group	*P-1* (#2)	*C12/c1* (#15)
Unit cell dimensions	a = 7.3062(2) Å α = 94.521(2)°	a = 46.279(5) Å α = 90°
	b = 10.1035(3) Å β = 100.326(2)°	b = 9.8612(3) Å β = 135.340(18)°
	c = 10.2334(3) Å γ = 99.364(2)°	c = 33.965(3) Å γ = 90°
V	728.72(4) Å^3	10895(3) Å^3
Z	2	8
Density (calculated)	1.451 g/cm^3	1.121 g/cm^3
Absorption	8.03 cm^{-1}	9.73 cm^{-1}
R_1 [I>2σ(I)]	0.0400	0.0619
wR_2 (all data)	0.1329	0.1996
Crystal size	0.200 × 0.100 × 0.100 mm^3	0.200 × 0.100 × 0.100 mm^3
Goodness-of-fit on F^2	1.066	0.997
Reflections	2857/7153 [R(int) = 0.0268]	10574/34808[R(int) = 0.0649]

Steady-State Photophysical Properties of 1–3 in Solution

All the steady-state UV-vis absorption and PL spectra were measured at room temperature with diluted solutions (10^{-5} M), which were prepared from degassed spectroscopic grade solvents (N_2 for 30 min). UV-vis absorption spectra were acquired with degassed solutions, while PL spectra were acquired with deaerated solutions prepared by freeze-throw method (3 cycles).

Emission lifetime measurement.

PL decay curves were deconvoluted with instrumental responsive function (IRF), and decay function G(t) was fitted with the following equation to extract lifetimes:

$$G(t) = \sum_{i=1}^{n} A_i \exp\left(-\frac{t}{\tau_i}\right)$$

where τ_i are lifetimes of each emission component and A_i are the corresponding fractional amplitudes.

Also, intensity average lifetime $<\tau>$ was calculated by the following equation:

$$<\tau> = \frac{\sum_{i=1}^{n} \tau_i{}^2 A_i}{\sum_{i=1}^{n} \tau_i A_i}$$

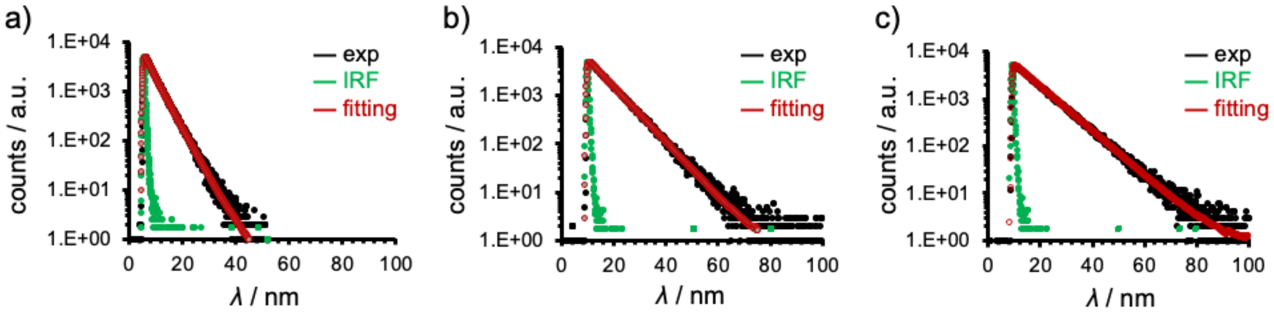

Figure S1. Emission decay profiles of **1** in a) toluene, b) THF, and c) $CHCl_3$. λ_{ex} = 365 nm. Black and green plots indicate experimental photocounting and instrumental responsive function (IRF), respectively. Red lines indicate fitting deconvolution curves.

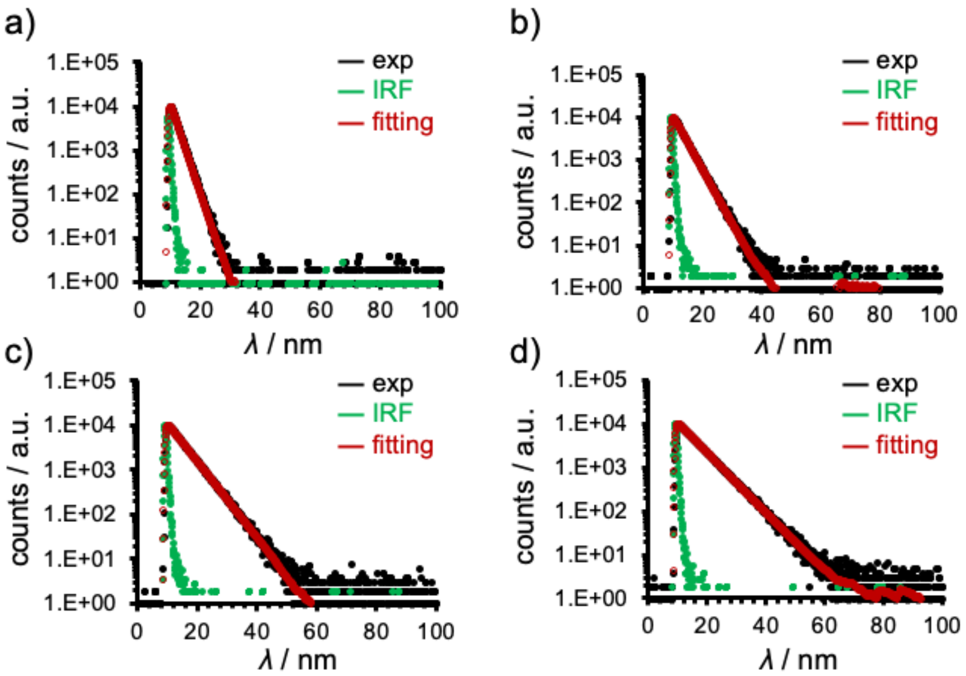

Figure S2. Emission decay profiles of **2** in a) cyclohexane, b) toluene, c) THF, and d) $CHCl_3$. λ_{ex} = 365 nm. Black and green plots indicate experimental photocounting and instrumental responsive function (IRF), respectively. Red lines indicate fitting deconvolution curves.

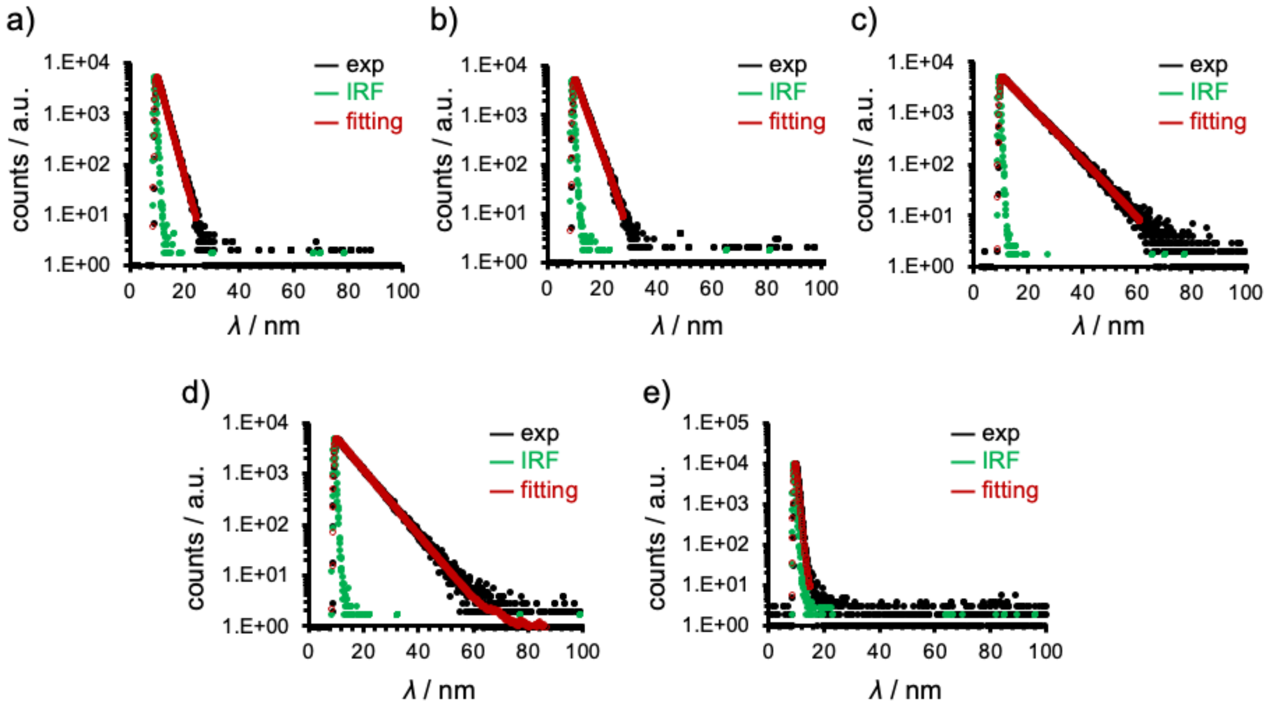

Figure S3. Emission decay profiles of **3** in a) cyclohexane, b) toluene, c) THF, d) $CHCl_3$, and e) DMF. λ_{ex} = 365 nm. Black and green plots indicate experimental photocounting and instrumental responsive function (IRF), respectively. Red lines indicate fitting deconvolution curves.

Absolute PLQY measurement

Absolute PLQYs were measured using a Hamamatsu Quantaurus-QY C11347-01 spectrometer equipped with an integrating sphere.

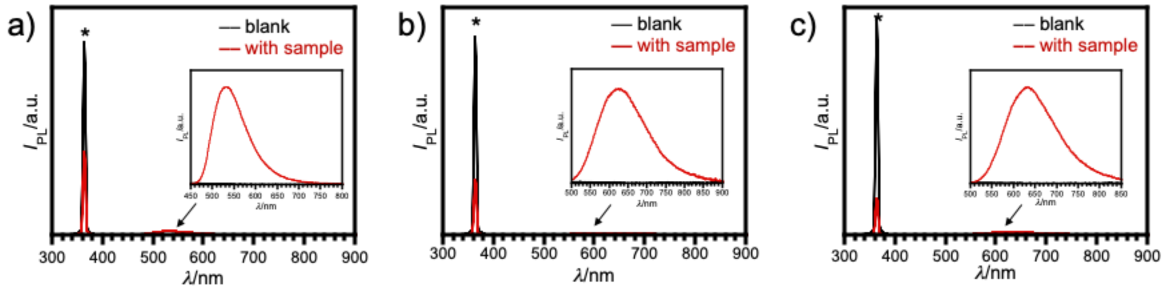

Figure S4. PL spectra of **1** recorded in a) toluene, b) THF, and c) $CHCl_3$ for absolute PLQY measurement (λ_{ex} = 365 nm). In each panel, the black solid line represents the blank (cuvette-only control), while the red solid line indicates the sample's photoluminescence. The asterisk marks the position of the residual excitation light. An inset shows an enlarged view of the emission region for clarity.

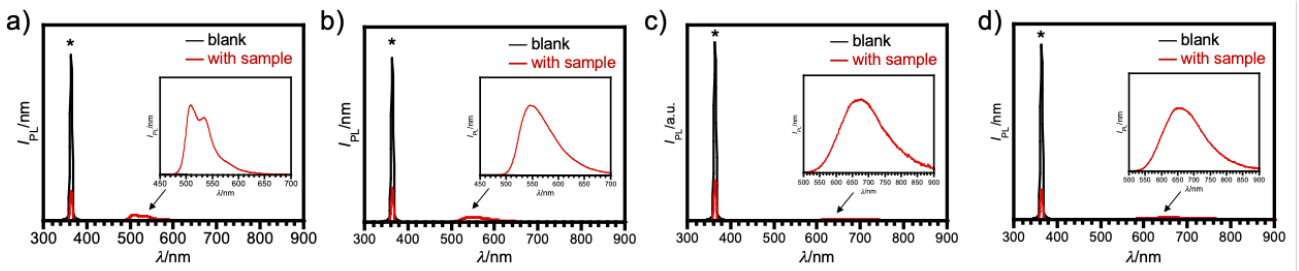

Figure S5. PL spectra of **2** recorded in a) cyclohexane, b) toluene, c) THF, and d) $CHCl_3$ for absolute PLQY measurement (λ_{ex} = 365 nm). In each panel, the black solid line represents the blank (cuvette-only control), while the red solid line indicates the sample's photoluminescence. The asterisk marks the position of the residual excitation light. An inset shows an enlarged view of the emission region for clarity.

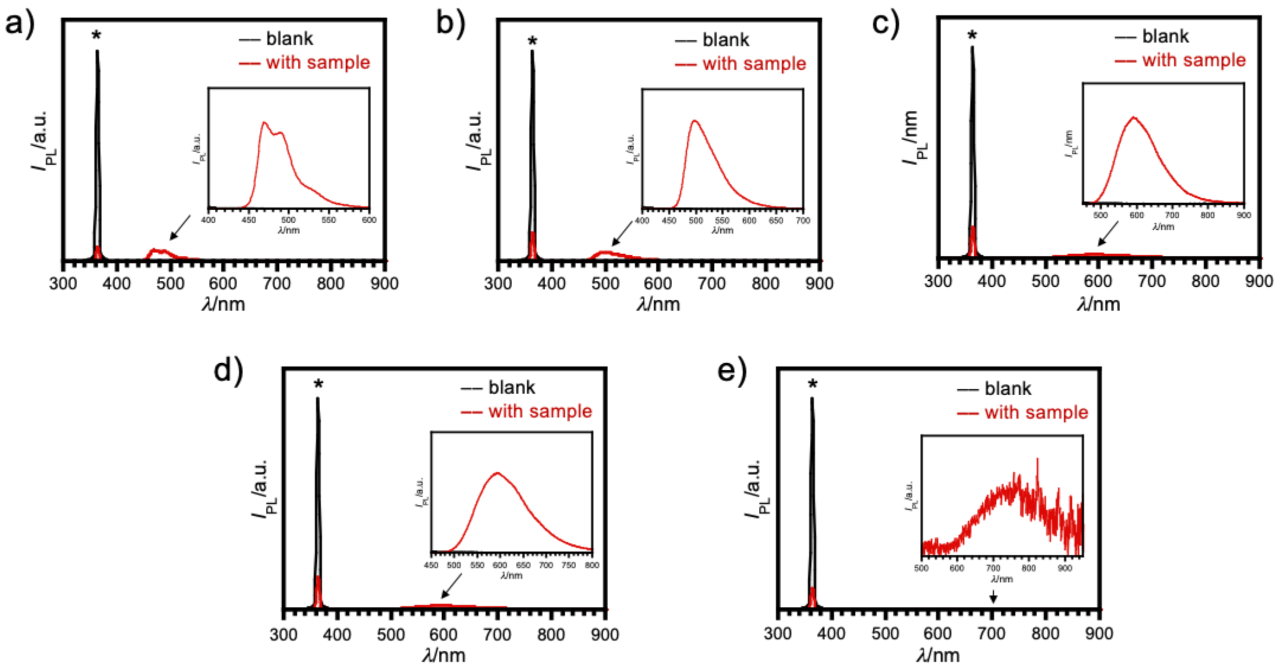

Figure S6. PL spectra of **3** recorded in a) cyclohexane, b) toluene, c) THF, d) $CHCl_3$, and e) DMF for absolute PLQY measurement (λ_{ex} = 365 nm). In each panel, the black solid line represents the blank (cuvette-only control), while the red solid line indicates the sample's photoluminescence. The asterisk marks the position of the residual excitation light. An inset shows an enlarged view of the emission region for clarity.

Table S2. Summary of photophysical properties of diluted solution of **1** (c = 10^{-5} M).

Solvent	λ_{abs} (nm)	ε ($M^{-1}cm^{-1}$)	λ_{em} (nm)[a]	Φ_{PL}[b]	$\langle\tau\rangle$ (ns)[c]	k_f (s^{-1})[d]
THF	444	26800	623	0.75	7.62	0.98×10^{8}
$CHCl_3$	446	22000	629	0.62	9.18	0.67×10^{8}

[a] Excited at λ_{ex} = 365 nm. [b] The absolute PLQY acquired with an integral sphere. [c] Intensity average lifetime. [d] fluorescence rate $k_f = \Phi_{PL}/\langle\tau\rangle$

Table S3. Summary of photophysical properties of diluted solution of **2** (c = 10^{-5} M).

Solvent	λ_{abs} (nm)	ε ($M^{-1}cm^{-1}$)	λ_{em} (nm)[a]	Φ_{PL}[b]	$\langle\tau\rangle$ (ns)[c]	k_f (s^{-1})[d]
cyclohexane	486	61500	502	0.53	2.20	2.41×10^{8}
			533		2.24	2.41×10^{8}
THF	473	42200	671	0.41	5.11	0.80×10^{8}
$CHCl_3$	479	51600	658	0.61	6.26	0.97×10^{8}

[a] Excited at λ_{ex} = 365 nm. [b] The absolute PLQY acquired with an integral sphere. [c] Intensity average lifetime. [d] fluorescence rate $k_f = \Phi_{PL}/\langle\tau\rangle$

Table S4. Summary of photophysical properties of diluted solution of **3** (c = 10^{-5} M).

Solvent	λ_{abs} (nm)	ε ($M^{-1}cm^{-1}$)	λ_{em} (nm)[a]	Φ_{PL}[b]	$\langle\tau\rangle$ (ns)[c]	k_f (s^{-1})[d]
cyclohexane	443	62300	464	0.58	2.23	2.60×10^{8}
			488		2.14	2.71×10^{8}
THF	446	50700	596	0.79	7.95	0.99×10^{8}
$CHCl_3$	448	40400	599	0.79	6.69	1.18×10^{8}
DMF	450	53000	774	0.03	0.56	0.54×10^{8}

[a] Excited at λ_{ex} = 365 nm. [b] The absolute PLQY acquired with an integral sphere. [c] Intensity average lifetime. [d] fluorescence rate $k_f = \Phi_{PL}/\langle\tau\rangle$

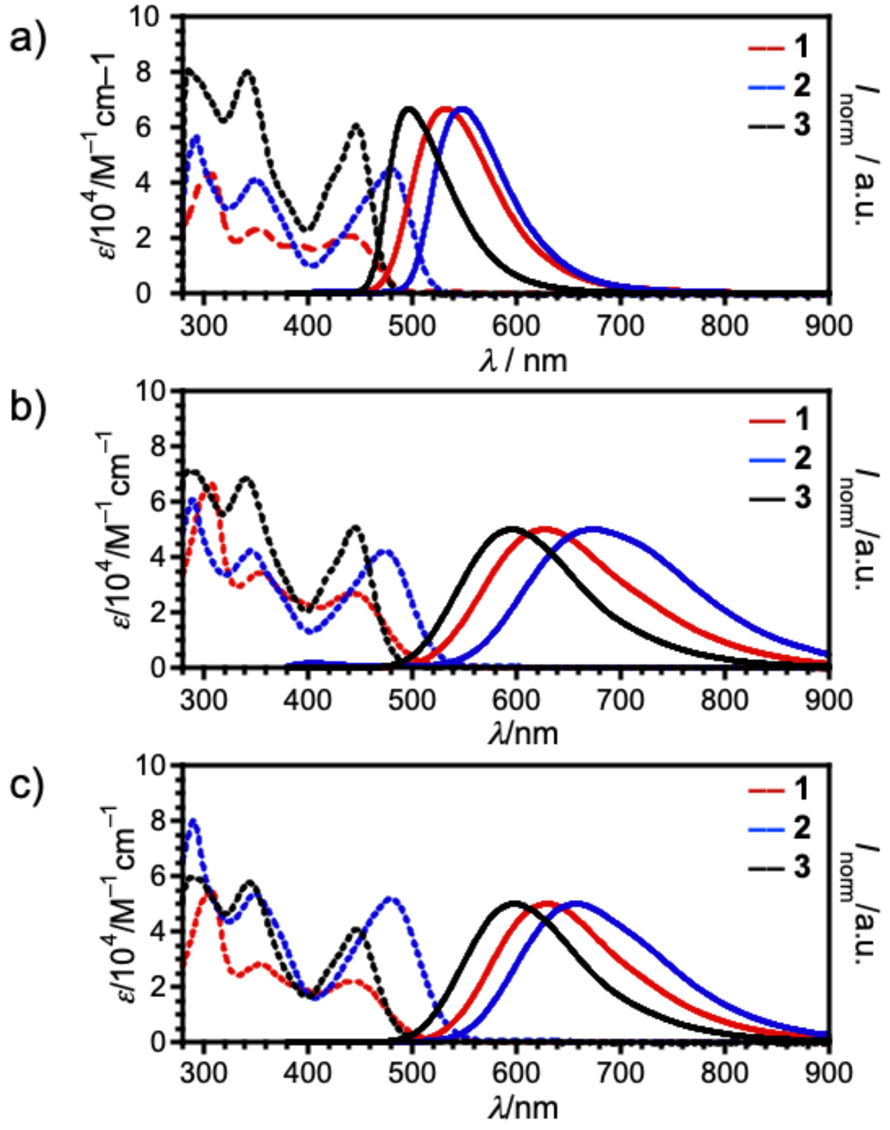

Figure S7. Comparison of UV-vis absorption and PL spectra of **1**–**3** in the same solvent: a) in toluene, b) in THF, and c) in $CHCl_3$.

Cyclic Voltammetry

The sample for CV measurement was prepared by dissolving compound **8** and (n-Bu)$_4$N•PF$_6$ electrolyte in a deaerated dichloromethane (c = 1.0 mM). Analysis was conducted at room temperature in a cell equipped with a Pt disc as the working electrode, Pt wire as the counter electrode, and Ag/AgNO$_3$ as the reference electrode under an Ar atmosphere (scanning rate: 50 mV/s). The HOMO/LUMO energy levels were calculated by the following equation using the onset potentials corrected against the Fc/Fc$^+$ (Fc = ferrocene) redox couple: IP = –HOMO = 5.1 + $^{ox}E_{onset}$/V [eV]; EA = –LUMO = 5.1 + $^{red}E_{onset}$/V [eV].

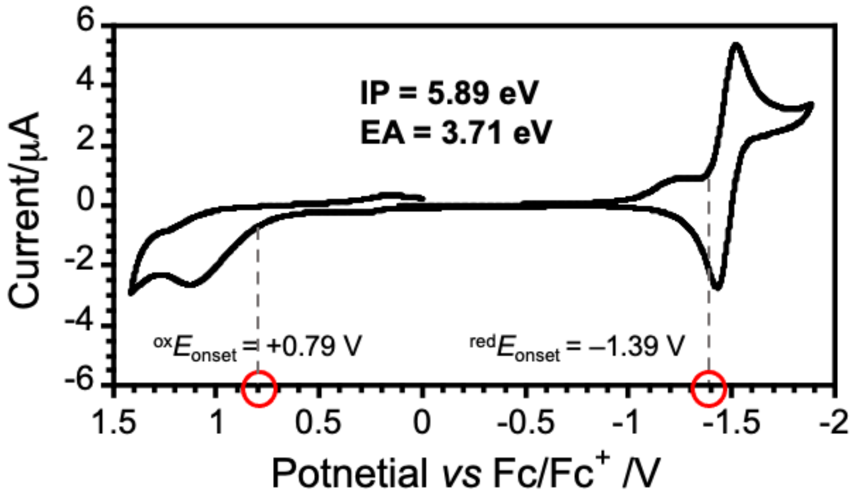

Figure S8. Cyclic voltammograms of **8** (1 mM) in CH$_2$Cl$_2$.

Job plot

Compound **1** (1.53 mg, 2.0 μmol) was placed into a 50 mL volumetric flask. Degassed spectroscopic grade toluene was added to dissolve the compound, and the solution was diluted to mark to achieve a concentration of 3.9 × 10$^{–5}$ M. Separately, NH$_2$SO$_2$CF$_3$ (1.45 mg, 9.7 μmol) was added into another 50 mL volumetric flask. Degassed spectroscopic grade toluene was added to dissolve the compound, and the solution was diluted to the mark to obtain a concentration of 2.0×10$^{–4}$ M. A 10 mL aliquot of the NH$_2$SO$_2$CF$_3$ stock solution was transferred to a third 50 mL volumetric flask and diluted to the mark with toluene, resulting in a solution with a concentration of 3.9×10$^{–5}$ M. In a quartz cuvette equipped with a magnetic stir bar, mixtures of compound **1** and NH$_2$SO$_2$CF$_3$ were prepared using volumetric pipettes to achieve a total volume of 3.0 mL. The initial molar ratios of [**1**]$_0$ to [NH$_2$SO$_2$CF$_3$]$_0$ were adjusted as follows: 10:0, 9:1, 8:2, 7:3, 6:4, 5:5, 4:6, 3:7, 2:8, 1:9, and 0:10. Each

mixture was stirred for 10 minutes at room temperature. Subsequently, the UV-Vis absorption spectrum of each solution was recorded.

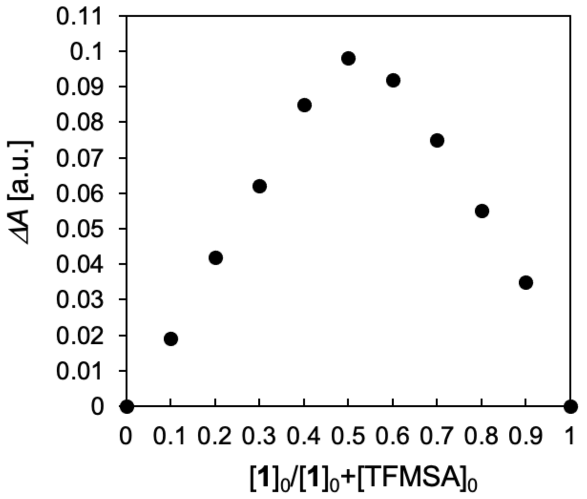

Figure S9. Job plot of **1**. Δ*A* denotes the difference between the absorbance at 500 nm after guest addition and the initial absorbance.

Photophysical properties of 1 with addition of TFMSA

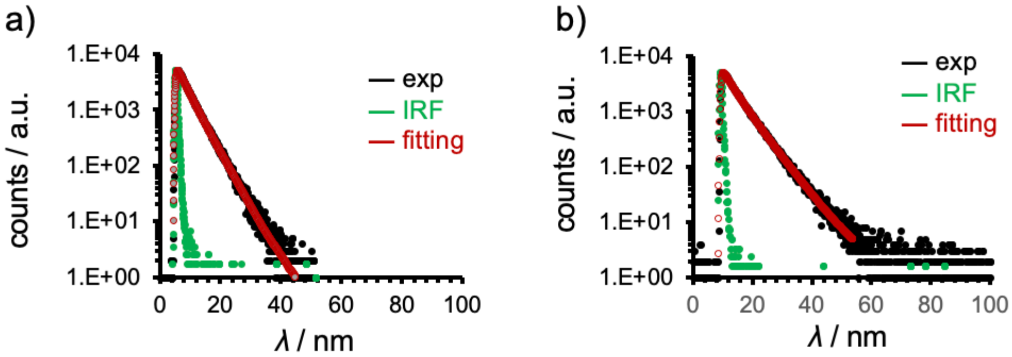

Figure S10. Emission decay profiles of a) **1** alone and b) **1** + TFMSA in toluene (λ_{ex} = 365 nm). Black and green plots indicate experimental photocounting and instrumental responsive function (IRF), respectively. Red lines indicate fitting deconvolution curves.

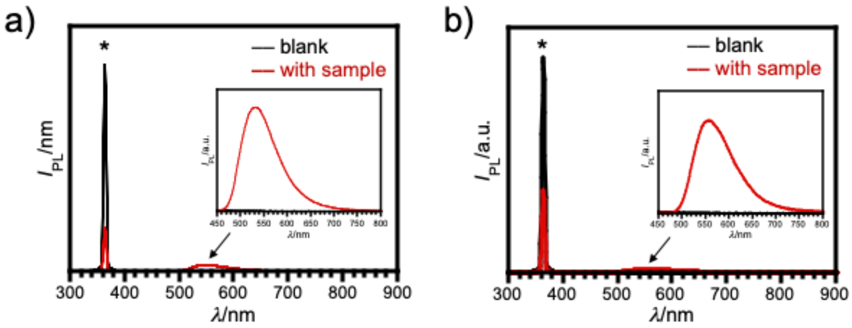

Figure S11. PL spectra of a) **1** alone and b) **1** + TFMSA in toluene for absolute PLQY measurement (λ_{ex} = 365 nm). In each panel, the black solid line represents the blank (cuvette-only control), while the red solid line indicates the sample's photoluminescence. The asterisk marks the position of the residual excitation light. An inset shows an enlarged view of the emission region for clarity.

UV-Vis Absorption and PL Titration Experiments

*UV-Vis Absorption Titration of **1** with TFMSA*: Dehydrated toluene (spectroscopic analysis grade) was deaerated through babbling N_2 gas for 30 min. To a volumetric flask (50 mL), were added **1** (199 µg, 0.26 µmol) and toluene to make 50 mL of solution (c = 5.2 µM) (*Solution A*). In another volumetric flask (20 mL), were added TFMSA (892 µg, 6.0 µmol) and the *Solution A* to make 20 mL of solution (the concentration of TFMSA = 0.3 mM) (*Solution B*). In a quartz cell (optical path length: 1 cm), were added *Solution A* (2.2 mL) and a stirring bar. The UV-Vis absorption spectrum was acquired. *Solution B* (4 µL, TFMSA 0.10 equiv) was added, and the resulting solution was stirred for 10 min, and UV-Vis absorption spectrum was acquired. In a similar manner, a portion of *Solution B* was added (8, 12, 16, 20, 24, 28, 36, 40, 48, 56, 64, 72, 80, 120, 200, and 400 µL), and the UV-Vis spectrum was acquired. Obtained titration curve was fitted with 1:1 binding model with bindfit using the Nelder-Mead method.[S13] The titration of **2** and **3** were conducted in a similar manner. The titration of **1** with other hydrogen bond donors were conducted in a similar manner.

*PL Titration of **1** with TFMSA*: Dehydrated toluene (spectroscopic analysis grade) was deaerated through babbling N_2 gas for 30 min. To a volumetric flask (25 mL), were added **1** (184 µg, 0.24 µmol) and toluene to make 25 mL of solution (c = 9.5 µM) (*Solution A*). In another volumetric flask (10 mL), were added TFMSA (933 µg, 6.3 µmol) and the *Solution A* to make 10 mL of solution (the concentration of TFMSA = 6.3 mM) (*Solution B*). In a quartz cell (optical path length: 1 cm) filled with N_2 gas, were added *Solution A* (2.5 mL) deaerated with freeze-threw cycles (3 times) and a stirring bar. The PL spectrum was acquired (λ_{ex} = 365 nm). *Solution B* (4 µL, TFMSA 0.10 equiv) was added, and the resulting solution was stirred for 10 min, and PL spectrum was acquired. In a similar manner,

a portion of *Solution B* was added (8, 12, 16, 20, 24, 28, 32, 36, 40, 80, 160, 240, 320, 400, 800, 2000, and 4000 μL), and the PL spectrum was acquired. The titration of **2** and **3** were conducted in a similar manner. The titration of **1** with other hydrogen bond donors were conducted in a similar manner.

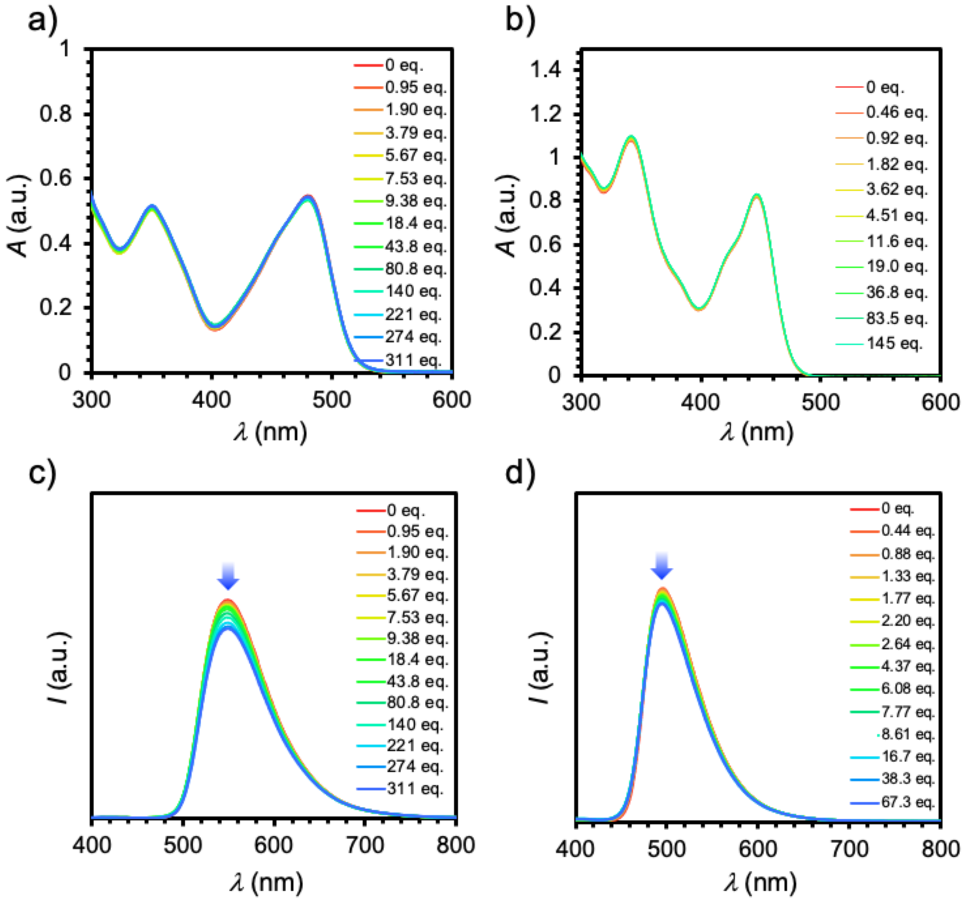

Figure S12. UV-vis absorption titration of a) **2** ($[H]_0 = 0.95\times10^{-5}$ M) and b) **3** ($[H]_0 = 1.1\times10^{-5}$ M) with TFMSA in toluene. PL titration of c) **2** ($[H]_0 = 0.95\times10^{-5}$ M) and d) **3** ($[H]_0 = 1.1\times10^{-5}$ M) with TFMSA in toluene (λ_{ex} = 365 nm).

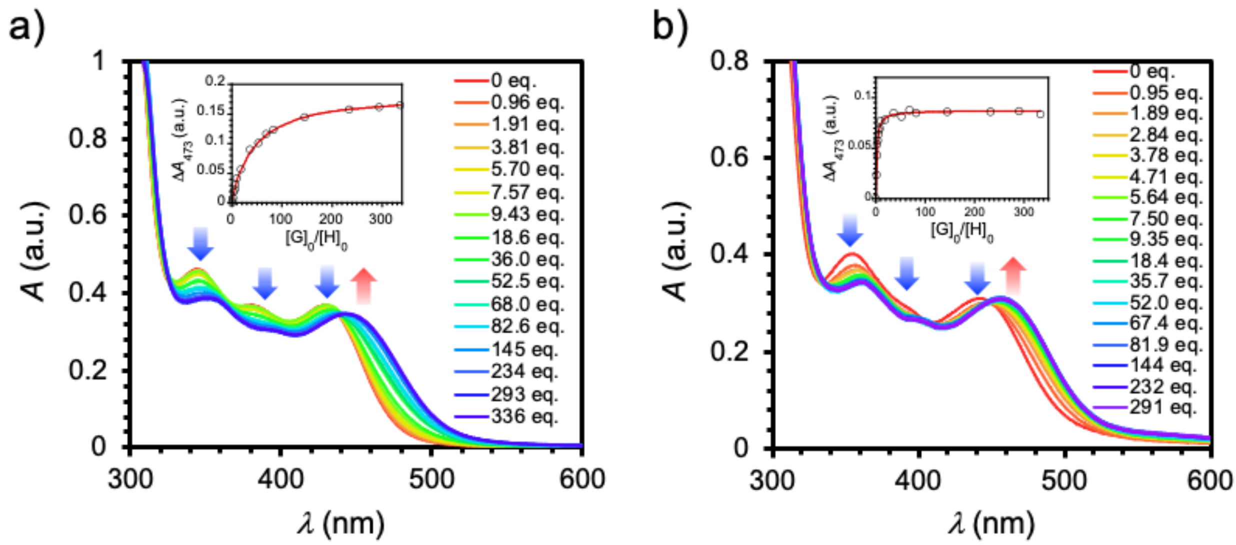

Figure S13. UV-vis absorption titration of **1** with TFMSA in a) THF ($[H]_0 = 1.06\times10^{-5}$ M) and b) $CHCl_3$ ($[H]_0 = 1.10\times10^{-5}$ M). The inserted graphs indicate the isotherm plots obtained by plotting the absorbance at 473 nm as the function of $[G]_0/[H]_0$.

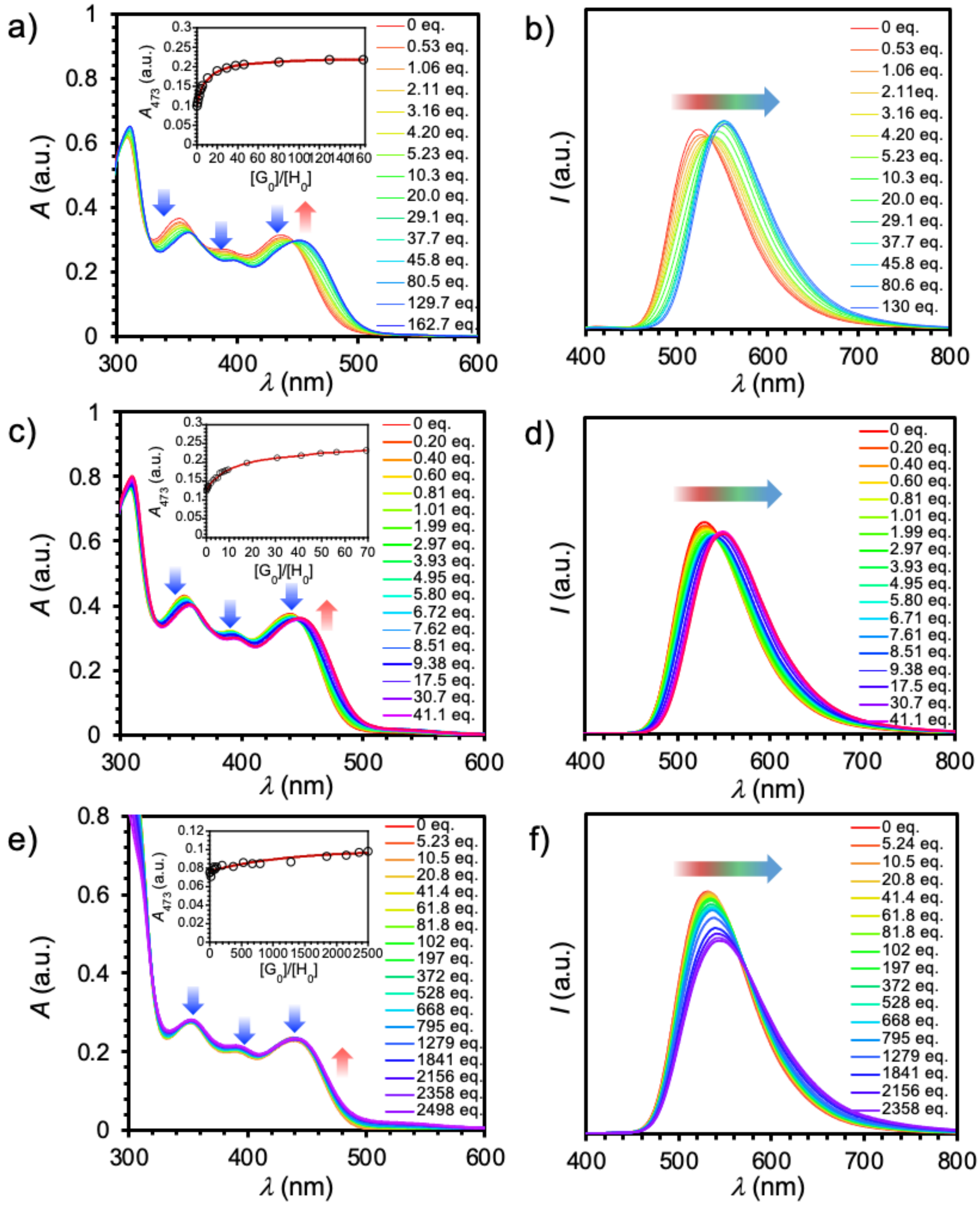

Figure S14. UV-vis absorption and PL (λ_{ex} = 365 nm) titration of **1** with a), b) methanesulfonamide ($[H]_0 = 9.77\times10^{-6}$ M), c), d) trifluoroacetamide ($[H]_0 = 9.78\times10^{-6}$ M), and e), f) acetamide ($[H]_0 = 1.05\times10^{-5}$ M) in toluene. The inserted graphs indicate the isotherm plots obtained by plotting the absorbance at 473 nm as the function of $[G]_0/[H]_0$.

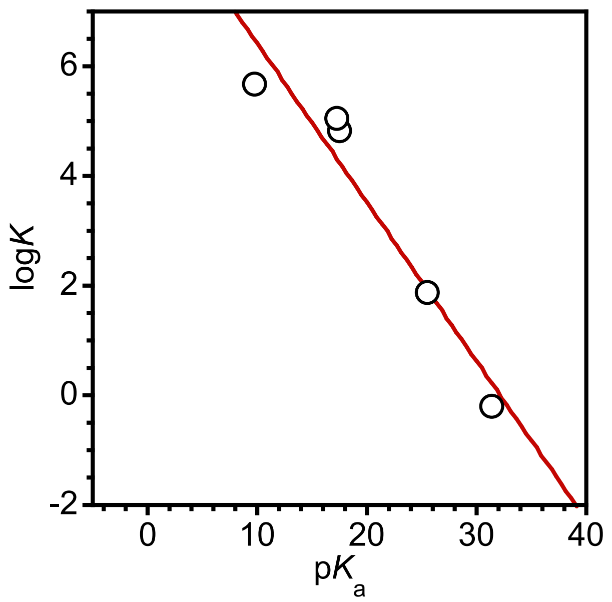

Figure S15. Relationship between log$K$ and pK_a of bidentate hydrogen bond donors.

Monitoring of ^{1}H NMR of 1 and TFMSA

To a vial (3 mL), were added **1** (2.32 mg, 3.0 μmol) and $CDCl_3$ (0.90 mL) (c = 3.4 mM, *Solution A*). To another vial (3 mL), were added TFMSA (482 μg, 3.2 μmol) and *Solution A* (0.3 mL) (the concentration of TFMSA = 1.1×10^{-2} M, *Solution B*). To a standard NMR tube, *Solution A* (0.60 mL) was added, and ^{1}H NMR spectrum was acquired. To the tube, 15 μL of *Solution B* (TFMSA 0.08 equiv) was added, and ^{1}H NMR spectrum was acquired. In a similar manner, a portion of *Solution B* was added (30, 60, 90, 120, 150, 180, 210, 240, and 270 μL), and the ^{1}H NMR spectrum was acquired.

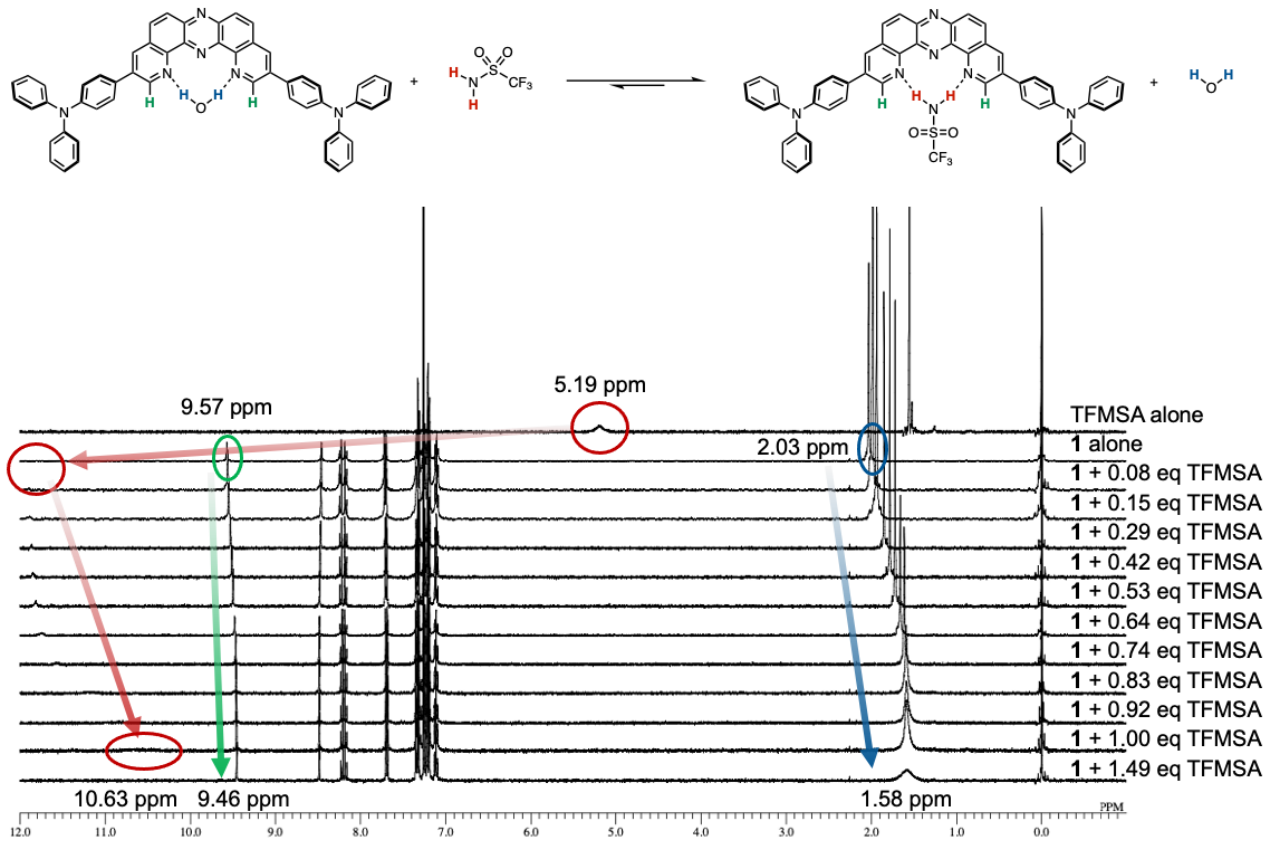

Figure S16. Change in ^{1}H NMR spectrum of **1** and TFMSA in $CDCl_3$ upon incremental addition of TFMSA from 0 to 1.49 equivalent.

^{1}H NMR Titration of 1 with Water

To a dried NMR tube, were added dried **1** (150 °C for 10 h in a vacuum oven), dried $CDCl_3$ over molecular sieves 3 Å (0.626 mL), and 1,1,2,2-tetrachloroethane (11.8 mg, 0.0703 mmol) (*Solution A*). ^{1}H NMR of *Solution A* was acquired, and the amount of **1** was determined to be 6.56 µmol based on the are integration of spectrum compared with an internal standard (1,1,2,2-tetracholoroethane). To a wet $CDCl_3$ (3.96 mL), 1,1,2,2-tetrachloroethane (18.2 mg, 0.1084 mmol) was added. From the ^{1}H NMR spectrum, water concentration of the wet $CDCl_3$ was determined to be 8.39 mM. To the *Solution A*, 78 µL of the wet $CDCl_3$ (H_2O 0.1 equiv) was added, and ^{1}H NMR was acquired. In a similar manner, a portion of the wet $CDCl_3$ was added (156, 234, 312, 390, 1170, 1950, 2730, 3510, 4290, 5070, 5850, 6630, and 7410 µL), and the ^{1}H NMR spectrum was acquired. Obtained titration curve was fitted with 1:1 binding model with bindfit using the Nelder-Mead method.[S13]

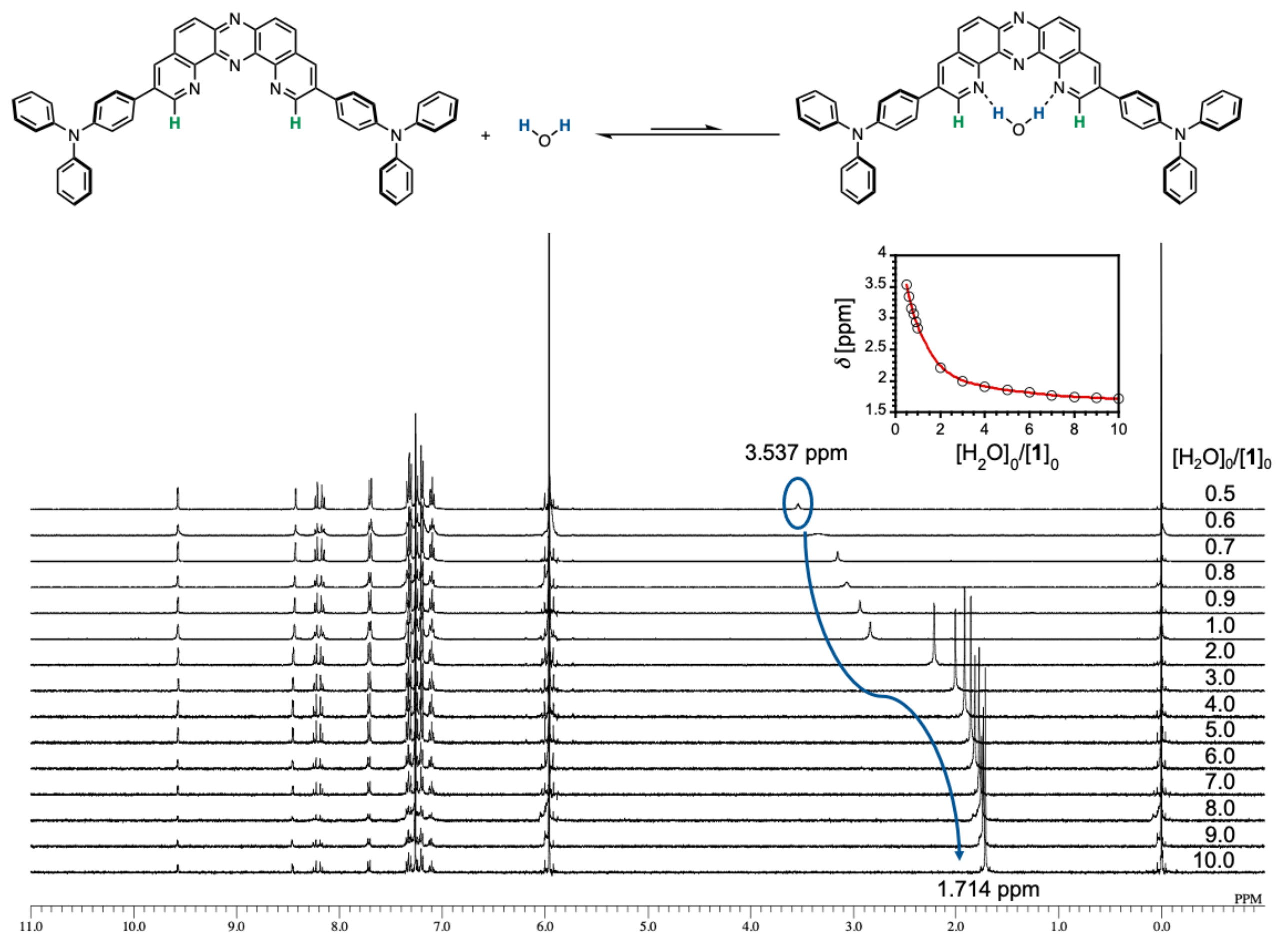

Figure S17. ^{1}H NMR titration of **1** with water in $CDCl_3$. The inset graph illustrates titration curve plotted with chemical shift of water.

Preparation of Emitter-Doped Films

*For the preparation of **1**@PS film*: To a vial (3 mL), were added toluene (spectroscopic analysis grade, 0.30 mL), compound **1** (0.724 mg, 0.94 mmol), and polystyrene (PS) (average Mw 35,000, 70 mg). The mixture was dissolved under irradiation of ultrasonication to make homogeneous solution (concentration of **1** against PS = 1 wt%). The solution was dropped onto a quartz plate (1 cm×1 cm), and film was fabricated by a spin-coat method (500 rpm over 60 sec; 500 rpm for 60 sec; 3000 rpm over 60 sec; 3000 rpm for 60 sec; 0 rpm over 60 sec). The obtained film was dried in a vacuum oven at 120 °C for 4 h. The films containing **2** and **3** were prepared in a similar manner.

*For the preparation of **1**+TFMSA@PS film*: To a vial (3 mL), were added toluene (spectroscopic analysis grade, 0.30 mL), compound **1** (0.750 mg, 0.98 mmol), TFMSA (0.152 mg, 1.0 mmol), and polystyrene (PS) (average Mw 35,000, 70 mg). The mixture was dissolved under irradiation of ultrasonication to make homogeneous solution (concentration of **1** against PS = 1 wt%). The solution was dropped onto a quartz plate (1 cm×1 cm), and film was fabricated by a spin-coat method (500 rpm

over 60 sec; 500 rpm for 60 sec; 3000 rpm over 60 sec; 3000 rpm for 60 sec; 0 rpm over 60 sec). The obtained film was dried in a vacuum oven at 120 °C for 4 h. The films containing **2** and **3** with TFMSA were prepared in a similar manner.

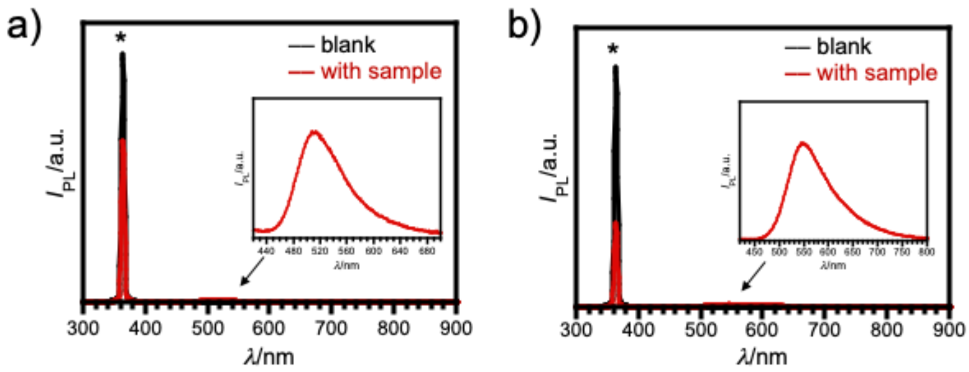

Figure S18. PL spectra of a) **1**@PS (1 wt%) and b) **1**+TFMSA@PS (1 wt%, 1:1) for absolute PLQY measurement (λ_{ex} = 365 nm). In each panel, the black solid line represents the blank (cuvette-only control), while the red solid line indicates the sample's photoluminescence. The asterisk marks the position of the residual excitation light. An inset shows an enlarged view of the emission region for clarity.

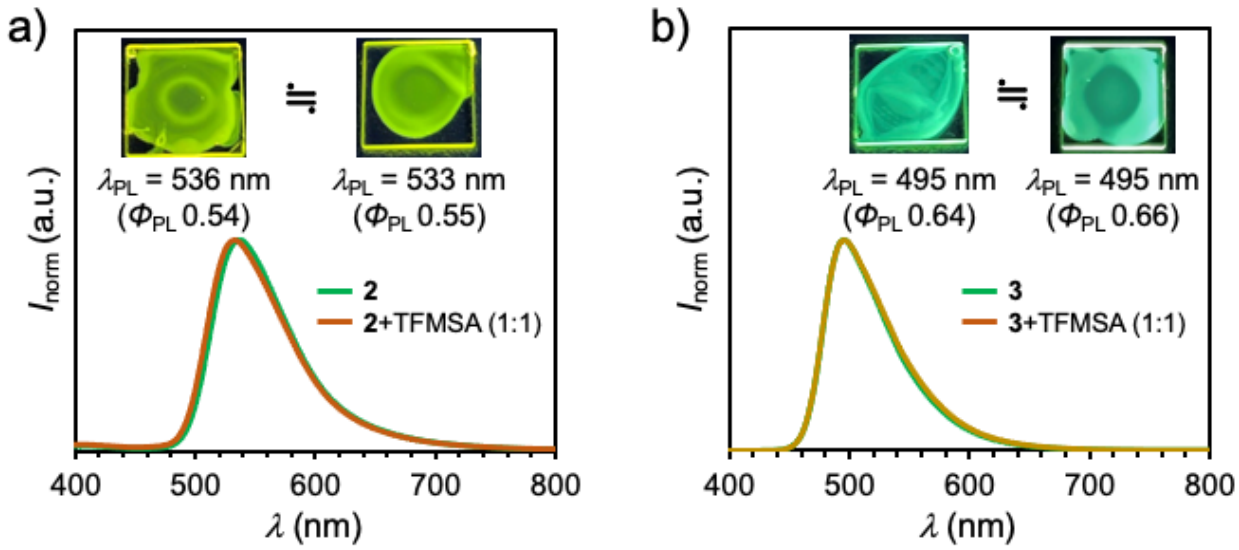

Figure S19. PL spectra of a) **2**@PS (1 wt%) and **2**+TFMSA@PS (λ_{ex} = 365 nm); b) **3**@PS (1 wt%) and **3**+TFMSA@PS (λ_{ex} = 365 nm).

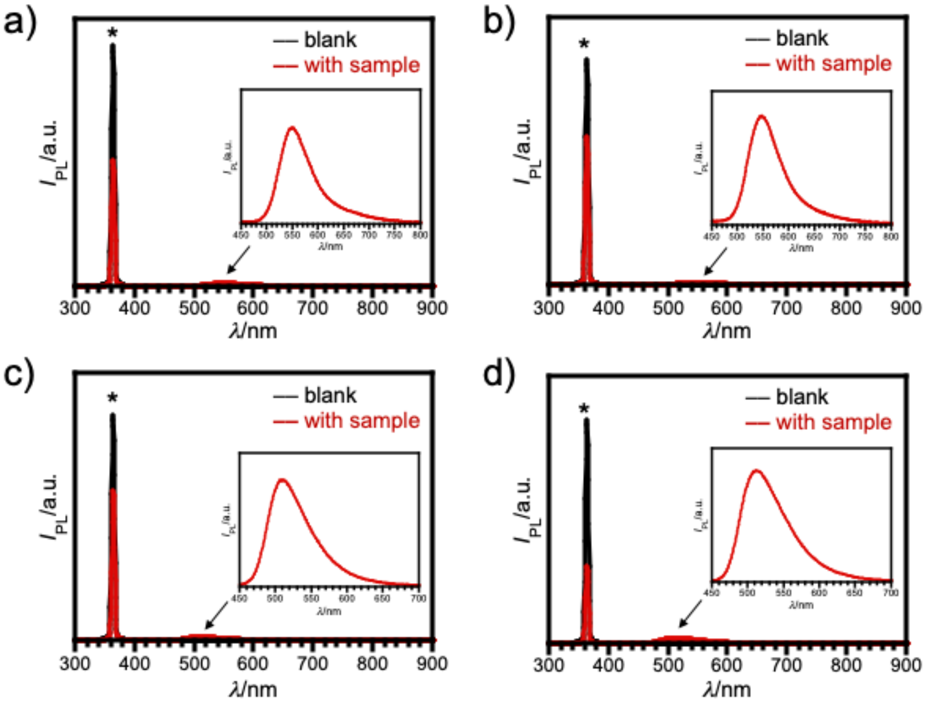

Figure S20. PL spectra of a) **2**@PS (1 wt%), b) **2**+TFMSA@PS (1 wt%, 1:1), c) **3**@PS (1 wt%), and d) **3**+TFMSA@PS (1 wt%, 1:1) for absolute PLQY measurement (λ_{ex} = 365 nm). In each panel, the black solid line represents the blank (cuvette-only control), while the red solid line indicates the sample's photoluminescence. The asterisk marks the position of the residual excitation light. An inset shows an enlarged view of the emission region for clarity.

Femtosecond-to-Nanosecond Transient Absorption Spectroscopy

Transient absorption measurements on femtosecond to nanosecond timescales were conducted by a homemade pump-probe system. An amplified femtosecond laser, Spirit One 1040-8 (Spectra-Physics, 1040 nm, the pulse width: ~270 fs), was split into two beams with a ratio of 1:9. The stronger beam was passed through a BBO crystal (type I, θ = 23.2°, 5 × 5 × 6 mm^3, Optronscience, Inc., Japan) to generate a second harmonic (520 nm). Then, the fundamental (1040 nm) and the second harmonic beams were separated by a beam splitter. A fundamental beam was passed through a half-wave plate to match the polarization to the second harmonic. Each beam passes through different optical pathways and was directed to another BBO crystal (type I, θ = 32.1°, 5 × 5 × 1 mm^3, Optronscience, Inc., Japan)) to generate a third harmonic (347 nm) for the pump beam. The pump beam was chopped before the sample at 500 Hz for signal differencing. The other weaker beam was focused on deuterated water placed in a 10-mm quartz cuvette to generate the white light continuum for the probe beam. Both pump and probe beams were focused on the sample solution placed in the 2-mm quartz cuvette. The polarization between the pump and probe pulses was set at a magic angle. The transmitted probe beam was detected with a multichannel detection system, PK120-C-RK (UNISOKU), composed of a CMOS linear image sensor and a polychromator. The obtained spectra were calibrated for group velocity dispersion using the data obtained by the optical Kerr signal of CH_2Cl_2 between the pump pulse and the white-light continuum. The instrumental response function was shorter than approximately 100 fs. The sample solutions were stirred with a stirrer during the experiments. The measurements were performed at room temperature.

Microsecond Transient Absorption Spectroscopy

Microsecond transient absorption measurements were conducted using a TSP–2000 time-resolved spectrophotometer (Unisoku). The third harmonic (355 nm) of a 10 Hz Q-switched Nd:YAG laser (~5 ns pulse, Minilite II, Amplitude Japan) was used as the excitation light and the laser pulse was irradiated to the sample placed in a 10-mm quartz cuvette without a defocusing lens under nitrogen atmosphere. The measurements were performed in toluene or chloroform solutions placed in a 10-mm quartz cuvette under nitrogen conditions at room temperature.

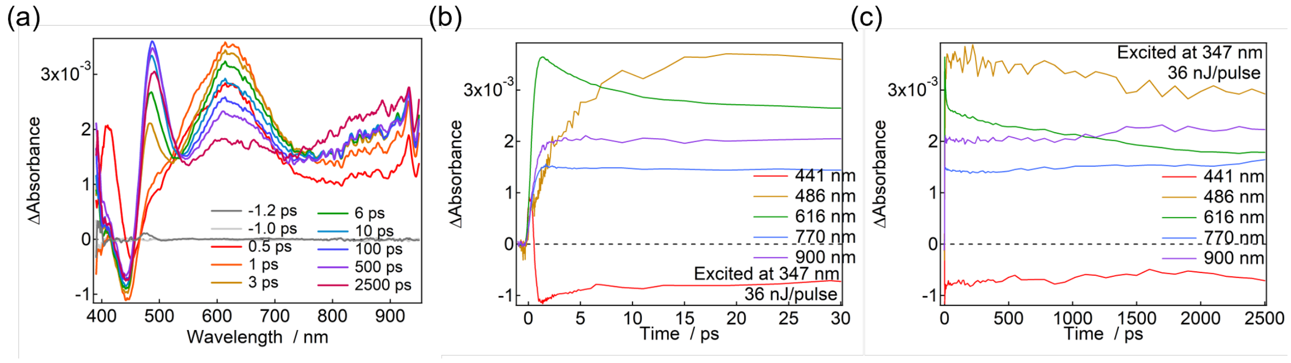

Figure S21. Transient absorption (a) spectra and (b, c) dynamics of **1** in toluene excited with a 347-nm pulse (36 nJ pulse^{-1}).

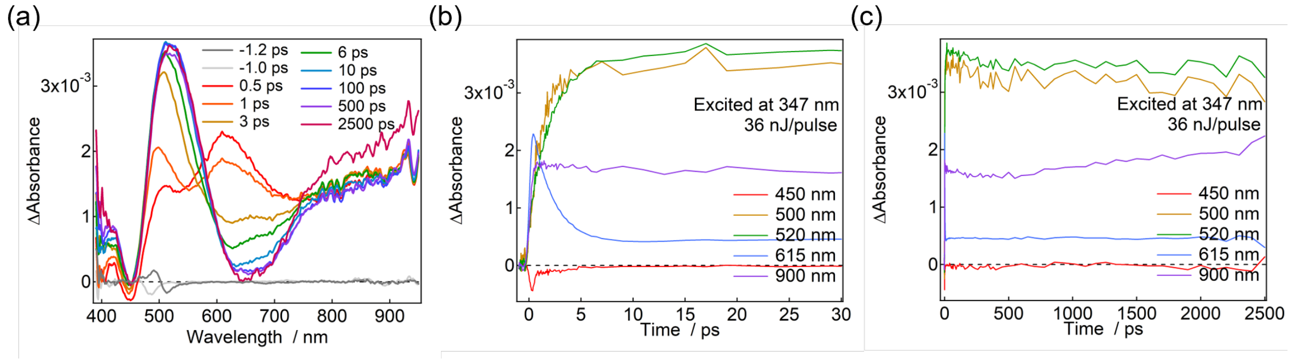

Figure S22. Transient absorption (a) spectra and (b, c) dynamics of **1** in chloroform excited with a 347-nm pulse (36 nJ pulse^{-1}).

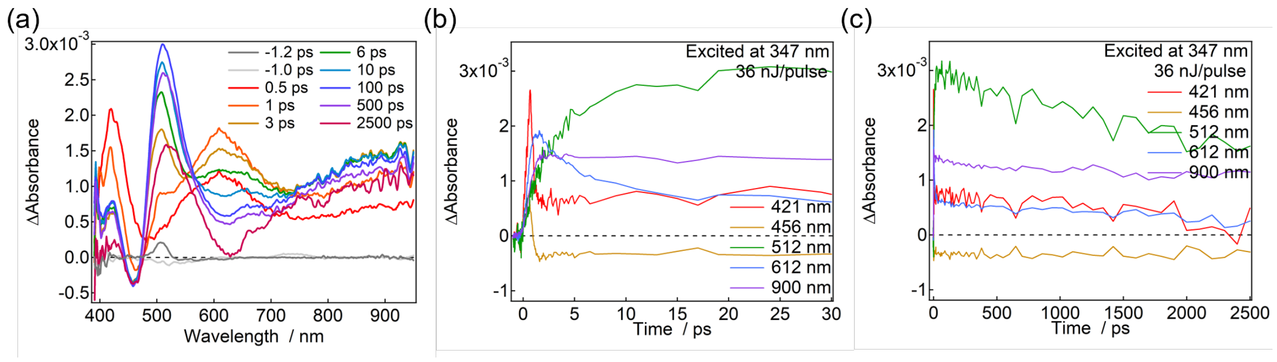

Figure S23. Transient absorption (a) spectra and (b, c) dynamics of **1** with TFMSA (1:235) in toluene excited with a 347-nm pulse (36 nJ pulse^{-1}).

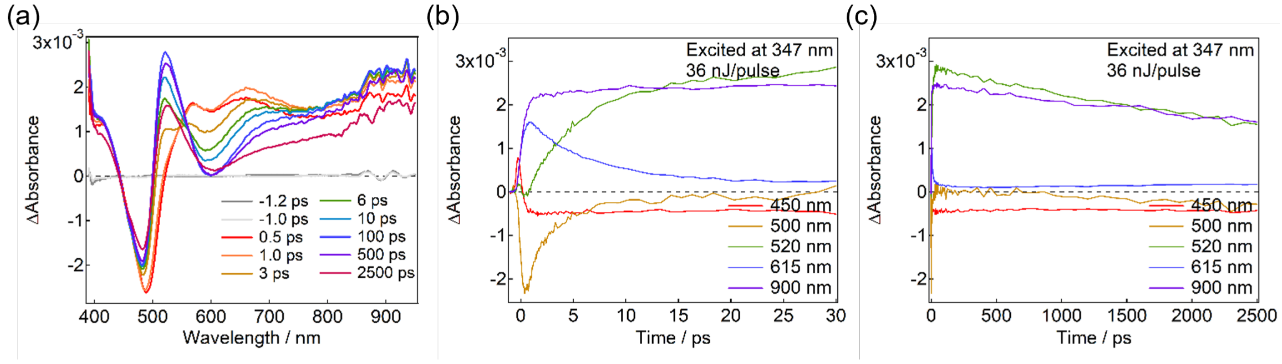

Figure S24. Transient absorption (a) spectra and (b, c) dynamics of **2** in toluene excited with a 347-nm pulse (36 nJ pulse^{-1}).

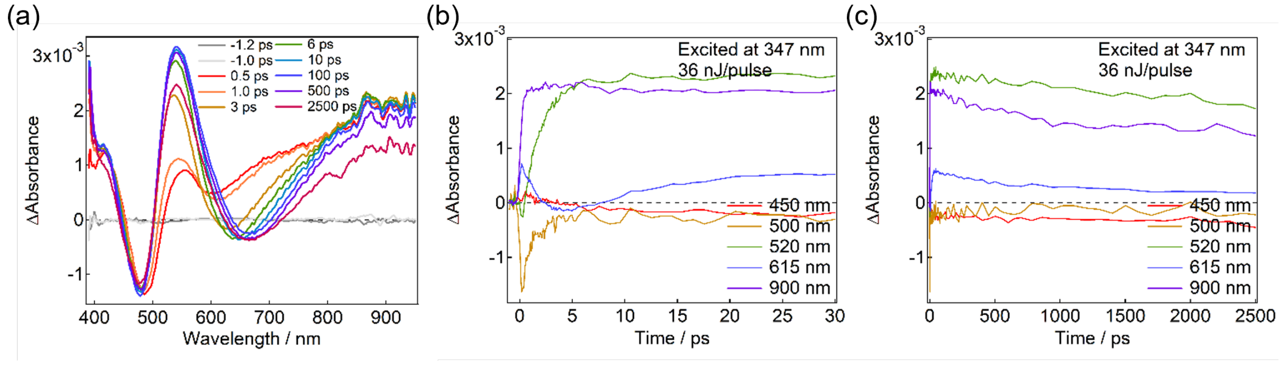

Figure S25. Transient absorption (a) spectra and (b, c) dynamics of **2** in chloroform excited with a 347-nm pulse (36 nJ pulse^{-1}).

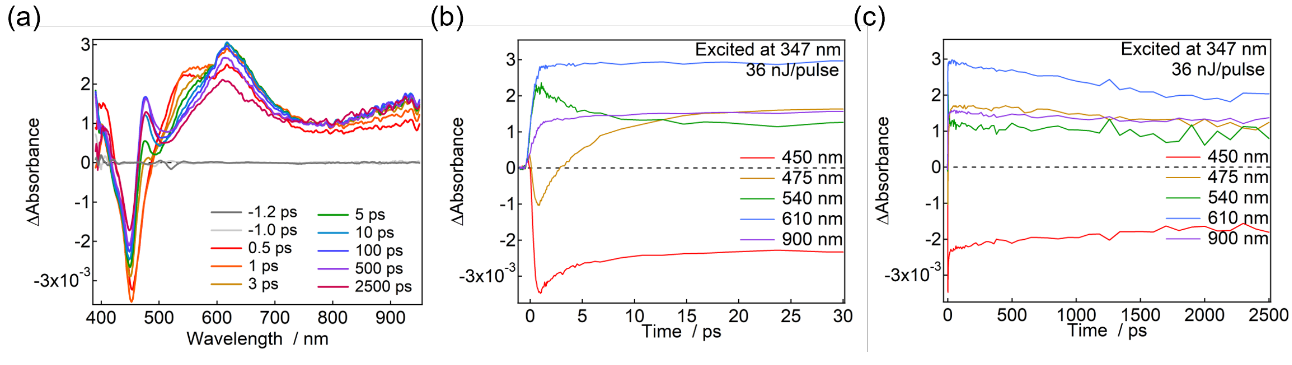

Figure S26. Transient absorption (a) spectra and (b, c) dynamics of **3** in toluene excited with a 347-nm pulse (36 nJ pulse^{-1}).

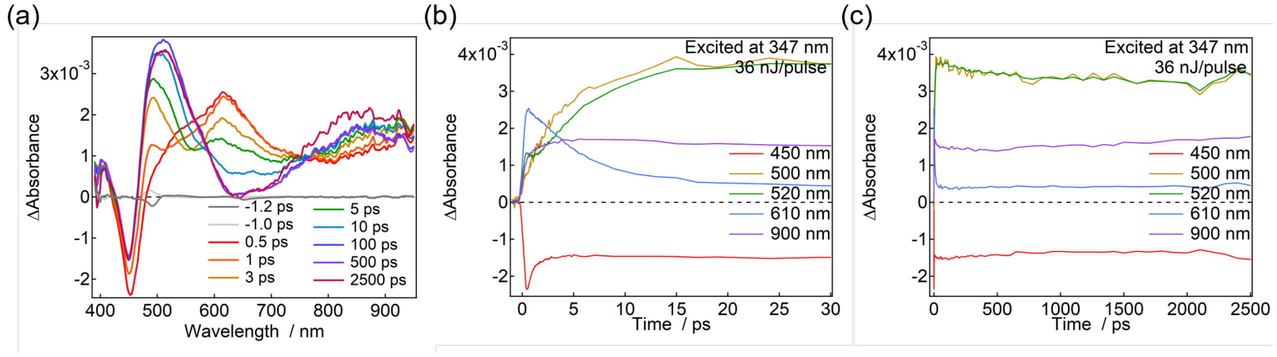

Figure S27. Transient absorption (a) spectra and (b, c) dynamics of **3** in chloroform excited with a 347-nm pulse (36 nJ pulse^{-1}).

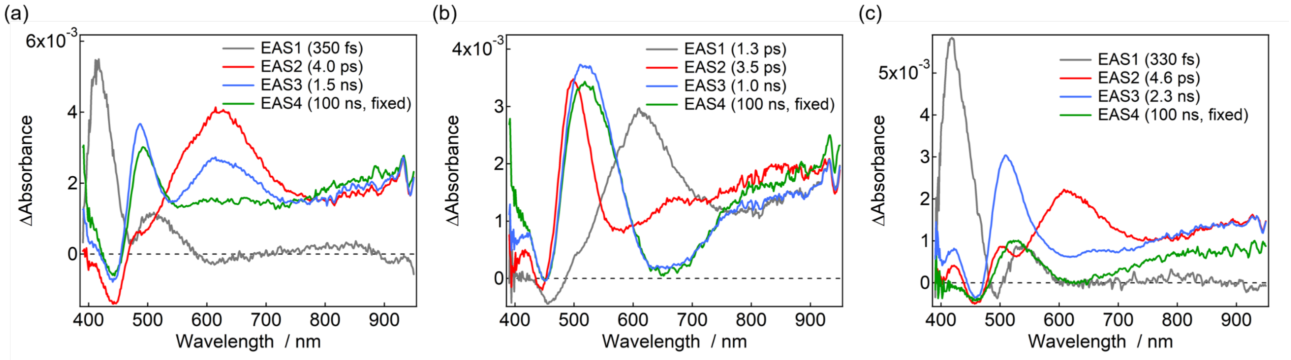

Figure S28. Evolution associated spectra (EAS) of the transient absorption spectra of **1** in (a) toluene (b) chloroform (c) toluene with TFMA (1:235) excited with a 347-nm pulse (36 nJ pulse^{-1}).

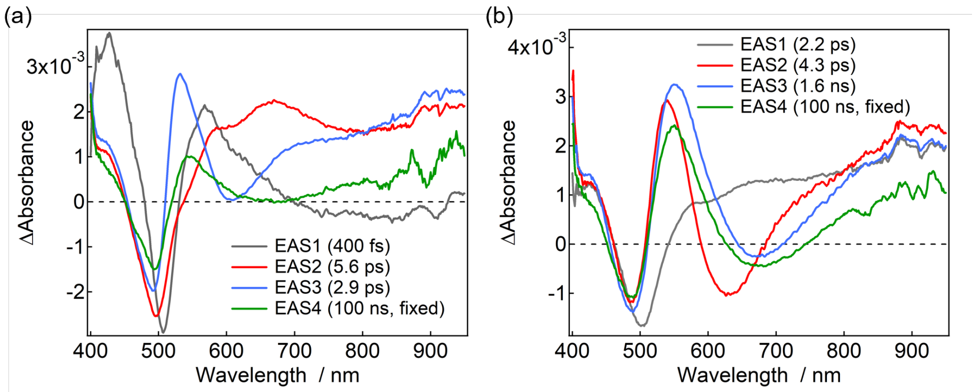

Figure S29. Evolution associated spectra (EAS) of the transient absorption spectra of **2** in (a) toluene (b) chloroform excited with a 347-nm pulse (36 nJ pulse^{-1}).

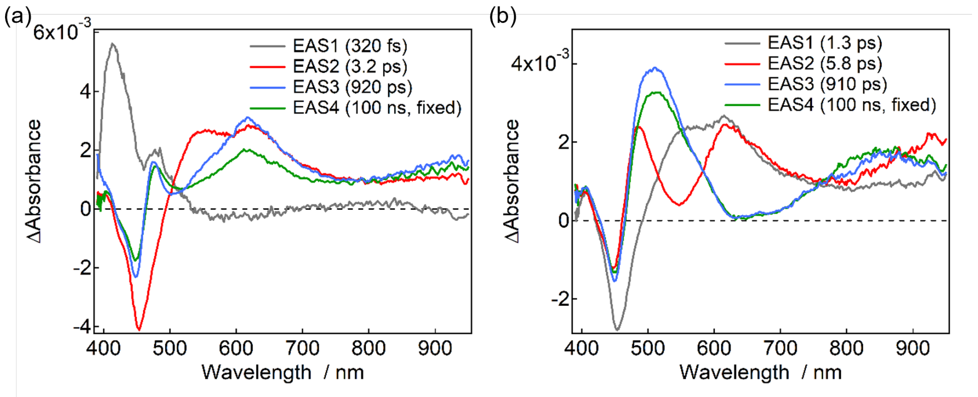

Figure S30. Evolution associated spectra (EAS) of the transient absorption spectra of **3** in (a) toluene (b) chloroform excited with a 347-nm pulse (36 nJ pulse^{-1}).

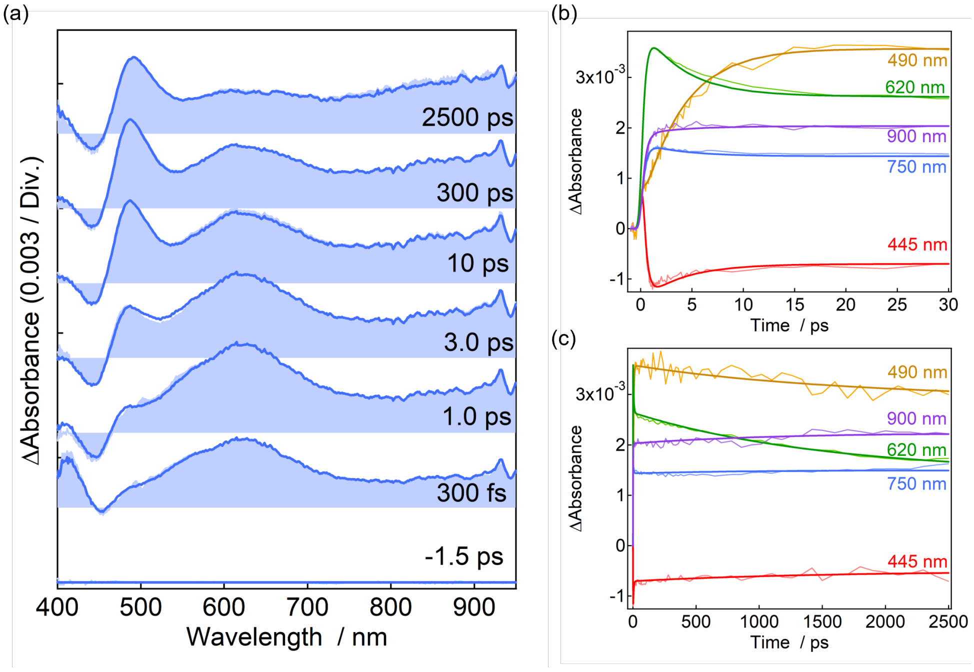

Figure S31. Time evolutions of femtosecond to nanosecond transient absorption spectra of **1** in toluene excited with a 347-nm pulse (36 nJ pulse^{-1}). Thick red, yellow, green, blue, and purple lines show the fitting lines by singular-value-decomposition (SVD) global analyses using a three-state sequential kinetic model.

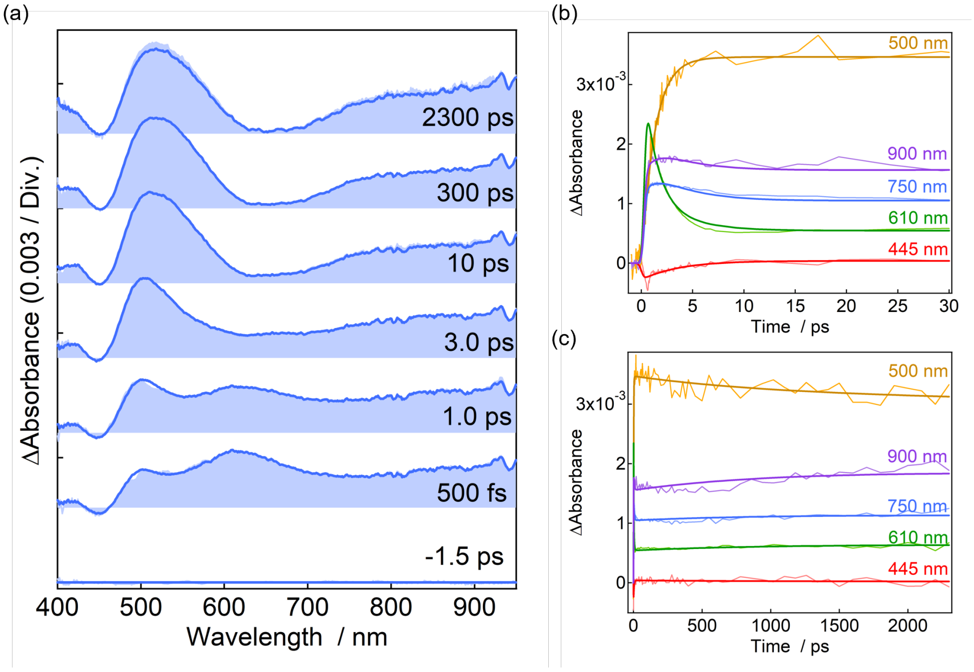

Figure S32. Time evolutions of femtosecond to nanosecond transient absorption spectra of **1** in chloroform excited with a 347-nm pulse (36 nJ pulse^{-1}). Thick red, yellow, green, blue, and purple lines show the fitting lines by SVD global analyses using a three-state sequential kinetic model.

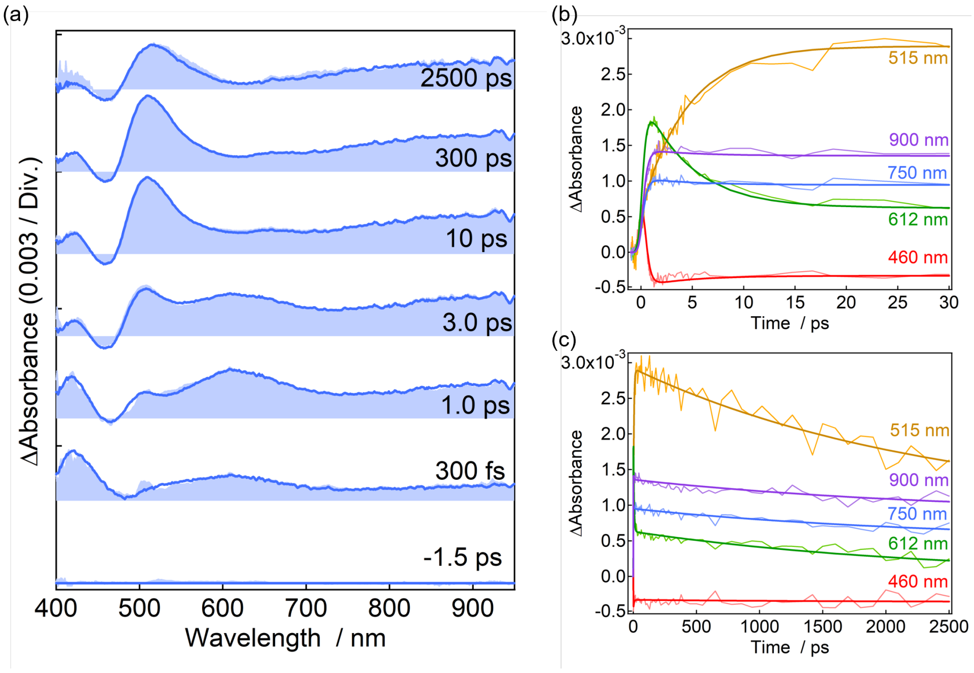

Figure S33. Time evolutions of femtosecond to nanosecond transient absorption spectra of **1** with TFMSA (1:235) in toluene excited with a 347-nm pulse (36 nJ pulse^{-1}). Thick red, yellow, green, blue, and purple lines show the fitting lines by SVD global analyses using a three-state sequential kinetic model.

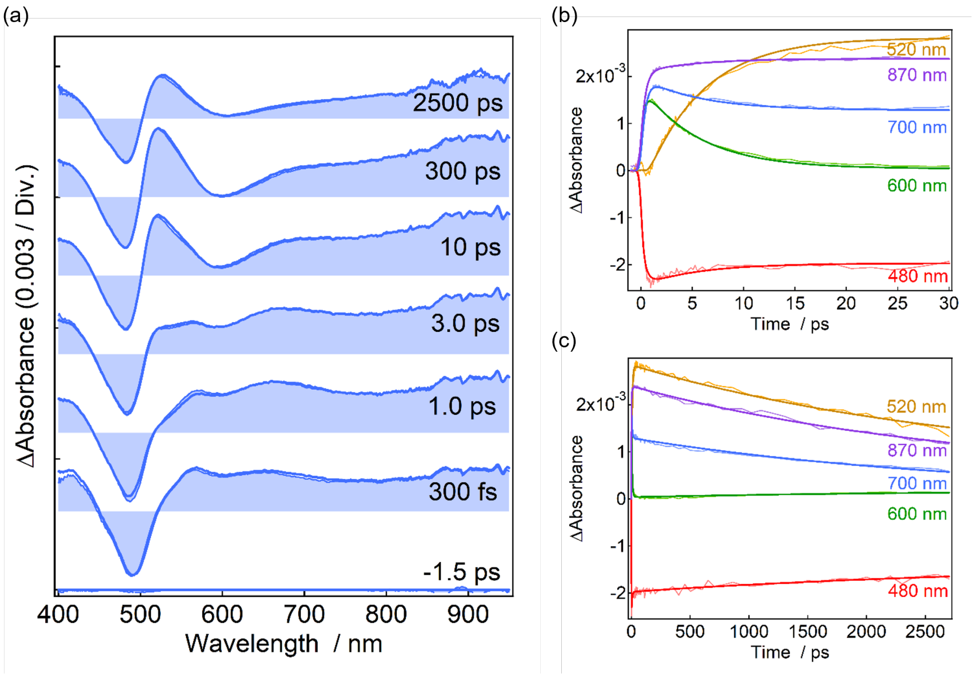

Figure S34. Time evolutions of femtosecond to nanosecond transient absorption spectra of **2** in toluene excited with a 347-nm pulse (36 nJ pulse^{-1}). Thick red, yellow, green, blue, and purple lines show the fitting lines by SVD global analyses using a three-state sequential kinetic model.

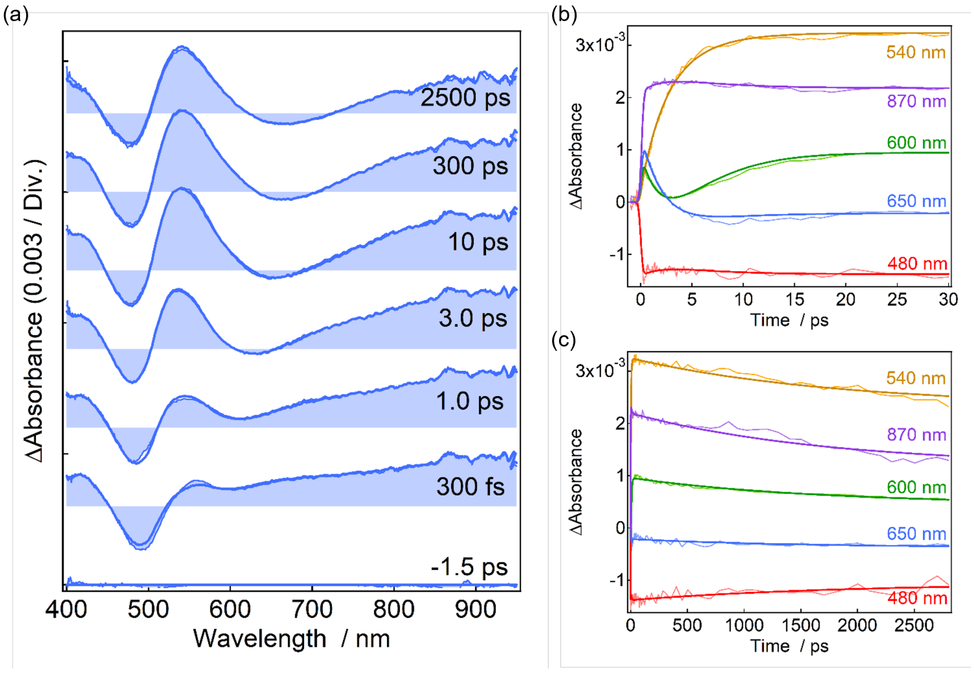

Figure S35. Time evolutions of femtosecond to nanosecond transient absorption spectra of **2** in chloroform excited with a 347-nm pulse (36 nJ pulse^{-1}). Thick red, yellow, green, blue, and purple lines show the fitting lines by SVD global analyses using a three-state sequential kinetic model.

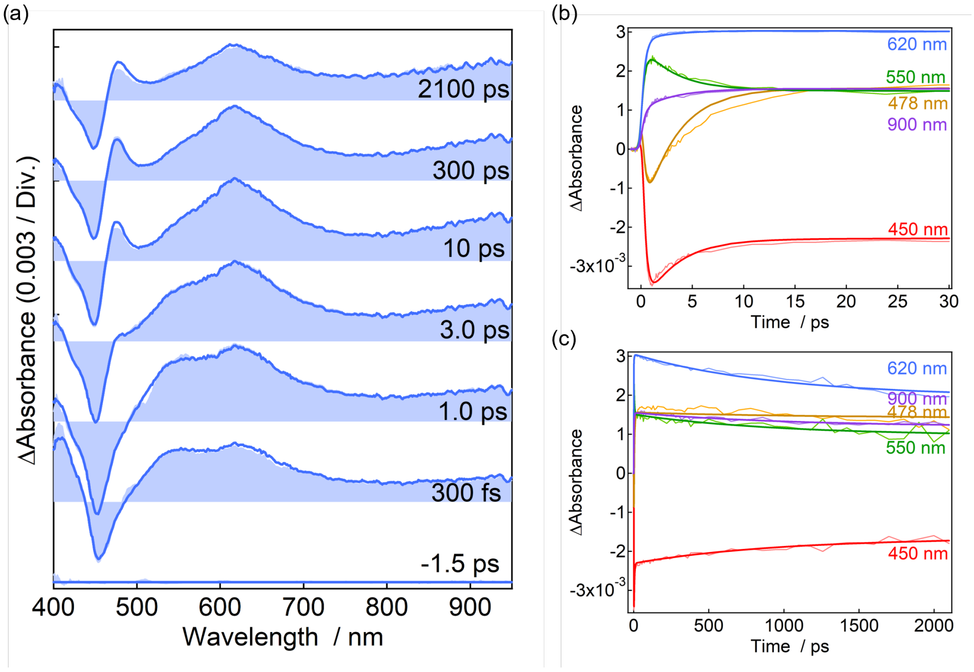

Figure S36. Time evolutions of femtosecond to nanosecond transient absorption spectra of **3** in toluene excited with a 347-nm pulse (36 nJ pulse^{-1}). Thick red, yellow, green, blue, and purple lines show the fitting lines by SVD global analyses using a three-state sequential kinetic model.

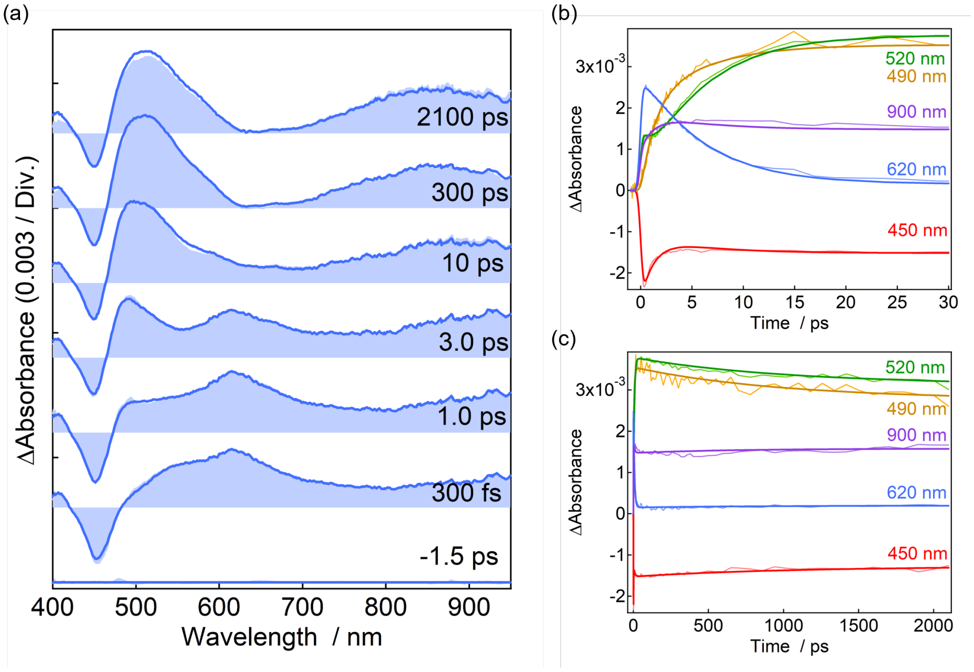

Figure S37. Time evolutions of femtosecond to nanosecond transient absorption spectra of **3** in chloroform excited with a 347-nm pulse (36 nJ pulse^{-1}). Thick red, yellow, green, blue, and purple lines show the fitting lines by SVD global analyses using a three-state sequential kinetic model.

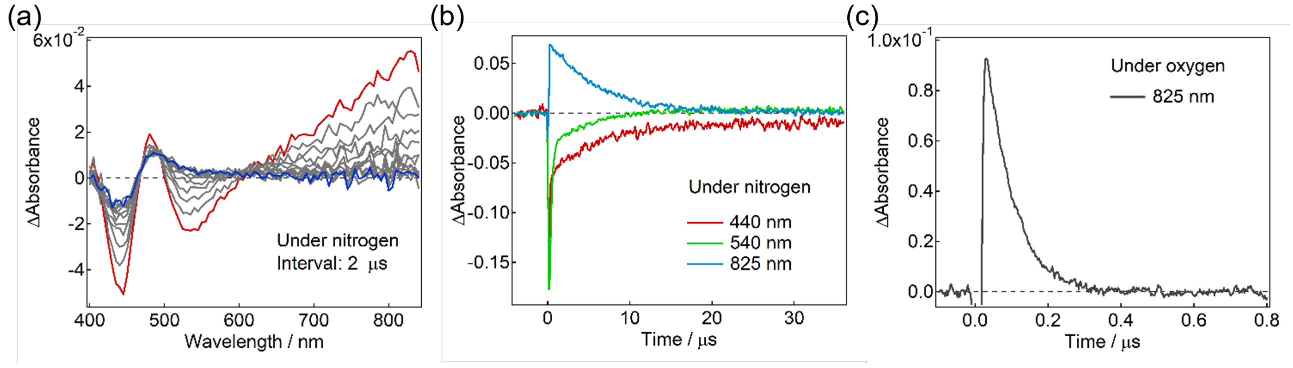

Figure S38. Microsecond transient absorption (a) spectra and (b) decays under nitrogen and (c) under oxygen of **1** in toluene excited with a 355-nm pulse (1.0 mJ pulse^{-1}).

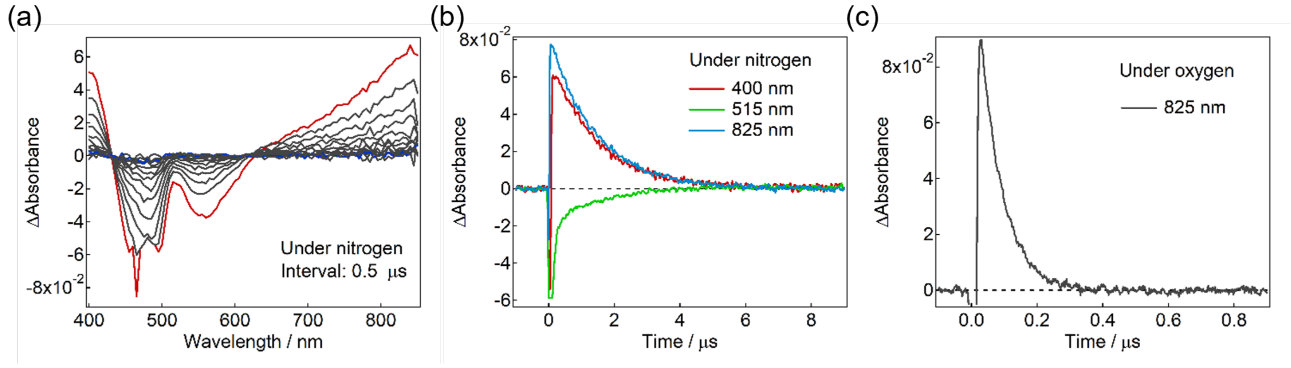

Figure S39. Microsecond transient absorption (a) spectra and (b) decays under nitrogen, and (c) under oxygen of **2** in toluene excited with a 355-nm pulse (1.0 mJ pulse^{-1}).

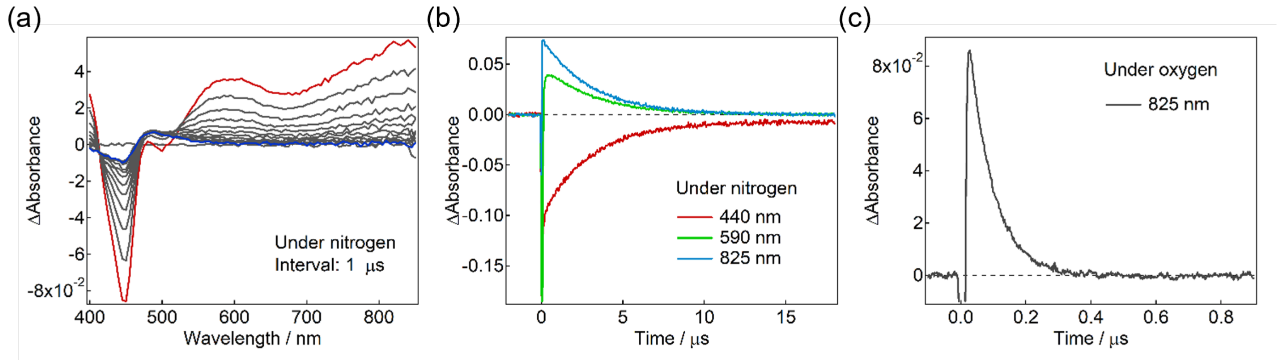

Figure S40. Microsecond transient absorption (a) spectra and (b) decays under nitrogen and (c) under oxygen of **3** in toluene excited with a 355-nm pulse (1.0 mJ pulse^{-1}).

Theoretical Calculations

Density Functional Theory (DFT) calculations were performed using Gaussian 16 software to optimize molecular geometries and conduct frequency analyses at the ground (S_0) and the first excited singlet state (S_1). Tamm-Dancoff (TDA) approximation was used. It employed the PBE0 functional paired with the 6-31+G(d,p) basis set. Frequency calculations were conducted on optimized geometries to ensure minima (absence of imaginary frequencies) and confirm the stability of these structures.

The range-separation parameter (ω) for the long-range corrected hybrid functional LRC-ωPBEh was non-empirically tuned for **1** using the optimal tuning protocol implemented in QChem 5.0 software. This protocol involves systematically adjusting ω to minimize deviations from the Koopmans' theorem, specifically ensuring that the energy of the highest occupied molecular orbital (HOMO) closely matches the negative ionization potential calculated by energy differences. The optimized ω parameter resulting from this tuning procedure was 0.124 $bohr^{-1}$, and this value was consistently used across all TD-DFT calculations.

To quantify hydrogen-bond interaction energies ($E_{H\text{-bond}}$) in the ground state, total energy calculations were performed on the hydrogen-bonded complex **1+TFMSA** and its isolated fragments **1** and **TFMSA**, following the supermolecular approach:

$E_{H\text{-bond}}(S_0) = E_{\mathbf{1+TFMSA}} - E_{\mathbf{1}} - E_{\mathbf{TFMSA}}$

where $E_{\mathbf{1+TFMSA}}$ is the total energy of the complex, $E_{\mathbf{1}}$ and $E_{\mathbf{TFMSA}}$ are the energies of the isolated fragments in their relaxed geometries.

For the excited-state hydrogen-bond strength $E_{H\text{-bond}}(S_1)$, the protocol was modified to maintain balanced excitation within the complex. Specifically, the reference fragment (here the chromophore **1**) is modeled in its S_1 optimized geometry and electronic state, while the attached fragment (here **TFMSA**) remains in its S_0 ground state. The binding energy is then computed as:

$E_{H\text{-bond}}(S_1) = E^*_{\mathbf{1+TFMSA}} - E^*_{\mathbf{1}} - E_{\mathbf{TFMSA}}$

Here, $E^*_{\mathbf{1+TFMSA}}$ is the energy of the complex in the S1 state, $E^*_{\mathbf{1}}$ is the energy of the isolated **1** in the S_1 state, and $E_{\mathbf{TFMSA}}$ is the energy of the isolated TFMSA in its S_0 state. For the methodological consistency of comparing ground- and excited-state total energies, the ground-state energies were calculated with PBE0 while only the excitation energy was calculated with the range-separated functional.

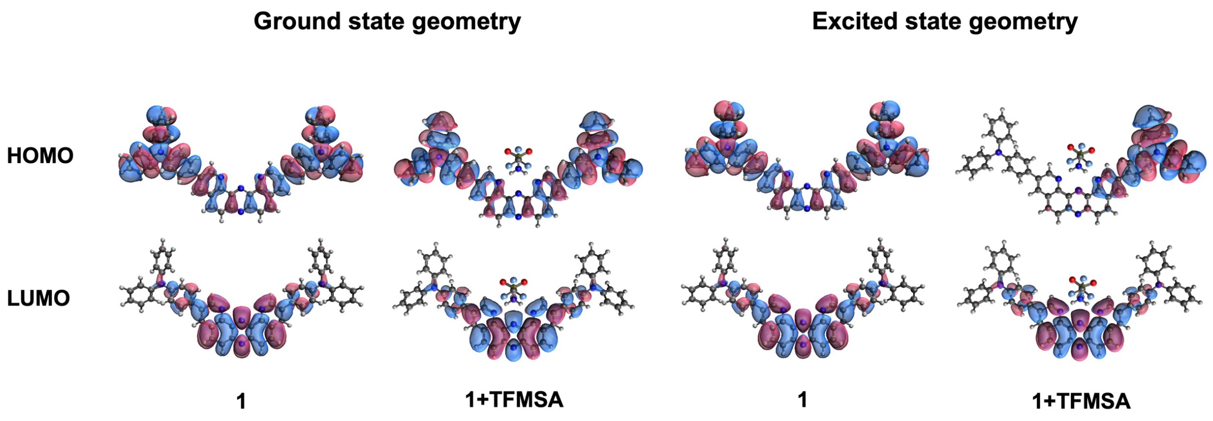

Figure S41. HOMO and LUMO orbitals of **1** and **1+TFMSA** at ground and excited state geometries.

Table S5. Frontier orbital energy levels of **1** and **1+TFMSA** calculated with PBE0 functional.

	**Ground state geometry**		**Excited state geometry**	
	**1**	**1+TFMSA**	**1**	**1+TFMSA**
**HOMO**	–0.20184 a.u.	–0.20446 a.u.	–0.19915 a.u.	–0.19901 a.u.
**LUMO**	–0.08617 a.u.	–0.10224 a.u.	–0.09412 a.u.	–0.11251 a.u.

Photophysical properties were studied with the Nuclear Ensemble Method by using NEMO software. This method systematically considers vibrational motions and their influence on optical properties by generating an ensemble of molecular conformations to represent vibrational distributions. Nuclear ensembles comprising 500 distinct molecular conformations each were independently generated for the ground and excited states. Subsequent ensemble-based time-dependent DFT(TD-DFT) calculations were carried out with QChem 5.4, employing the tuned LRC-ωPBEh functional and 6-31+G(d,p) basis set, explicitly including toluene as the solvent to model realistic experimental conditions. Here the red shift was observed in both absorption and emission spectra of **1** with respect to the complex.

Table S6. Intersystem crossing rate constants for the transitions from the S1 state. The rate constants are calculated using the Nuclear Ensemble Method.

**Transition**	k_{ISC} **(1)**	k_{ISC} **(1+TFMSA)**
$S1 \rightsquigarrow T1$	$3 \times 10^{-51}\ s^{-1}$	$2 \times 10^{5}\ s^{-1}$
$S1 \rightsquigarrow T2$	$1 \times 10^{6}\ s^{-1}$	$2.2 \times 10^{8}\ s^{-1}$
$S1 \rightsquigarrow T3$	$5 \times 10^{8}\ s^{-1}$	$9 \times 10^{8}\ s^{-1}$

$S1 \rightsquigarrow T4$	$6 \times 10^{8}\ s^{-1}$	$9 \times 10^{8}\ s^{-1}$
$S1 \rightsquigarrow T5$	$1.8 \times 10^{9}\ s^{-1}$	$7 \times 10^{8}\ s^{-1}$

Natural Bond Orbital (NBO) analyses were conducted to examine bonding interactions, electron delocalization, and donor-acceptor characteristics of optimized ground-state (S_0) structures. These analyses utilized QChem software at the PBE0/6-31+G(d,p) level. Additionally, Natural Transition Orbital (NTO) analyses were conducted for the first excited state (S_1) complexes using QChem software at the tuned LRC-ωPBEh/6-31+G(d,p) level. Visualization and interpretation of the NBO and NTO analyses were performed with IQmol software, facilitating clear representation and analysis of electronic distributions and transitions.

Copies of NMR Charts of New Compounds

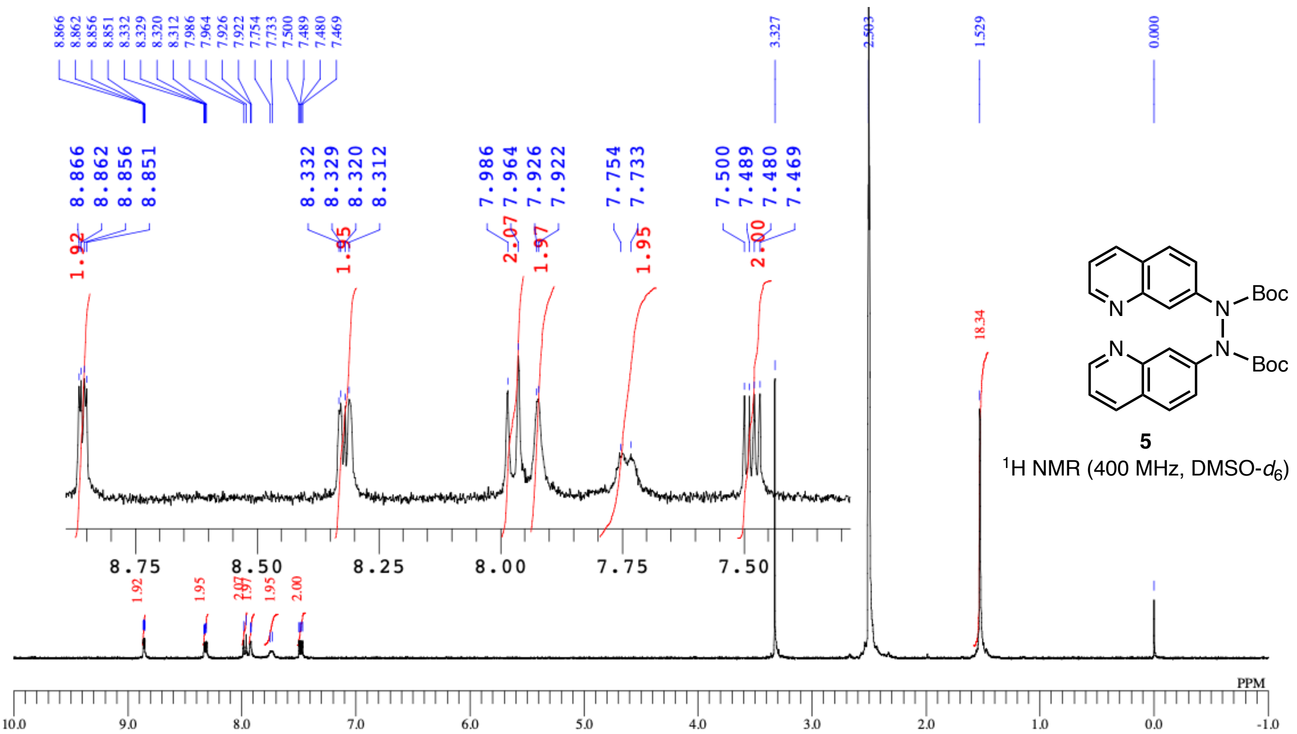

5

^{1}H NMR (400 MHz, DMSO-d_6)

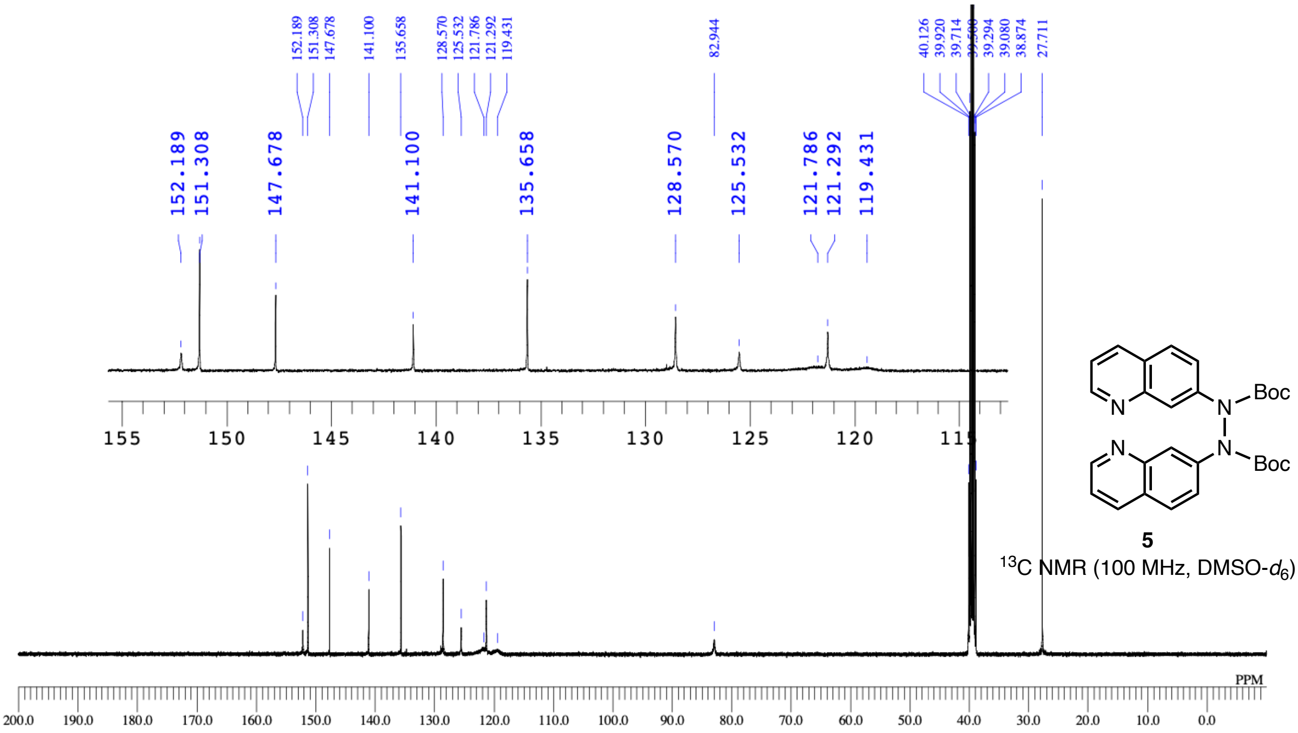

5

^{13}C NMR (100 MHz, DMSO-d_6)

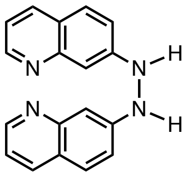

6

^{1}H NMR (400 MHz, $CDCl_3$)

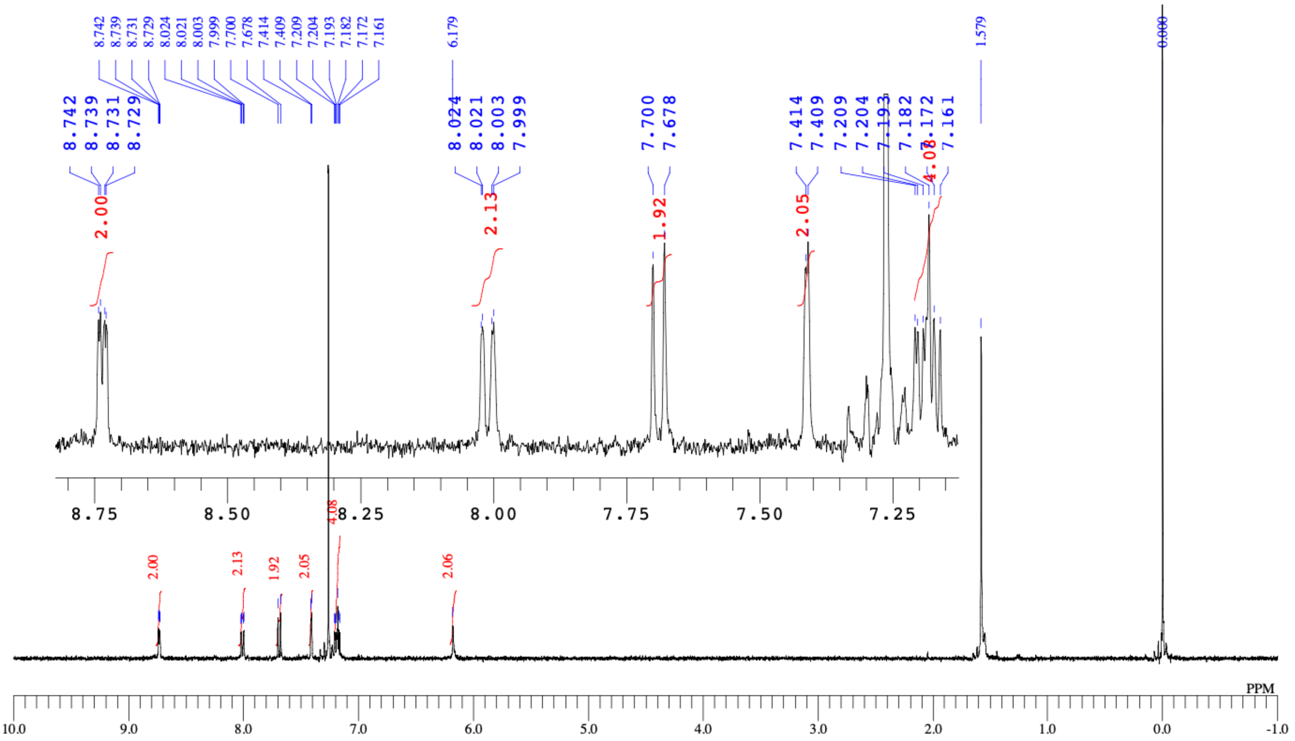

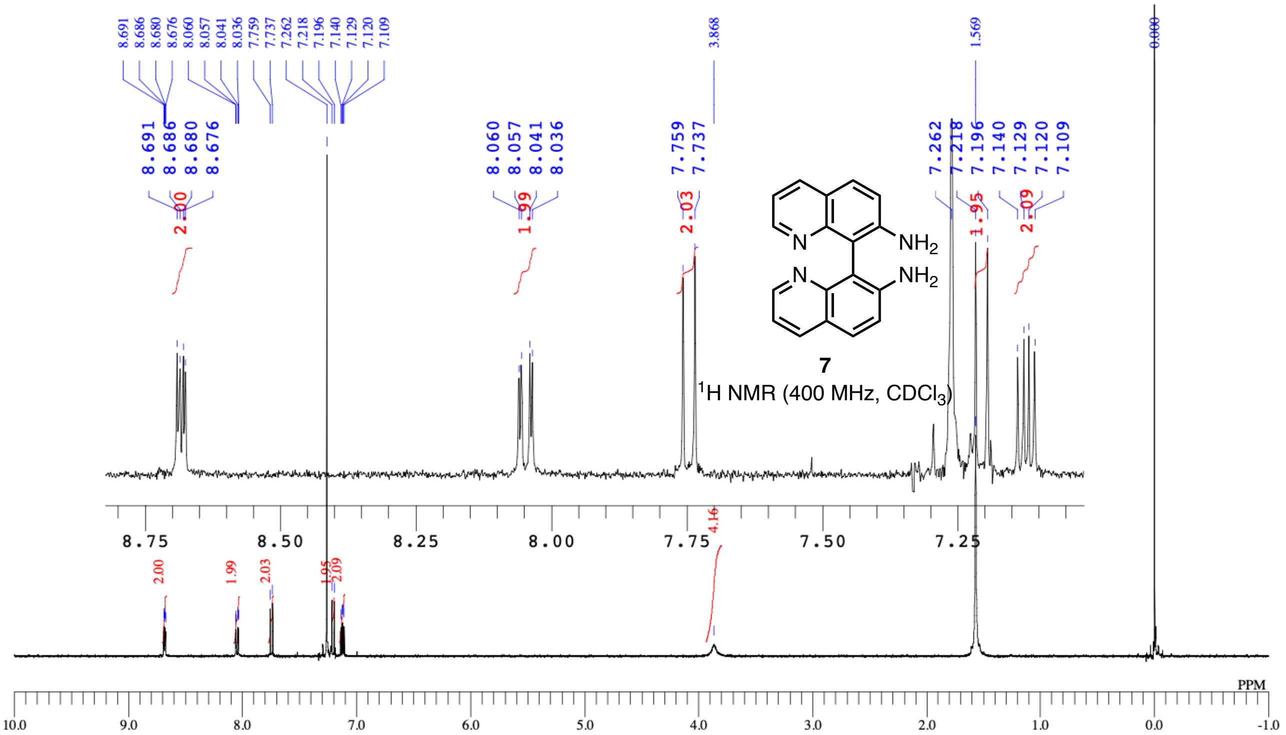
7
¹H NMR (400 MHz, CDCl3)

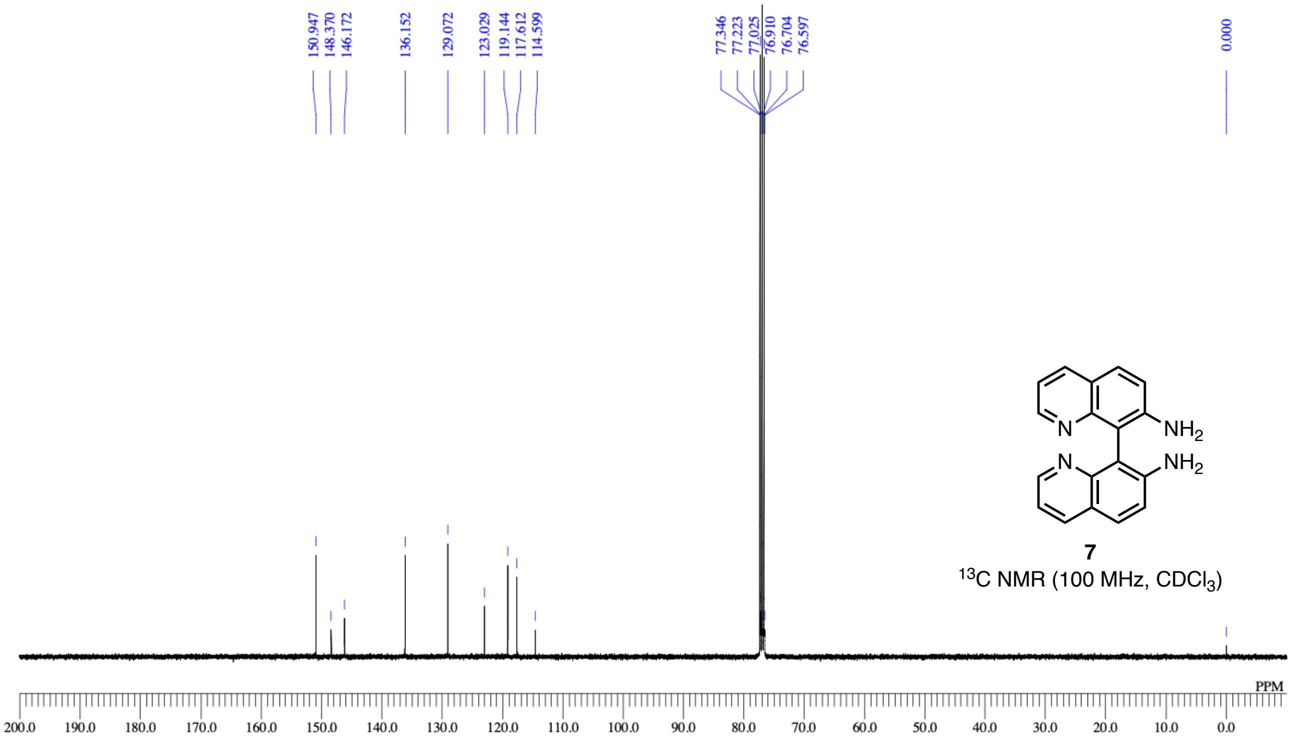
7
¹³C NMR (100 MHz, CDCl3)

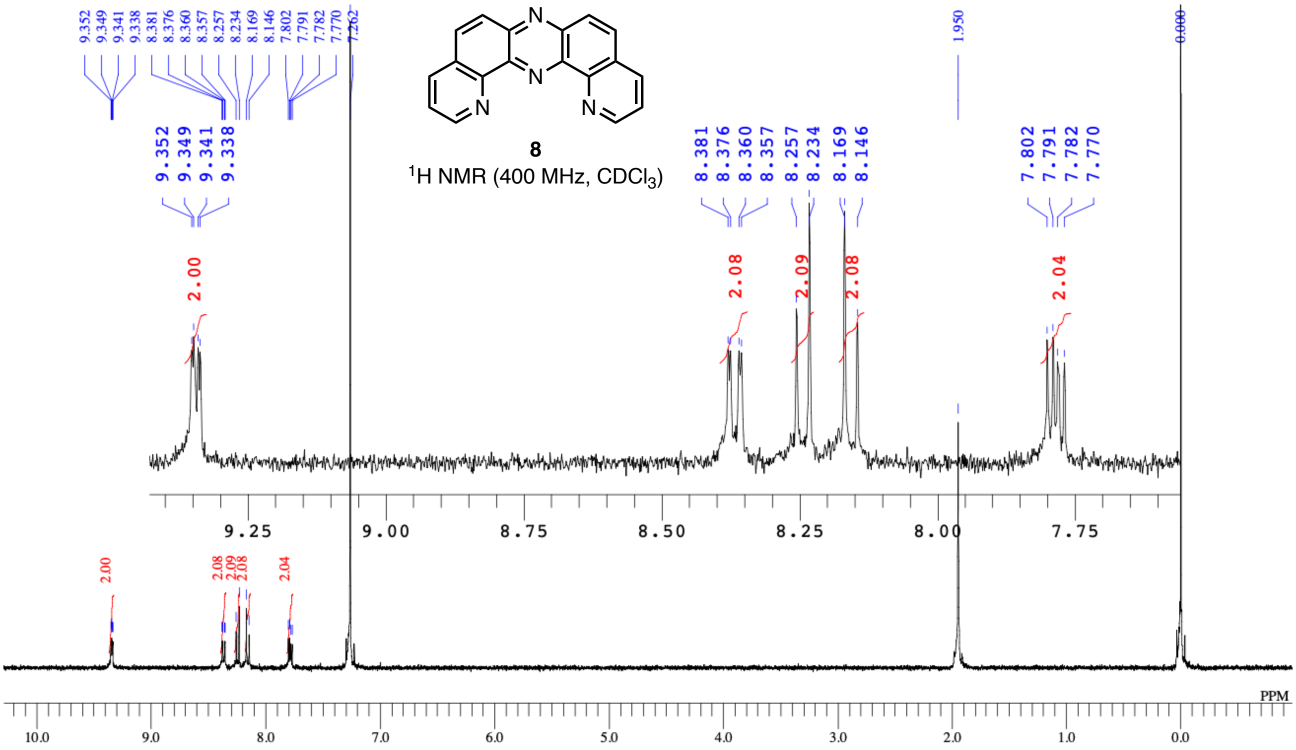
9.352 9.349 9.341 9.338 8.381 8.376 8.360 8.357 8.257 8.234 8.169 8.146 7.802 7.791 7.782 7.770 7.262
1.950
0.000
8
1H NMR (400 MHz, CDCl3)
2.00 2.08 2.09 2.08 2.04
9.25 9.00 8.75 8.50 8.25 8.00 7.75
PPM
10.0 9.0 8.0 7.0 6.0 5.0 4.0 3.0 2.0 1.0 0.0

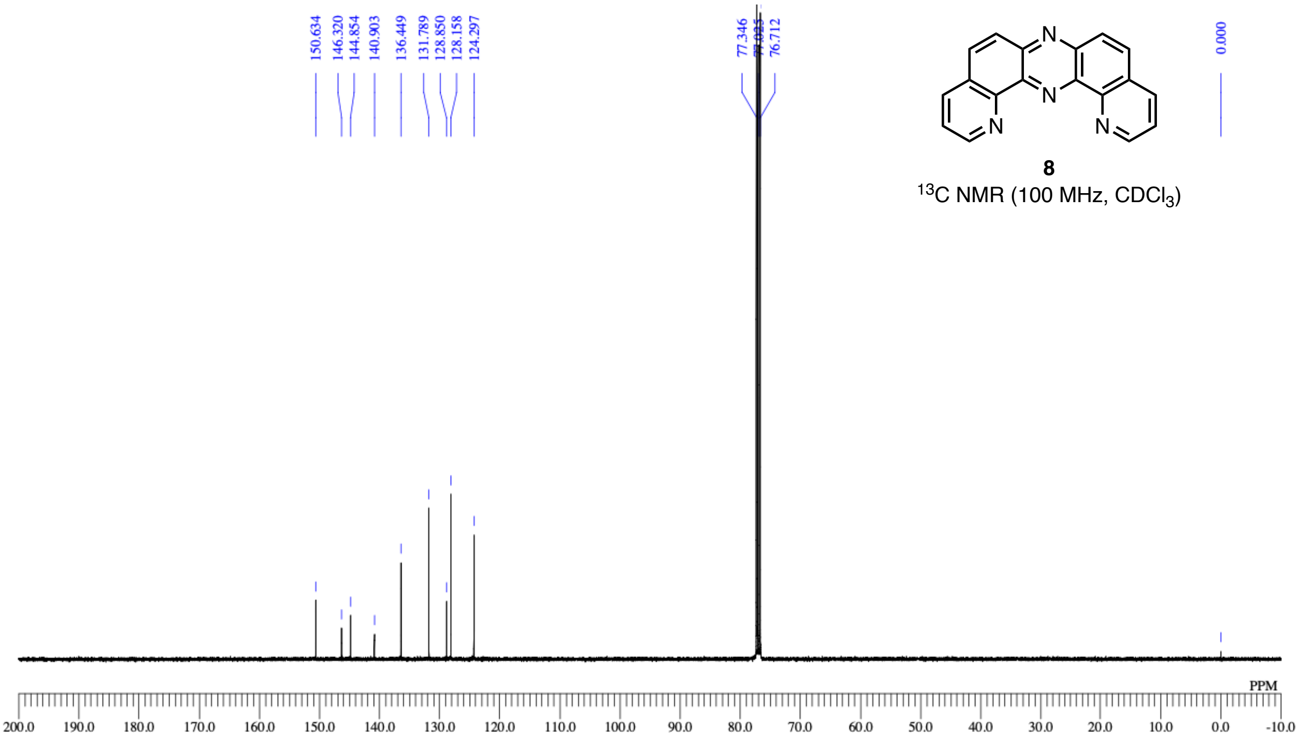
150.634 146.320 144.854 140.903 136.449 131.789 128.850 128.158 124.297
77.346 76.712
0.000
8
13C NMR (100 MHz, CDCl3)
PPM
200.0 190.0 180.0 170.0 160.0 150.0 140.0 130.0 120.0 110.0 100.0 90.0 80.0 70.0 60.0 50.0 40.0 30.0 20.0 10.0 0.0 -10.0

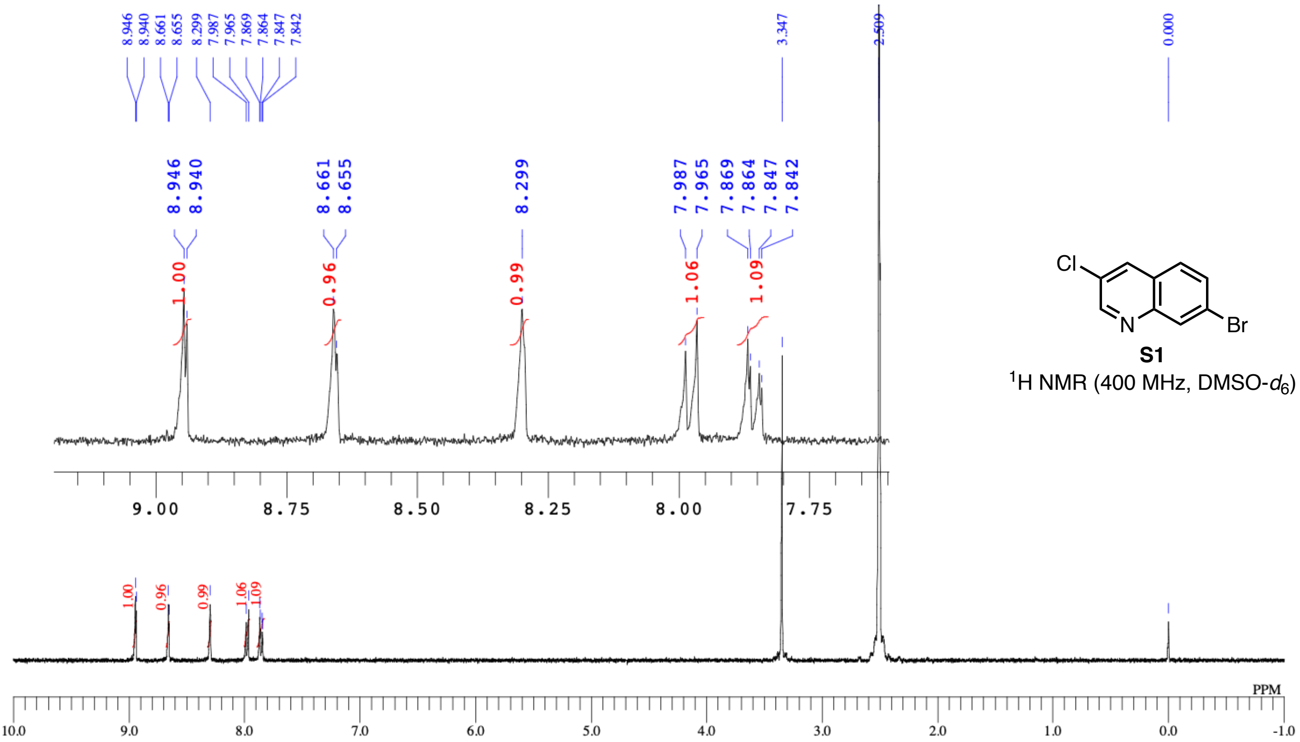

^{1}H NMR (400 MHz, DMSO-d_6)

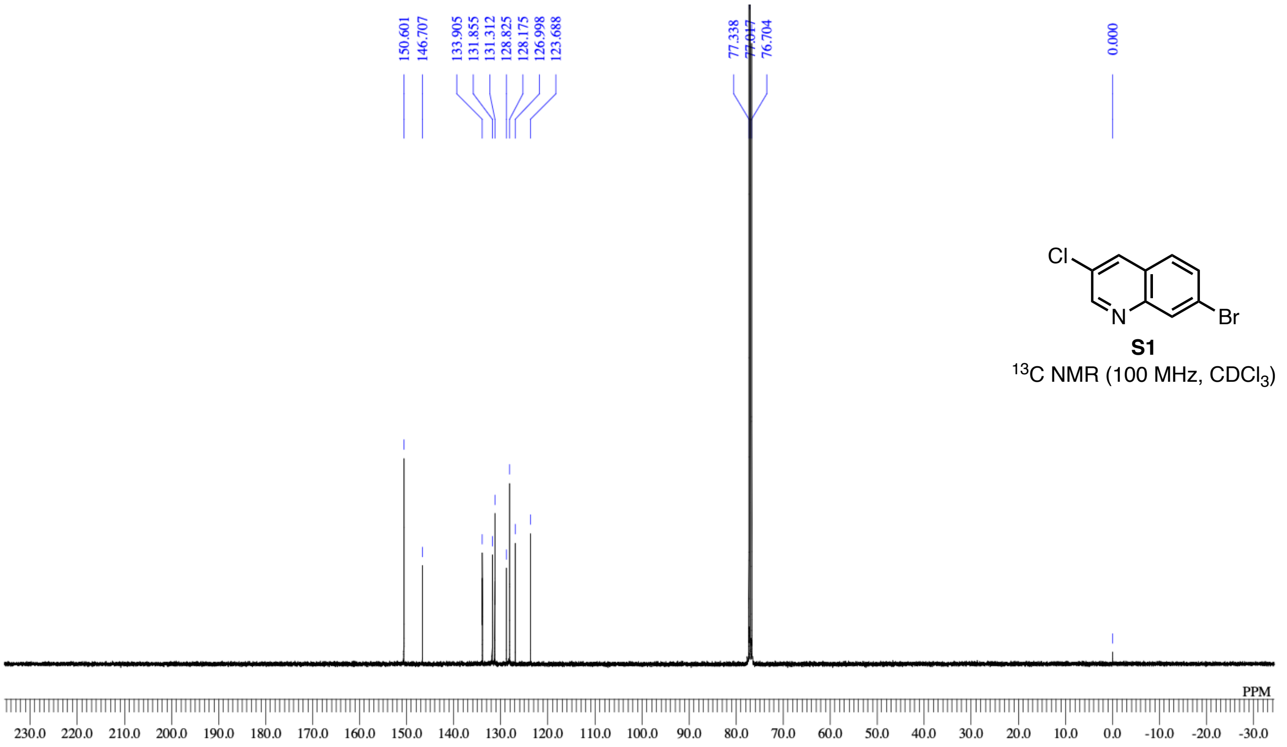

^{13}C NMR (100 MHz, $CDCl_3$)

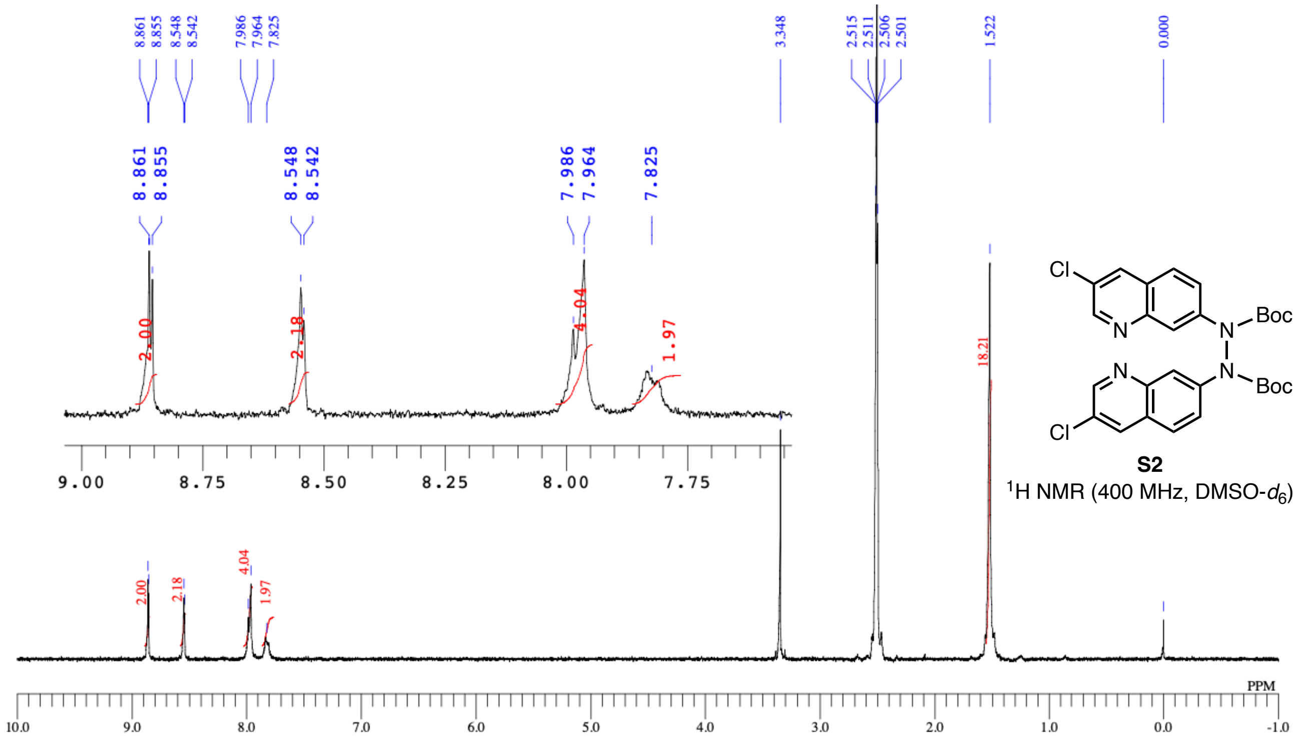

S2

^{1}H NMR (400 MHz, DMSO-d_6)

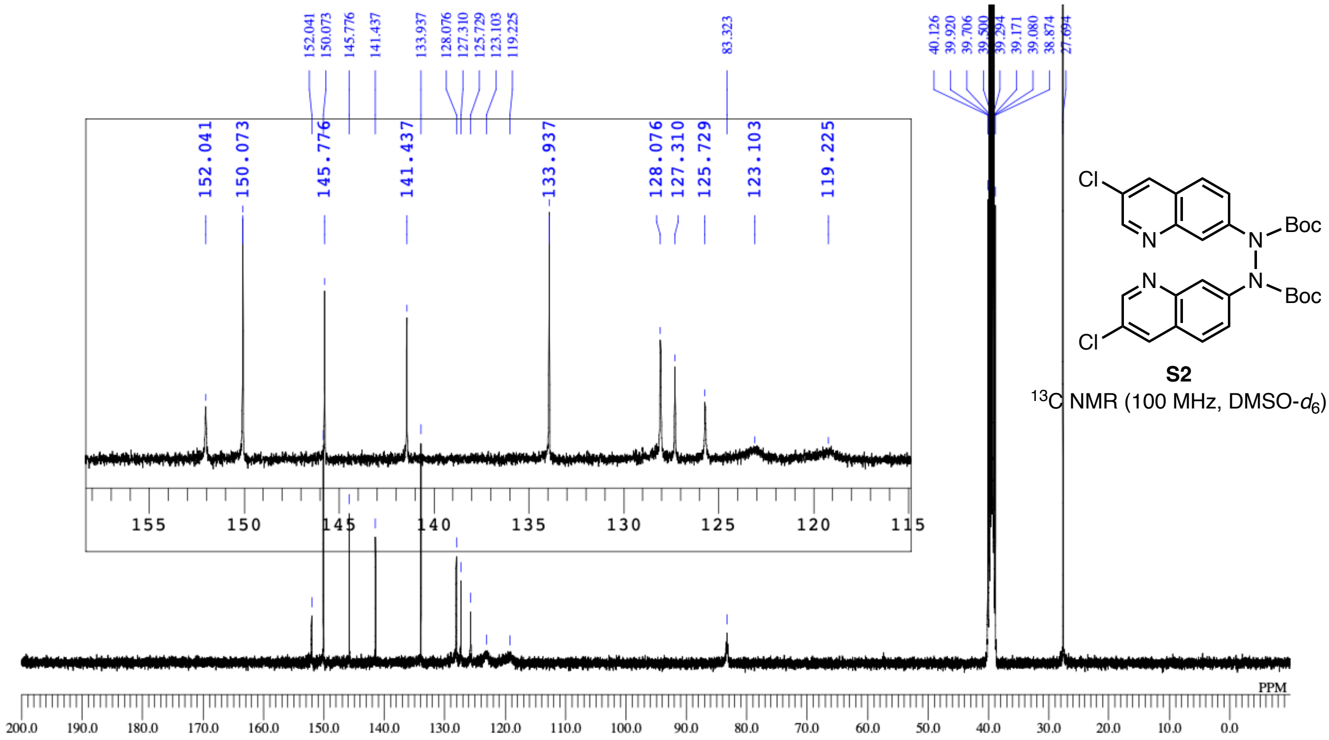

S2

^{13}C NMR (100 MHz, DMSO-d_6)

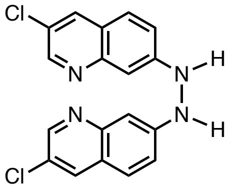

S3

^{1}H NMR (400 MHz, $CDCl_3$)

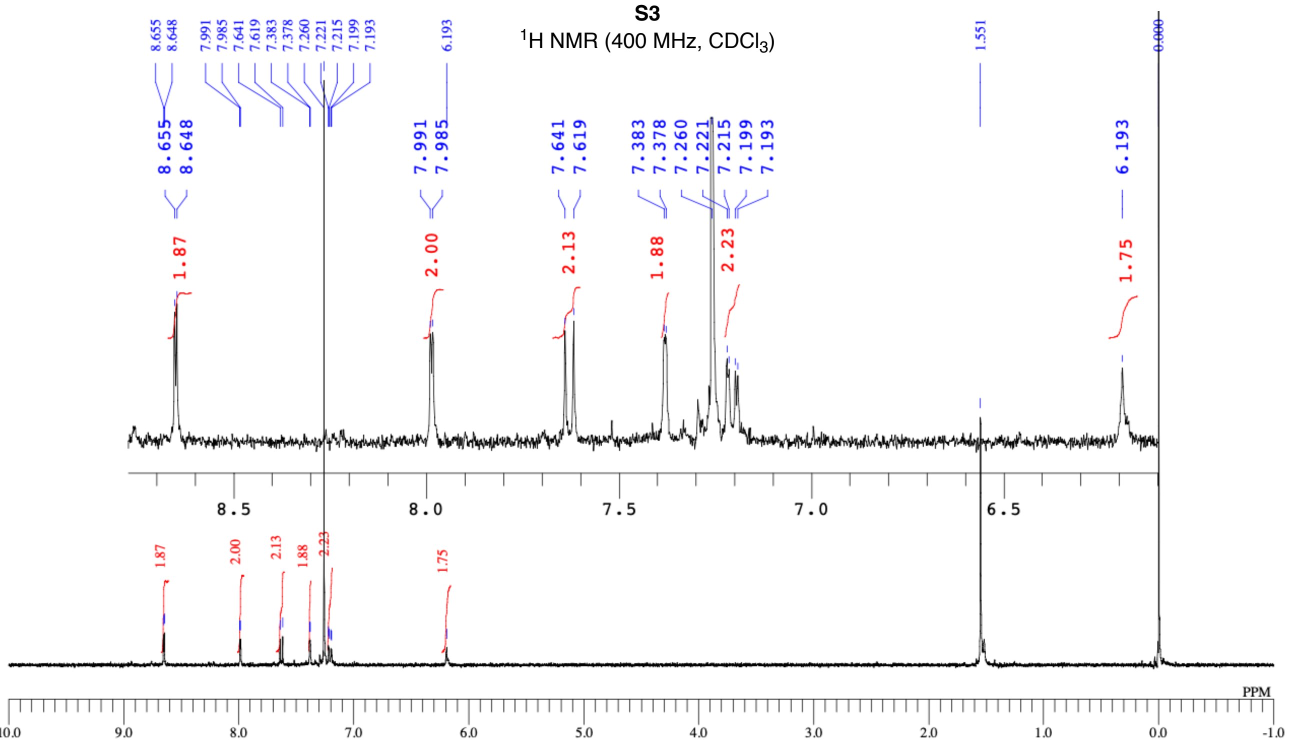

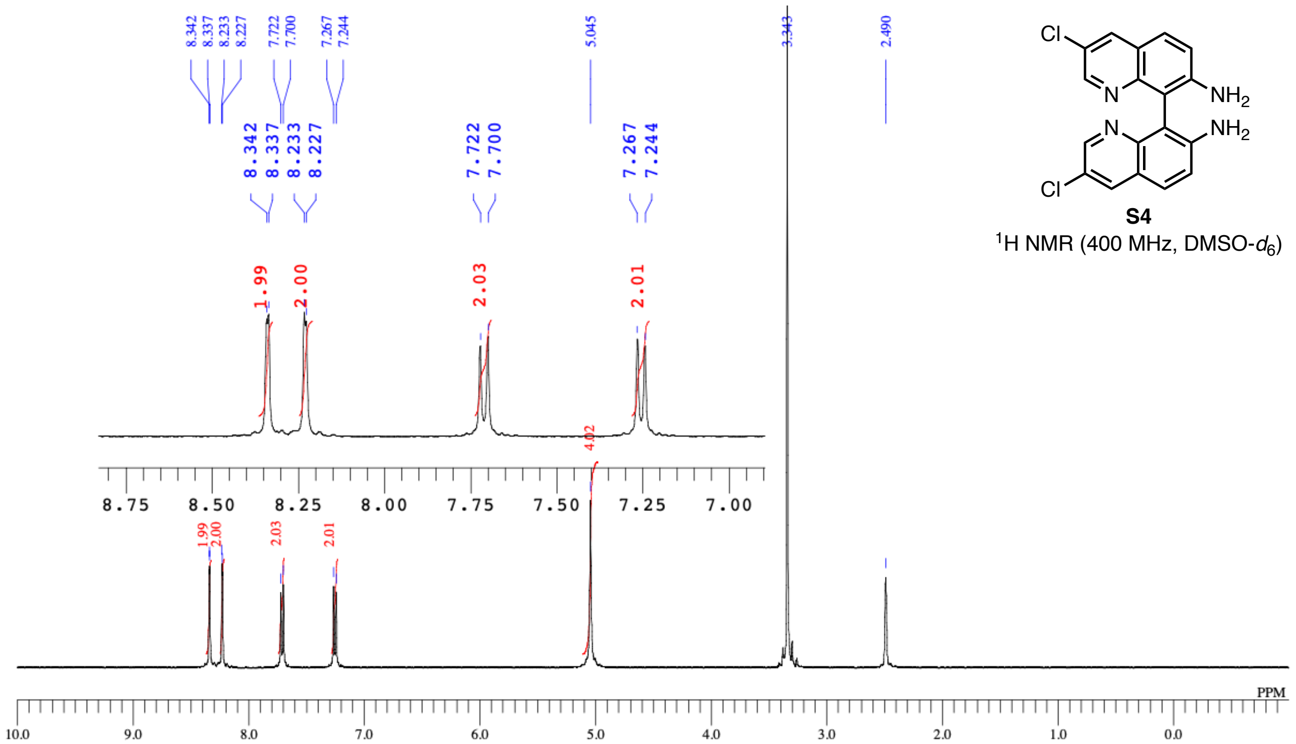
Cl
N
NH2
N
NH2
Cl
S4
1H NMR (400 MHz, DMSO-d6)
8.342
8.337
8.233
8.227
7.722
7.700
7.267
7.244
5.045
3.343
2.490
1.99
2.00
2.03
2.01
4.02
PPM

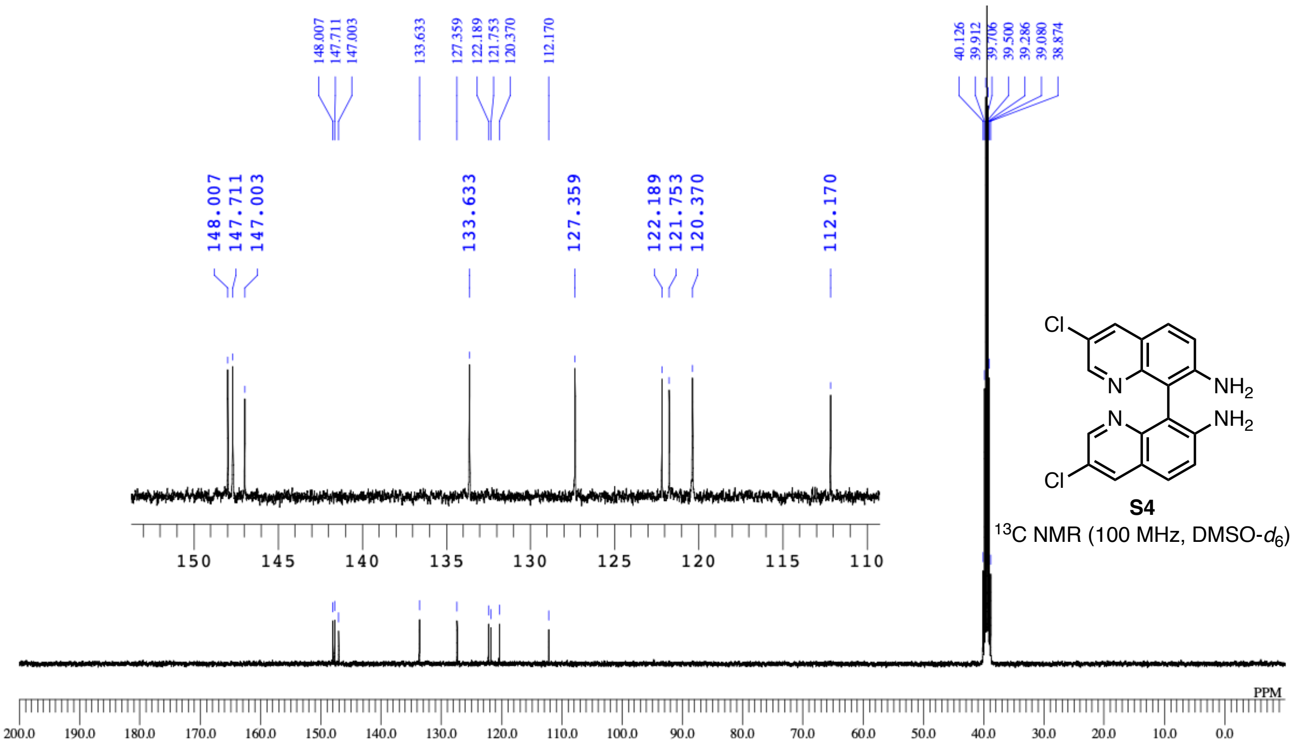
Cl
N
NH2
N
NH2
Cl
S4
13C NMR (100 MHz, DMSO-d6)
148.007
147.711
147.003
133.633
127.359
122.189
121.753
120.370
112.170
40.126
39.912
39.706
39.500
39.286
39.080
38.874
PPM

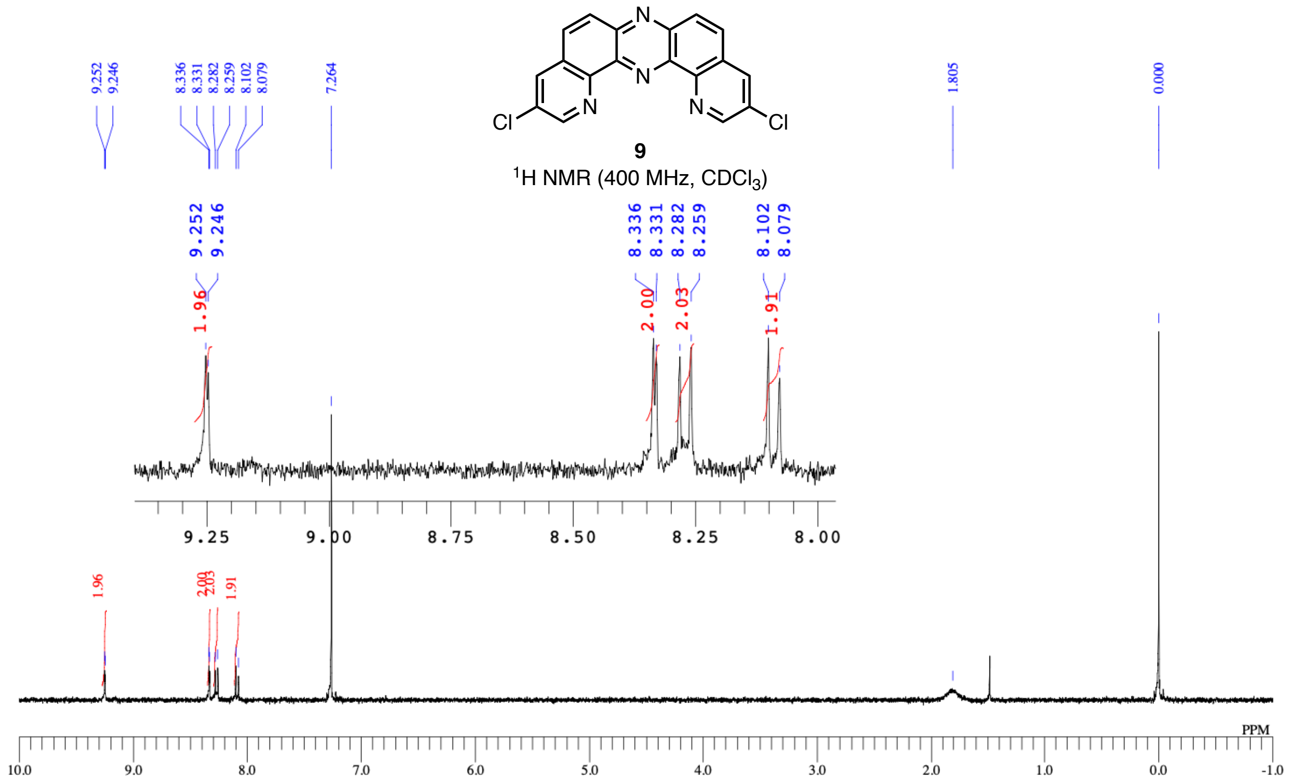
9
^{1}H NMR (400 MHz, $CDCl_3$)
9.252 9.246 8.336 8.331 8.282 8.259 8.102 8.079 7.264 1.805 0.000
1.96 2.00 2.03 1.91
9.25 9.00 8.75 8.50 8.25 8.00
PPM
10.0 9.0 8.0 7.0 6.0 5.0 4.0 3.0 2.0 1.0 0.0 -1.0

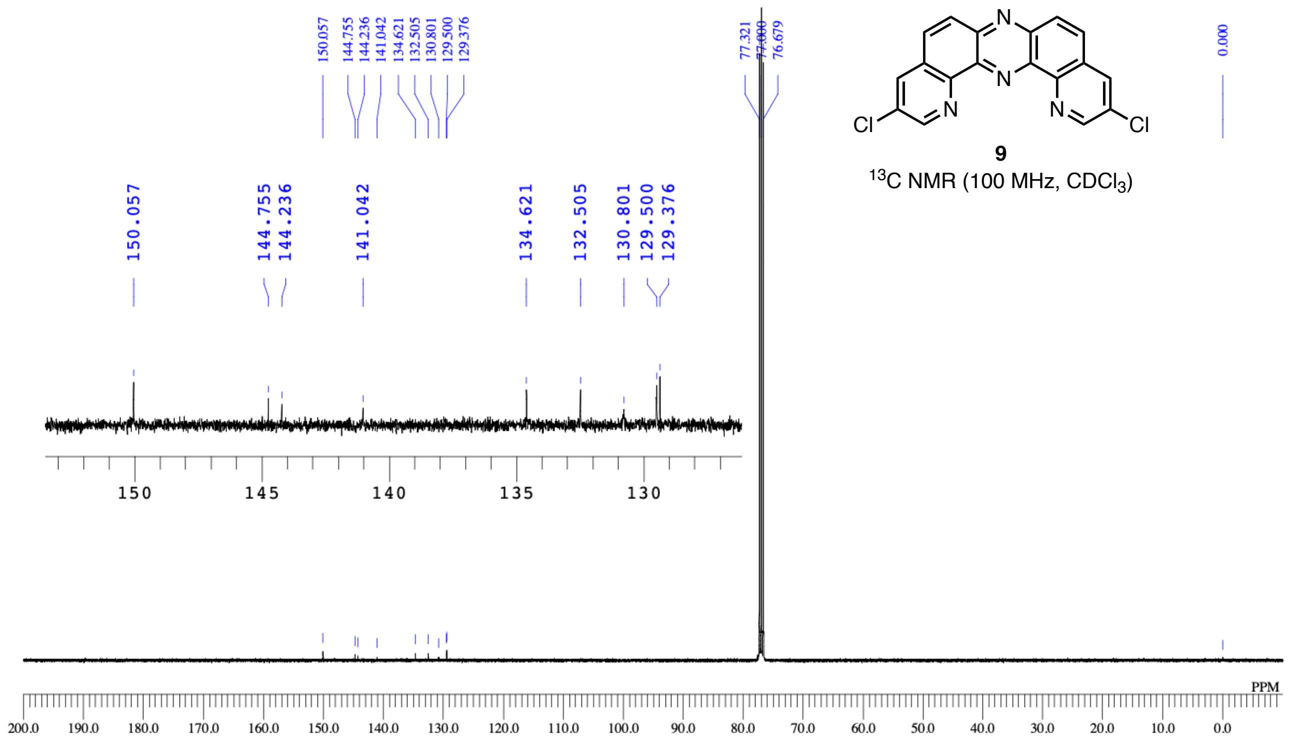
9
^{13}C NMR (100 MHz, $CDCl_3$)
150.057 144.755 144.236 141.042 134.621 132.505 130.801 129.500 129.376
77.321 77.000 76.679 0.000
150 145 140 135 130
PPM
200.0 190.0 180.0 170.0 160.0 150.0 140.0 130.0 120.0 110.0 100.0 90.0 80.0 70.0 60.0 50.0 40.0 30.0 20.0 10.0 0.0

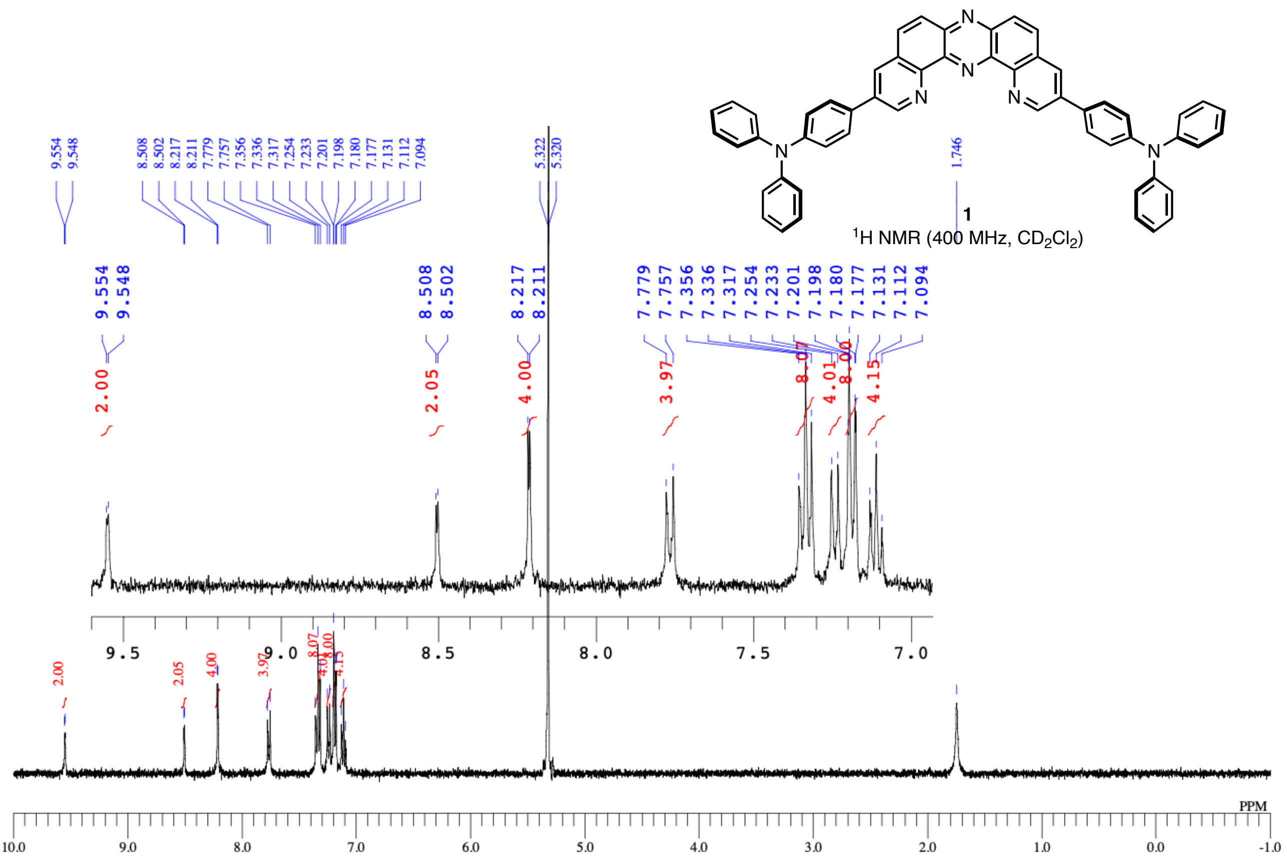
1
^{1}H NMR (400 MHz, CD_2Cl_2)

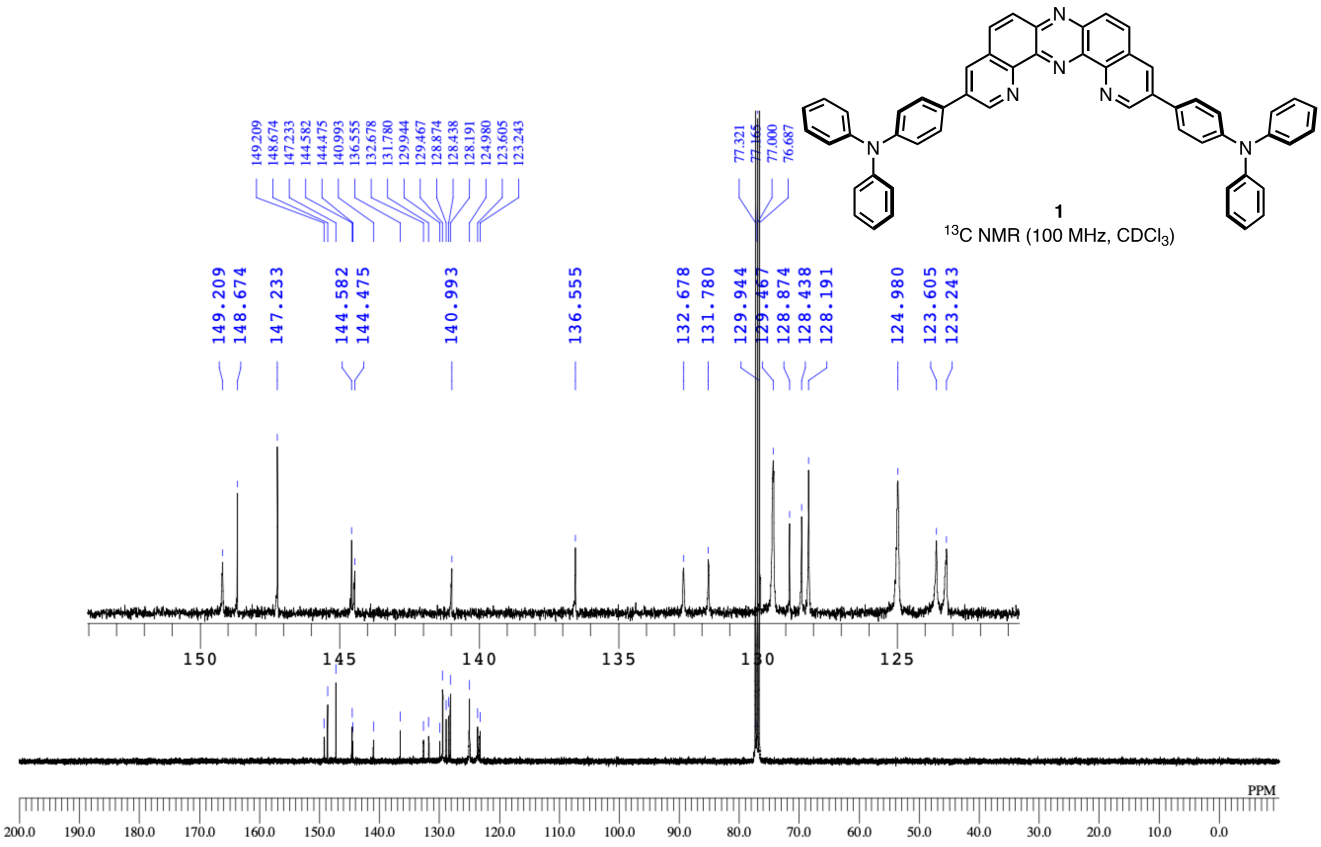
1
^{13}C NMR (100 MHz, $CDCl_3$)

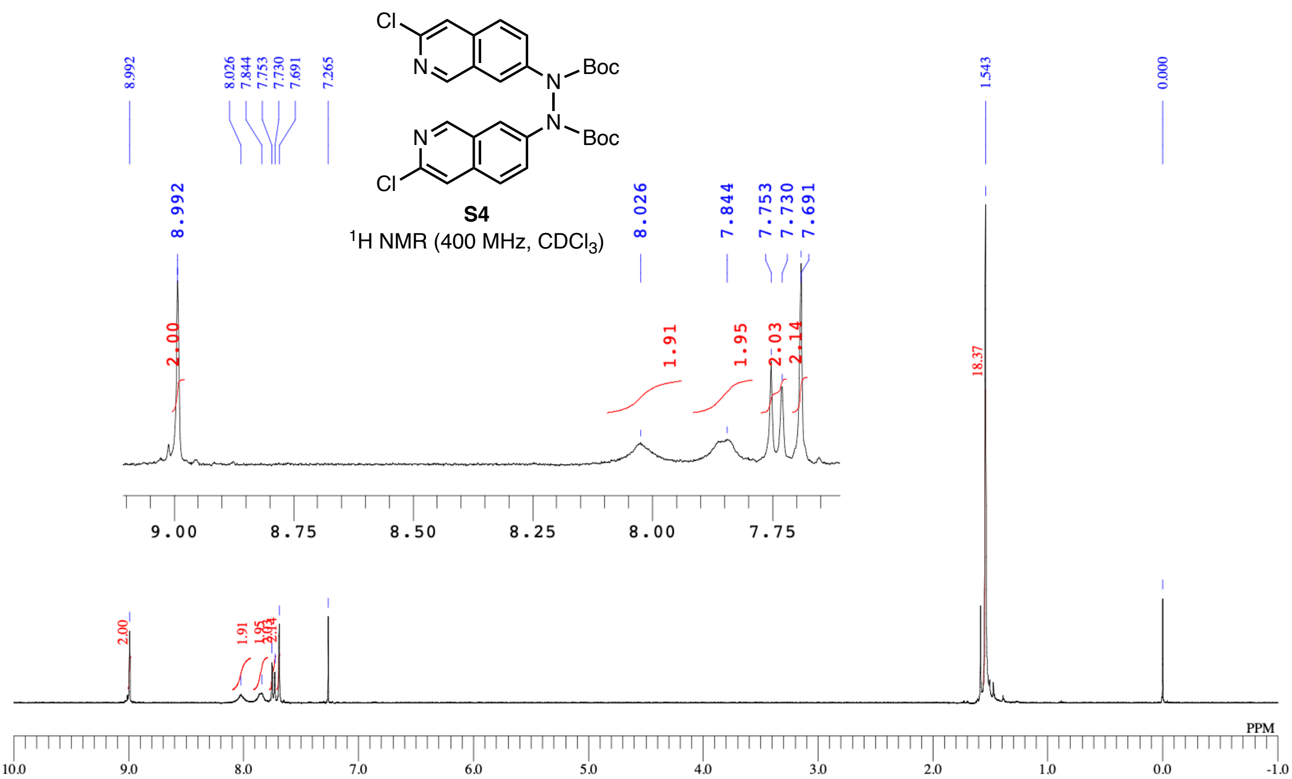
S4
1H NMR (400 MHz, CDCl3)
PPM

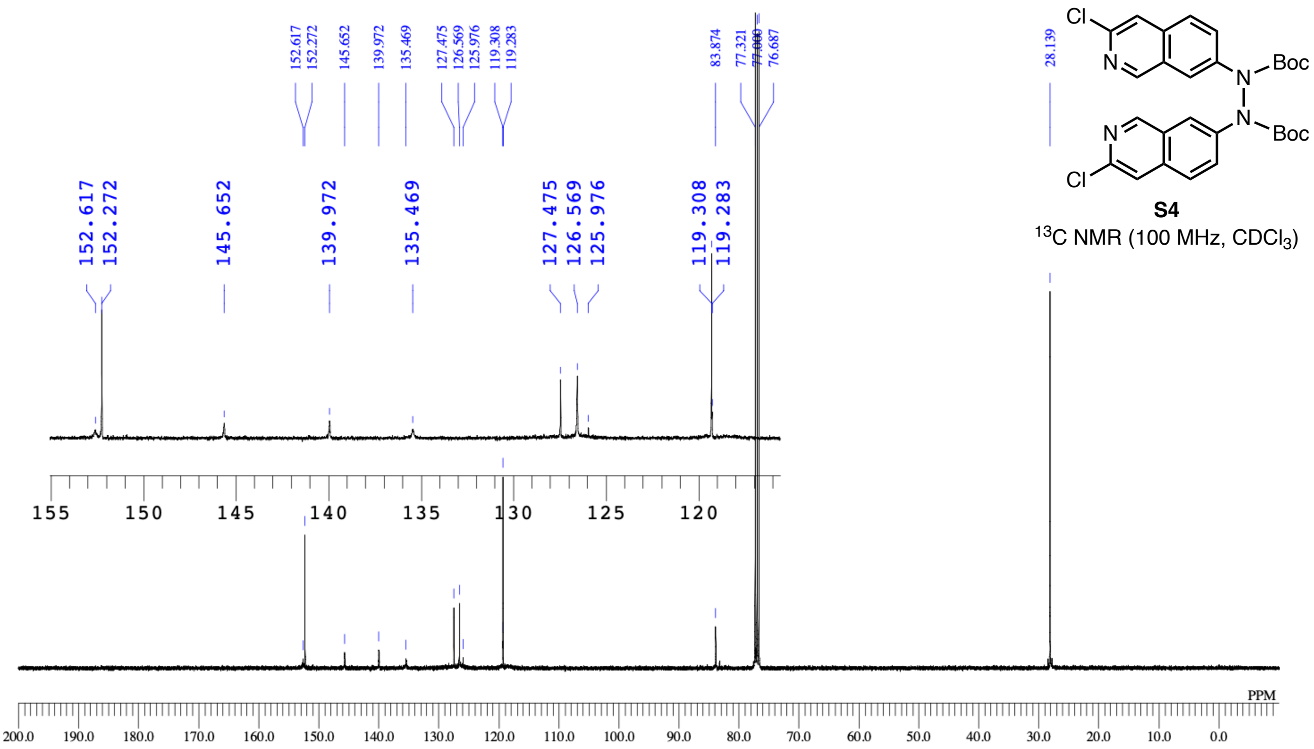
S4
13C NMR (100 MHz, CDCl3)
PPM

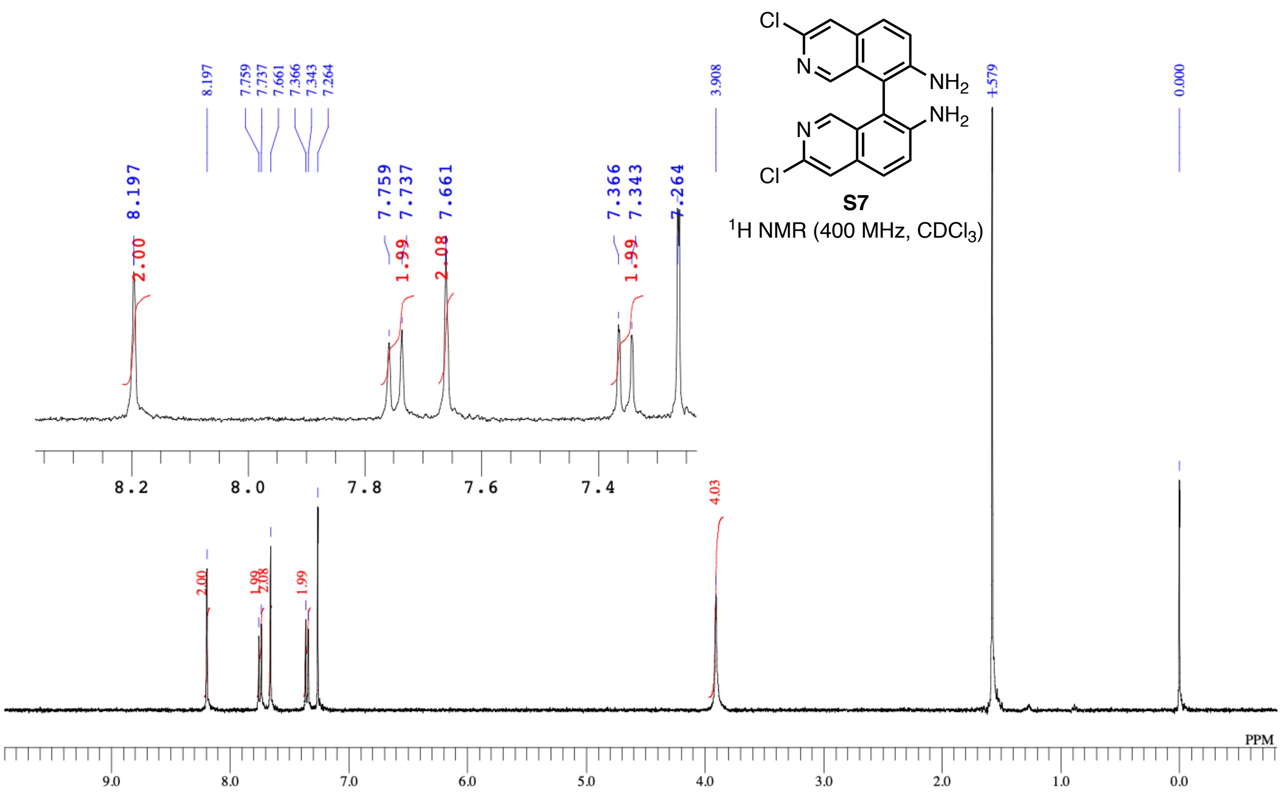
Cl
N
NH2
NH2
N
Cl
S7
1H NMR (400 MHz, CDCl3)
8.197
7.759
7.737
7.661
7.366
7.343
7.264
3.908
1.579
0.000
2.00
1.99
2.08
1.99
4.03
8.2
8.0
7.8
7.6
7.4
PPM
9.0
8.0
7.0
6.0
5.0
4.0
3.0
2.0
1.0
0.0

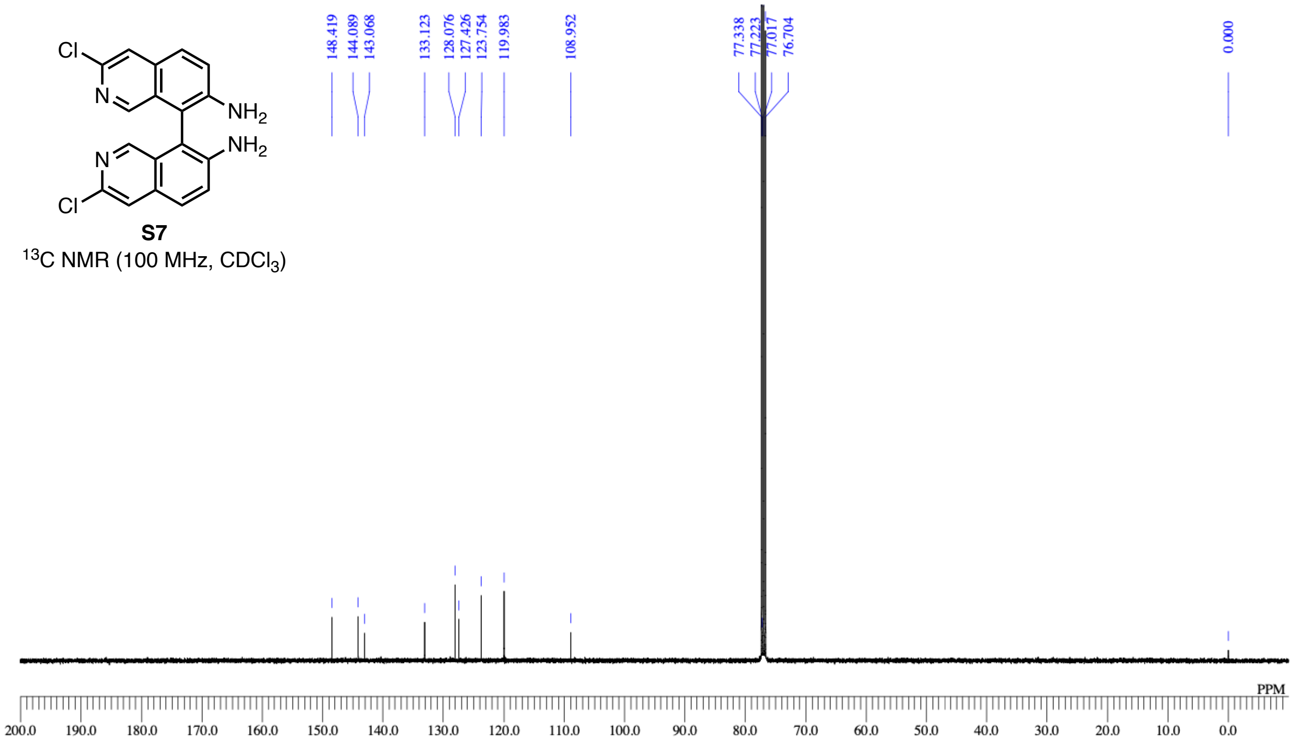
Cl
N
NH2
NH2
N
Cl
S7
13C NMR (100 MHz, CDCl3)
148.419
144.089
143.068
133.123
128.076
127.426
123.754
119.983
108.952
77.338
77.223
77.017
76.704
0.000
PPM
200.0
190.0
180.0
170.0
160.0
150.0
140.0
130.0
120.0
110.0
100.0
90.0
80.0
70.0
60.0
50.0
40.0
30.0
20.0
10.0
0.0

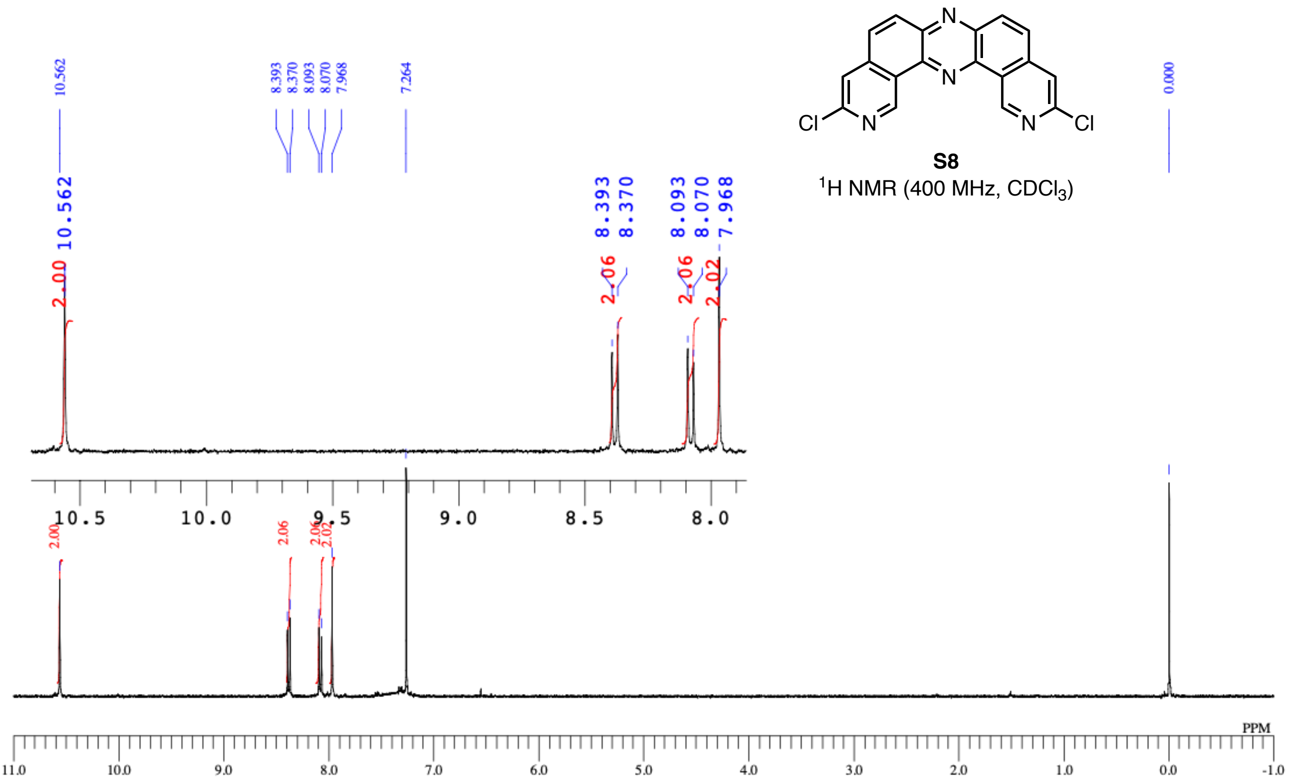

S8

^{1}H NMR (400 MHz, $CDCl_3$)

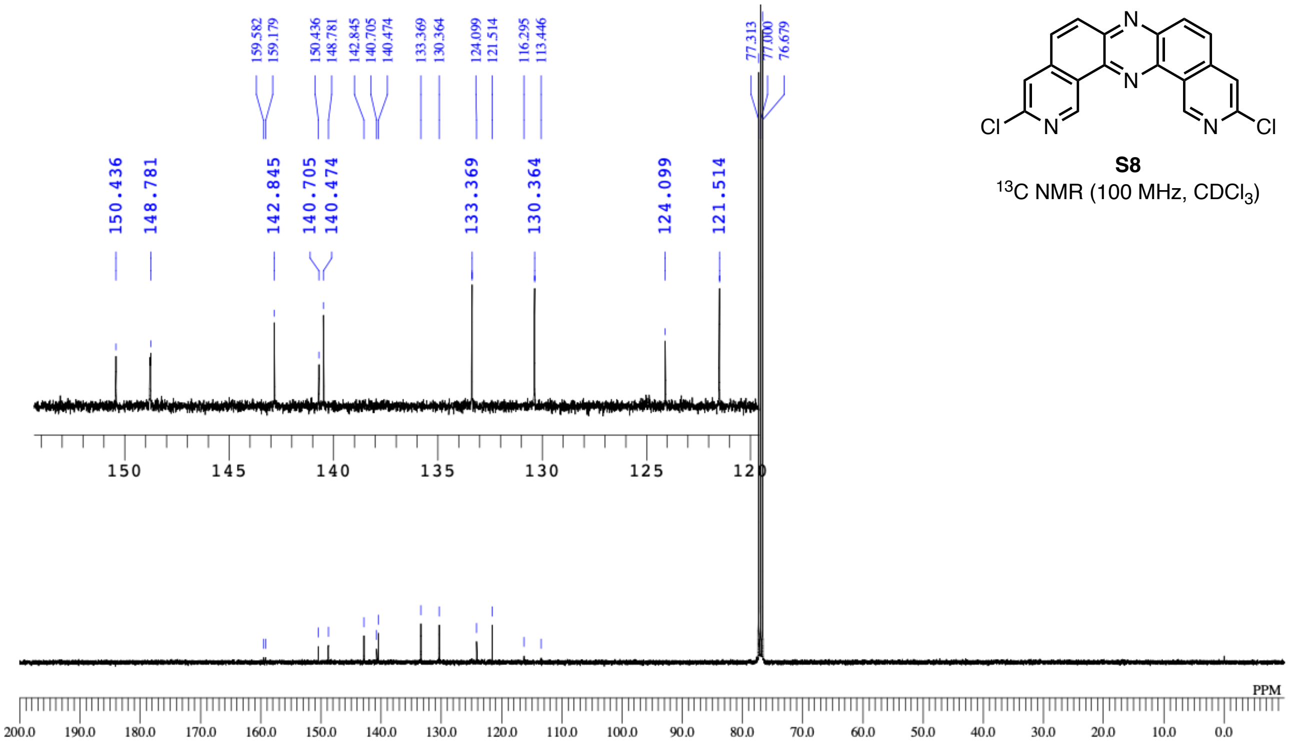

S8

^{13}C NMR (100 MHz, $CDCl_3$)

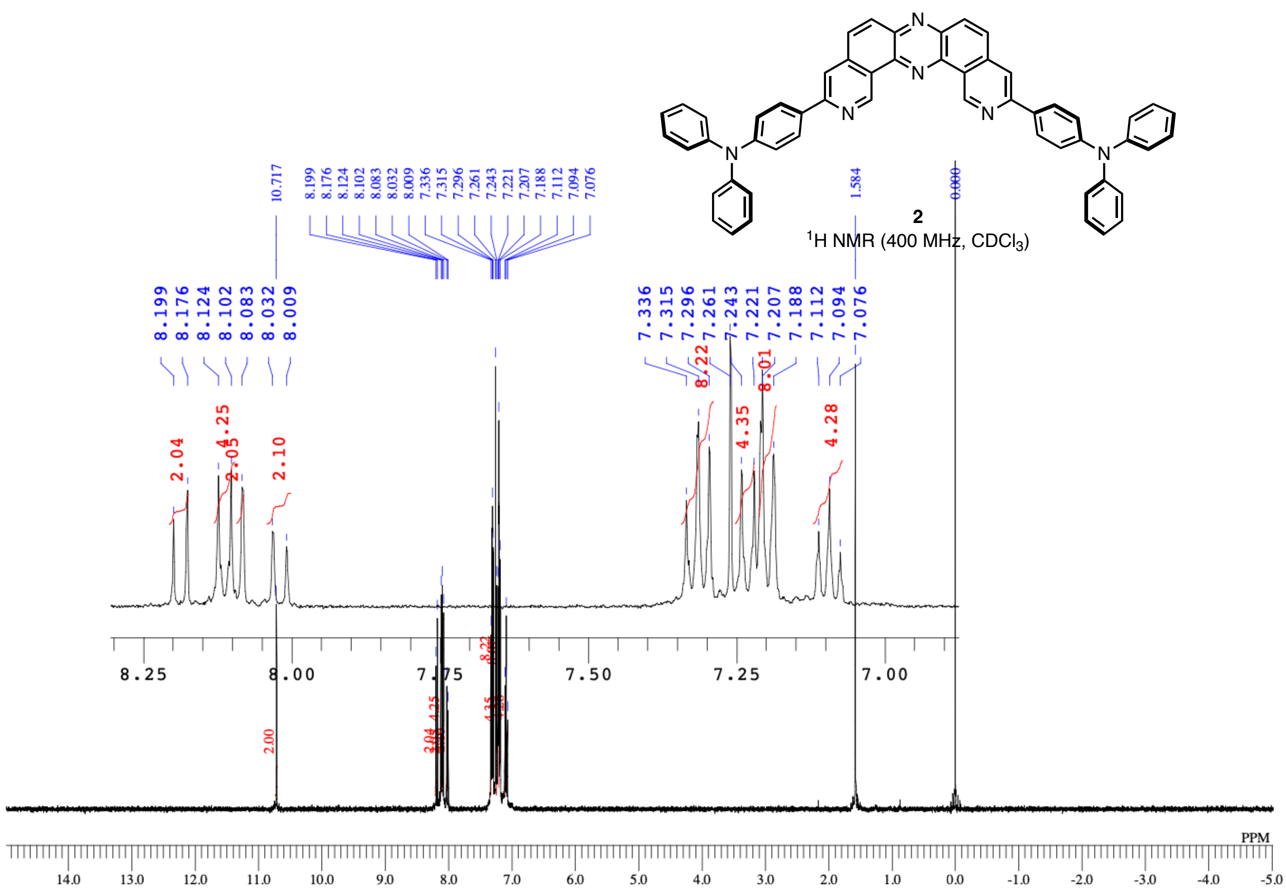
2
^{1}H NMR (400 MHz, $CDCl_3$)

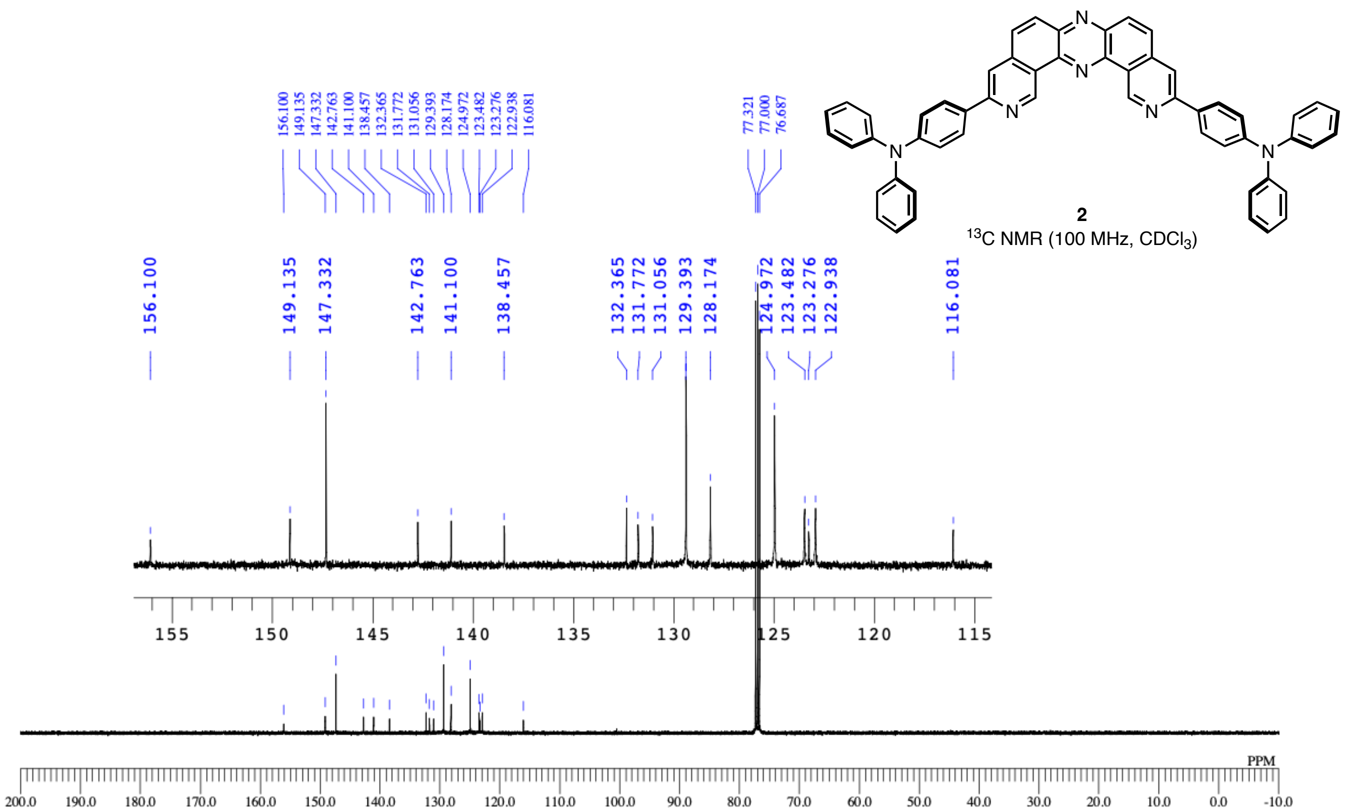
2
^{13}C NMR (100 MHz, $CDCl_3$)

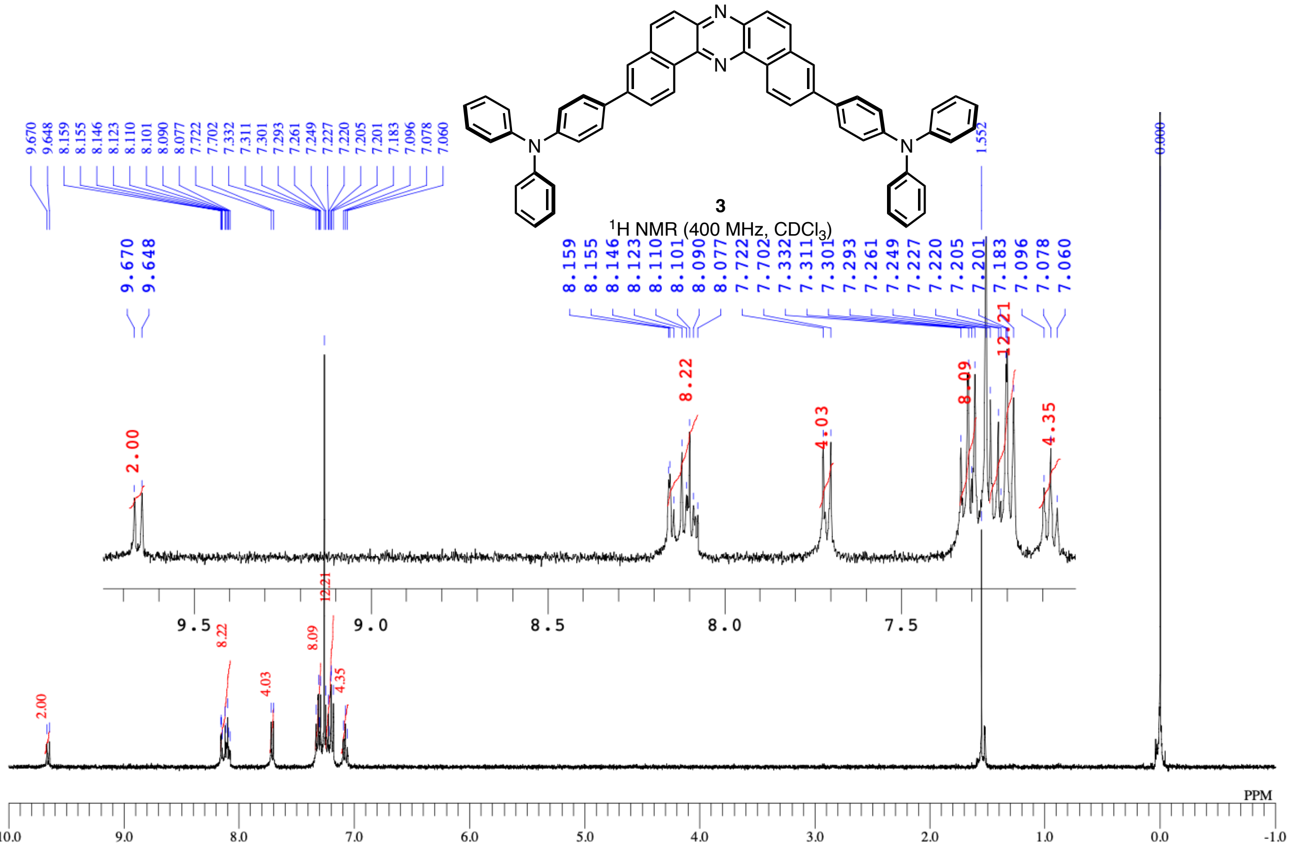

3
1H NMR (400 MHz, CDCl3)
9.670 9.648 8.159 8.155 8.146 8.123 8.110 8.101 8.090 8.077 7.722 7.702 7.332 7.311 7.301 7.293 7.261 7.249 7.227 7.220 7.205 7.201 7.183 7.096 7.078 7.060
1.552
0.000
2.00 8.22 4.03 8.09 12.21 4.35
PPM

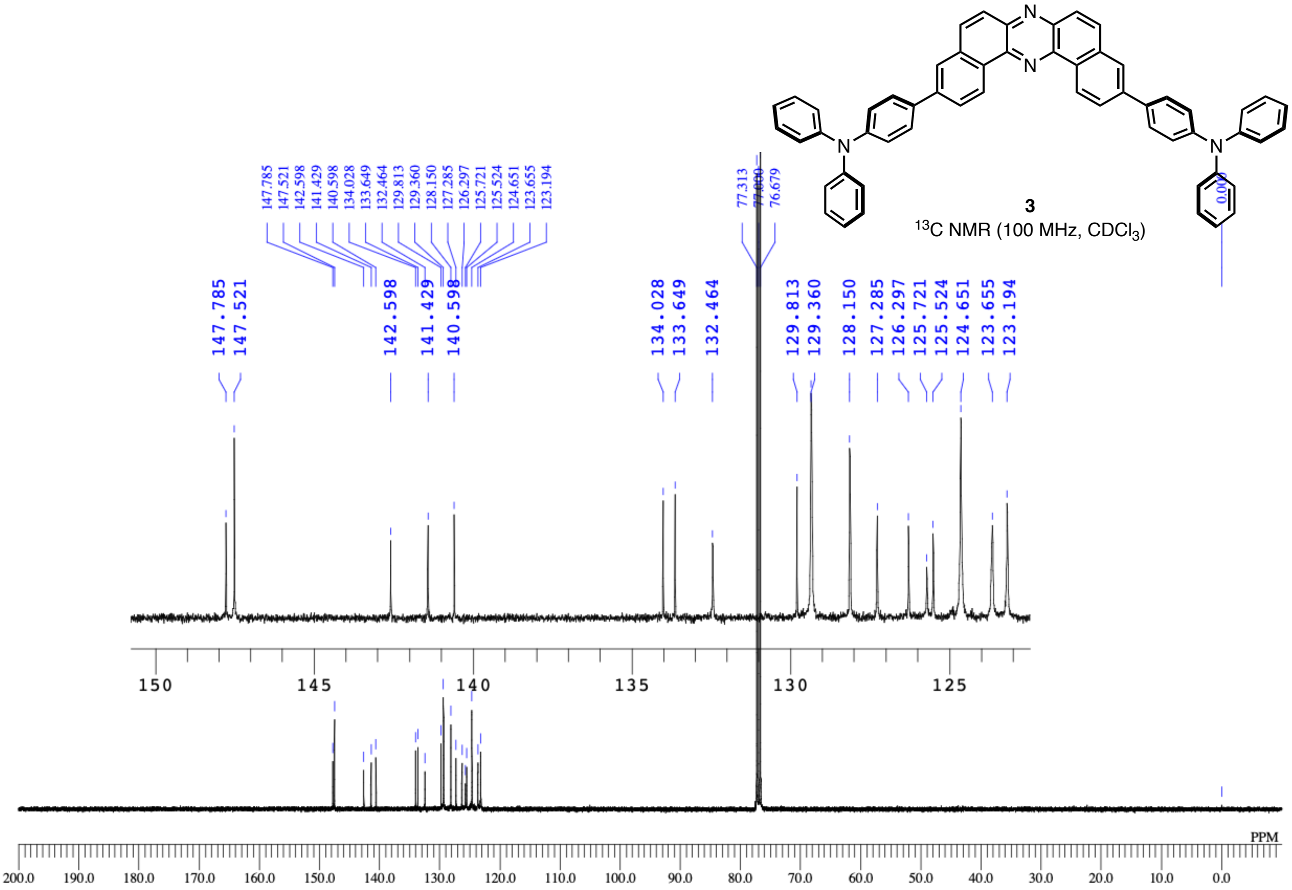

3
13C NMR (100 MHz, CDCl3)
147.785 147.521 142.598 141.429 140.598 134.028 133.649 132.464 129.813 129.360 128.150 127.285 126.297 125.721 125.524 124.651 123.655 123.194
77.313 77.000 76.679
0.000
PPM

References

[S1] Y. Takeda, M. Okazaki, S. Minakata, *Chem. Commun.* **2014**, *50*, 10291–10294.

[S2] L. E. de Sousa, P. de Silva, *J. Chem. Theory Comput.* **2021**, *17*, 5816–5824.

[S3] T. Stein, L. Kronik, R. Baer, *J. Am. Chem. Soc.* **2009**, *131*, 2818–2820.

[S4] For Gaussian 16, see: Gaussian 16, Revision C.01, M. J. Frisch, G. W. Trucks, H. B. Schlegel, G. E. Scuseria, M. A. Robb, J. R. Cheeseman, G. Scalmani, V. Barone, G. A. Petersson, H. Nakatsuji, X. Li, M. Caricato, A. V. Marenich, J. Bloino, B. G. Janesko, R. Gomperts, B. Mennucci, H. P. Hratchian, J. V. Ortiz, A. F. Izmaylov, J. L. Sonnenberg, D. Williams-Young, F. Ding, F. Lipparini, F. Egidi, J. Goings, B. Peng, A. Petrone, T. Henderson, D. Ranasinghe, V. G. Zakrzewski, J. Gao, N. Rega, G. Zheng, W. Liang, M. Hada, M. Ehara, K. Toyota, R. Fukuda, J. Hasegawa, M. Ishida, T. Nakajima, Y. Honda, O. Kitao, H. Nakai, T. Vreven, K. Throssell, J. A. Montgomery, Jr., J. E. Peralta, F. Ogliaro, M. J. Bearpark, J. J. Heyd, E. N. Brothers, K. N. Kudin, V. N. Staroverov, T. A. Keith, R. Kobayashi, J. Normand, K. Raghavachari, A. P. Rendell, J. C. Burant, S. S. Iyengar, J. Tomasi, M. Cossi, J. M. Millam, M. Klene, C. Adamo, R. Cammi, J. W. Ochterski, R. L. Martin, K. Morokuma, O. Farkas, J. B. Foresman and D. J. Fox, Gaussian, Inc., Wallingford CT, 2016.

[S5] For QChem, see: Y. Shao, Z. Gan, E. Epifanovsky, A. T. B. Gilbert, M. Wormit, J. Kussmann, A. W. Lange, A. Behn, J. Deng, X. Feng, D. Ghosh, M. Goldey P. R. Horn, L. D. Jacobson, I. Kaliman, R. Z. Khaliullin, T. Kús, A. Landau, J. Liu, E. I. Proynov, Y. M. Rhee, R. M. Richard, M. A. Rohrdanz, R. P. Steele, E. J. Sundstrom, H. L. Woodcock III, P. M. Zimmerman, D. Zuev, B. Albrecht, E. Alguire, B. Austin, G. J. O. Beran, Y. A. Bernard, E. Berquist, K. Brandhorst, K. B. Bravaya, S. T. Brown, D. Casanova, C.-M. Chang, Y. Chen, S. H. Chien, K. D. Closser, D. L. Crittenden, M. Diedenhofen, R. A. DiStasio Jr., H. Dop, A. D. Dutoi, R. G. Edgar, S. Fatehi, L. Fusti-Molnar, A. Ghysels, A. Golubeva-Zadorozhnaya, J. Gomes, M. W. D. HansonHeine, P. H. P. Harbach, A. W. Hauser, E. G. Hohenstein, Z. C. Holden, T.-C. Jagau, H. Ji, B. Kaduk, K. Khistyaev, J. Kim, J. Kim, R. A. King, P. Klunzinger, D. Kosenkov, T. Kowalczyk, C. M. Krauter, K. U. Lao, A. Laurent, K. V. Lawler, S. V. Levchenko, C. Y. Lin, F. Liu, E. Livshits, R. C. Lochan, A. Luenser, P. Manohar, S. F. Manzer, S.-P. Mao, N. Mardirossian, A. V. Marenich, S. A. Maurer, N. J. Mayhall, C. M. Oana, R. Olivares-Amaya, D. P. O'Neill, J. A. Parkhill, T. M. Perrine, R. Peverati, P. A. Pieniazek, A. Prociuk, D. R. Rehn, E. Rosta, N. J. Russ, N. Sergueev, S. M. Sharada, S. Sharmaa, D. W. Small, A. Sodt, T. Stein, D. Stück, Y.-C. Su, A. J. W. Thom, T. Tsuchimochi, L. Vogt, O. Vydrov, T. Wang, M. A. Watson, J. Wenzel, A. White, C. F. Williams, V. Vanovschi, S. Yeganeh, S. R. Yost, Z.-Q. You, I. Y. Zhang, X. Zhang, Y. Zhou, B. R. Brooks, G. K. L. Chan, D. M. Chipman, C. J. Cramer, W.

A. Goddard III, M. S. Gordon, W. J. Hehre, A. Klamt, H. F. Schaefer III, M. W. Schmidt, C. D. Sherrill, D. G. Truhlar, A. Warshel, X. Xua, A. Aspuru-Guzik, R. Baer, A. T. Bell, N. A. Besley, J.-D. Chai, A. Dreuw, B. D. Dunietz, T. R. Furlani, S. R. Gwaltney, C.-P. Hsu, Y. Jung, J. Kong, D. S. Lambrecht, W. Liang, C. Ochsenfeld, V. A. Rassolov, L. V. Slipchenko, J. E. Subotnik, T. Van Voorhis, J. M. Herbert, A. I. Krylov, P. M. W. Gill and M. Head-Gordon, Molecular Phys., 2015, 113, 184–215.

[S6] Y.-K. Lim, J.-W. Jung, H. Lee, C.-G. Cho, *J. Org. Chem.* **2004**, *69*, 5778–5781.

[S7] N. D. Adams, A. M. Chaudhari, T. J. Kiesow, C. A. Parrish, A. J. Reif, L. H. Ridgers, S. J. Schmidt, K. Wiggall, WO 2012/064642 A1.

[S8] Rigaku Oxford Diffraction (2015), Software CrysAlisPro 1.171.39.20a Rigaku Corporation, Tokyo, Japan.

[S9] G. M. Sheldrick, G *Acta Cryst*. **2015**, *A71*, 3-8.

[S10] O. V. Dolomanov, L. J. Bourhis, R. J. Gildea, J. A. K. Howard, H. Puschmann, *J. Appl. Cryst.* **2009**, *42*, 339–341.

[S11] G. M. Sheldrick, *Acta Cryst.* **2015**, *C71*, 3–8.

[S12] Rigaku Oxford Diffraction (2024, Software CrysAlisPro 1.171.43.105a Rigaku Corporation, Tokyo, Japan.

[S13] http://app.supramolecular.org/bindfit/
